# Supplementary material for: Population structure, biogeography and transmissibility of Mycobacterium tuberculosis
Source: Nat Commun. 2021 Oct 20;12:6099. doi: 10.1038/s41467-021-26248-1 (PMC8528816; doi:10.1038/s41467-021-26248-1)
Supplement: Supplementary file 1 — Supplementary Information [file 41467_2021_26248_MOESM1_ESM.pdf]

Supplementary Information

Population structure, biogeography and transmissibility of *Mycobacterium tuberculosis*

Luca Freschi, Roger Vargas Jr., Ashaque Husain, S M Mostofa Kamal, Alena Skrahina, Sabira Tahseen, Nazir Ismail, Anna Barbova, Stefan Niemann, Daniela Maria Cirillo, Anna S Dean, Matteo Zignol and Maha Reda Farhat

Supplementary Figures

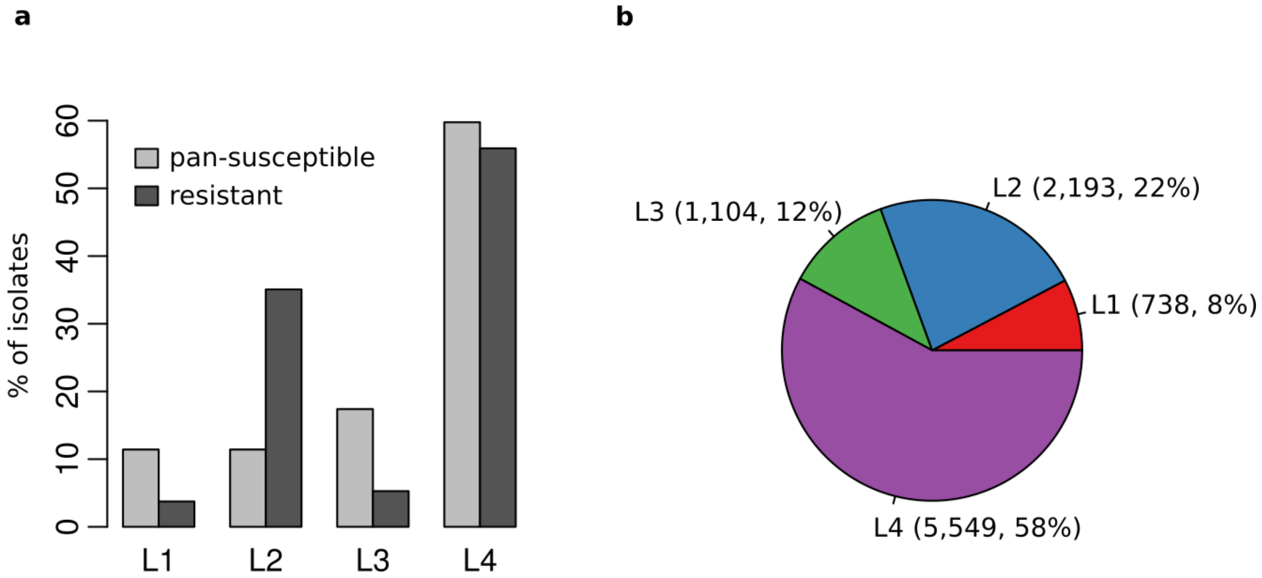

Suppl. Figure 1. Composition of the dataset of isolates with known phenotypic data by lineage and antibiotic resistance susceptibility. **a** Bar chart showing the percentage of pan-susceptible or resistant isolates for each one of the four major *Mtb* lineages (Lineage 1: 564 pan-susceptible and 174 resistant isolates; Lineage 2: 564 pan-susceptible and 1,629 resistant isolates; Lineage 3: 859 pan-susceptible and 245 resistant isolates; Lineage 4: 2,952 pan-susceptible and 2,597 resistant isolates). **b** Pie chart showing the number of isolates present in our dataset for each one of the four major *Mtb* lineages (L1-4). Percentages relative to the total number of isolates of our dataset are also shown. Source data are provided as a Source Data file.

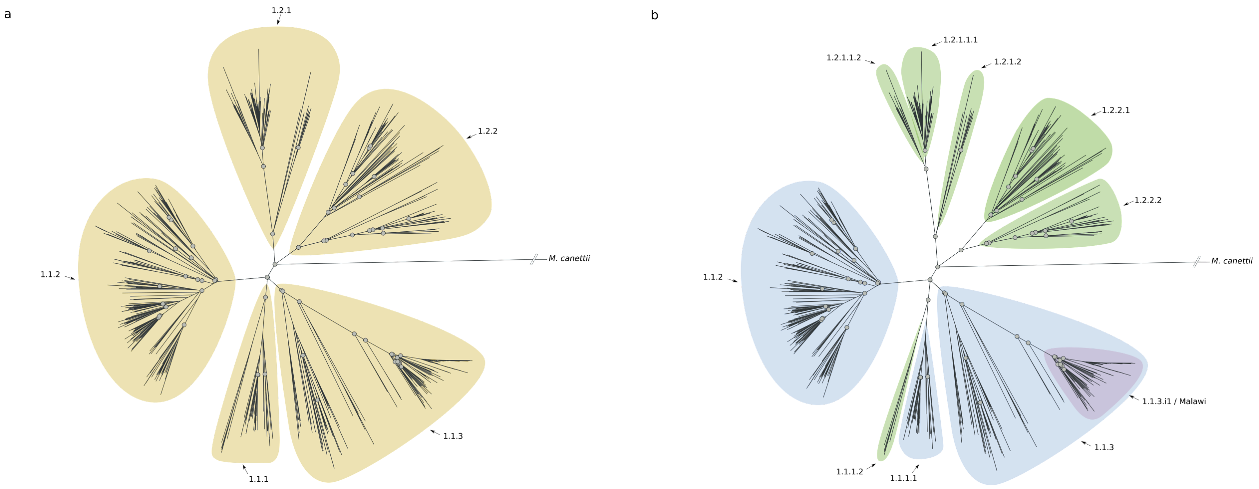

Suppl. Figure 2. Sub-lineage definitions for lineage 1 according to *Coll et al.*[1] *or as described in this study.* **a** Phylogenetic tree reconstruction of lineage 1. Yellow areas define sub-lineages of lineage 1 as described by *Coll et al.*[1] **b** Phylogenetic tree reconstruction of lineage 1. Colored areas define sub-lineages of lineage 1 as described in this study (blue: sub-lineages that match those already described in the literature; green: sub-lineages described here; purple: internal sub-lineages). Source data are provided as a Source Data file.

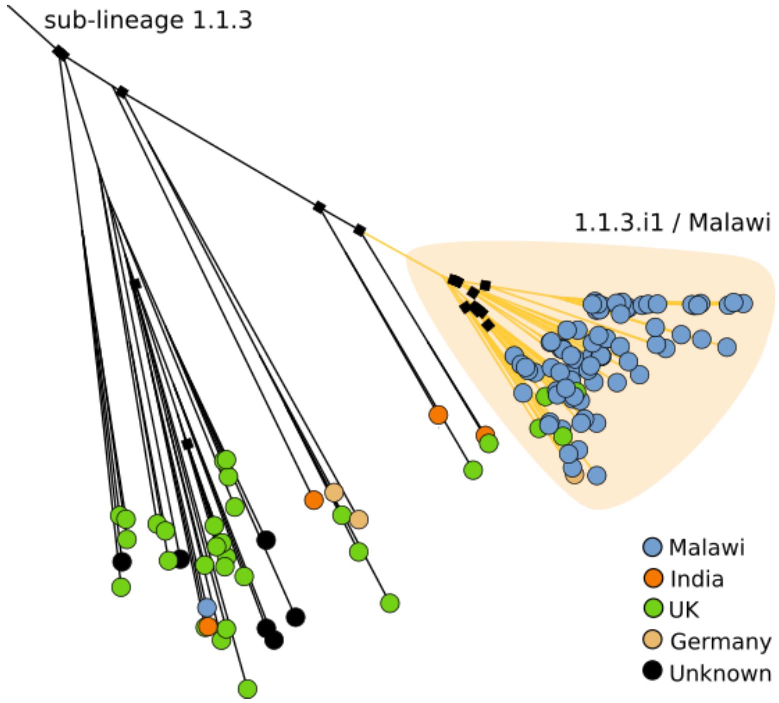

Suppl. Figure 3. Phylogenetic context of the internal sub-lineage 1.1.3.i1 / Malawi. The picture shows a magnification of a portion of lineage 1 phylogeny that includes sub-lineage 1.1.3. The yellow area defines the internal sub-lineage 1.1.3.i1 / Malawi. Black squares represent splits where the FST (fixation index) calculated using the descendants of the two children nodes is greater than 0.33. Colored circles represent single isolates and provide information about the country where each were sampled. Source data are provided as a Source Data file.

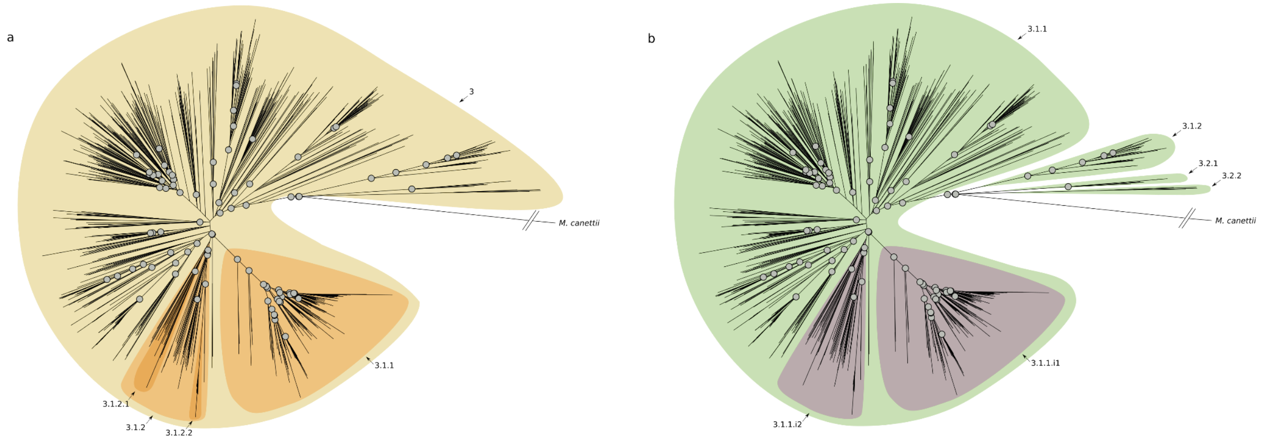

Suppl. Figure 4. Sub-lineage definitions for lineage 3 according to *Coll et al.*[1] *or as described in this study.* a Phylogenetic tree reconstruction of lineage 3. Yellow and orange areas define sub-lineages of L3 described by Coll *et al.*[1] ***b*** Phylogenetic tree reconstruction of L3. Colored areas define sub-lineages of lineage 3 as described in this study (blue: sub-lineages that match those already described in the literature; green: sub-lineages described here; purple: internal sub-lineages). Source data are provided as a Source Data file.

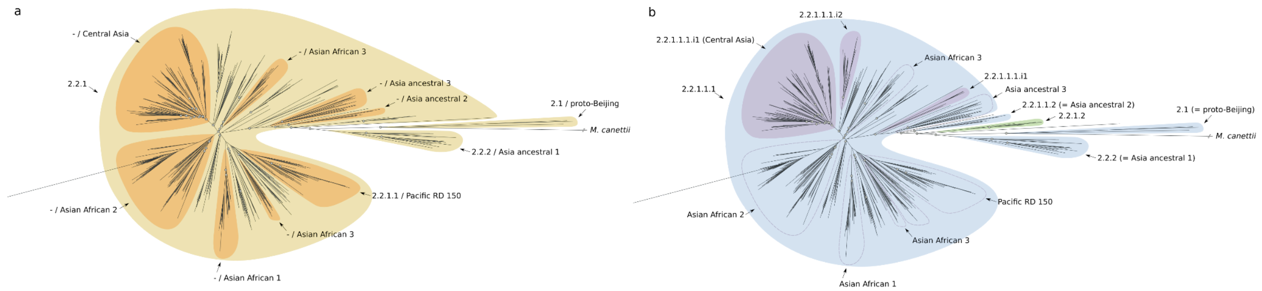

Suppl. Figure 5. Sub-lineage definitions for lineage 2 according to *Coll et al.*[1] *or as described in this study.* a Phylogenetic tree reconstruction of lineage 2. Yellow and orange areas define sub-lineages of lineage 2 described by Coll *et al.*[1] or Shitikov *et al.*[2] ***b*** Phylogenetic tree reconstruction of lineage . Colored areas define sub-lineages of lineage 2 as described in this study (blue: sub-lineages that match those already described in the literature; green: sub-lineages described here; purple: internal sub-lineages). Source data are provided as a Source Data file.

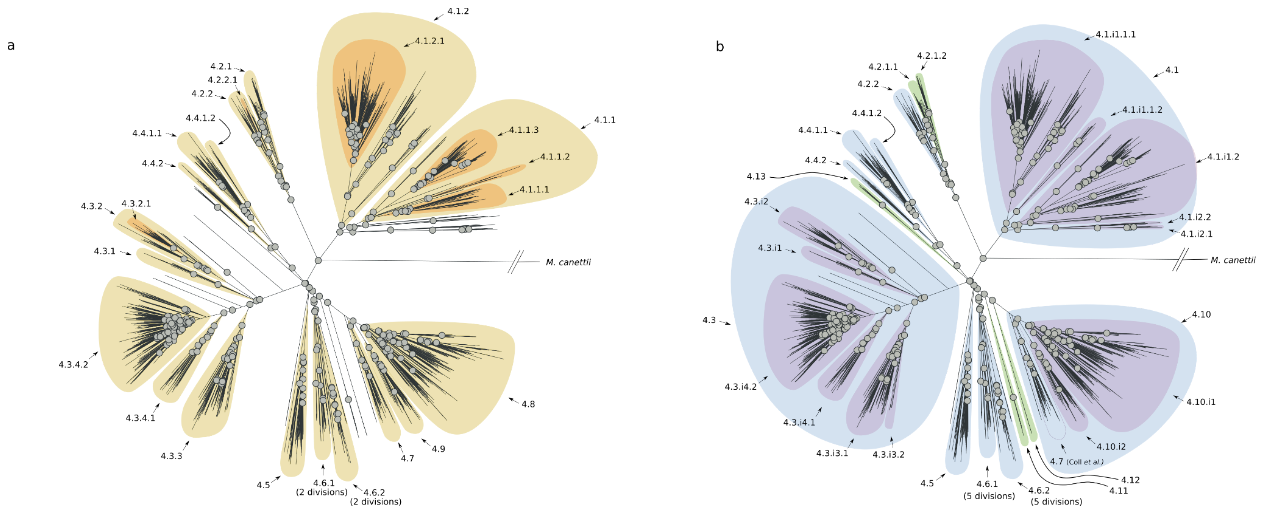

Suppl. Figure 6. Sub-lineage definitions for lineage 4 according to *Coll et al.* [1] ***or as described in this study.* a** Phylogenetic tree reconstruction of lineage 4. Yellow areas define sub-lineages of lineage 4 described by Coll *et al.* [1] ***b*** Phylogenetic tree reconstruction of lineage 4. Colored areas define sub-lineages of lineage 4 as described in this study (blue: sub-lineages that match those already described in the literature; green: sub-lineages described here; purple: internal sub-lineages). Source data are provided as a Source Data file.

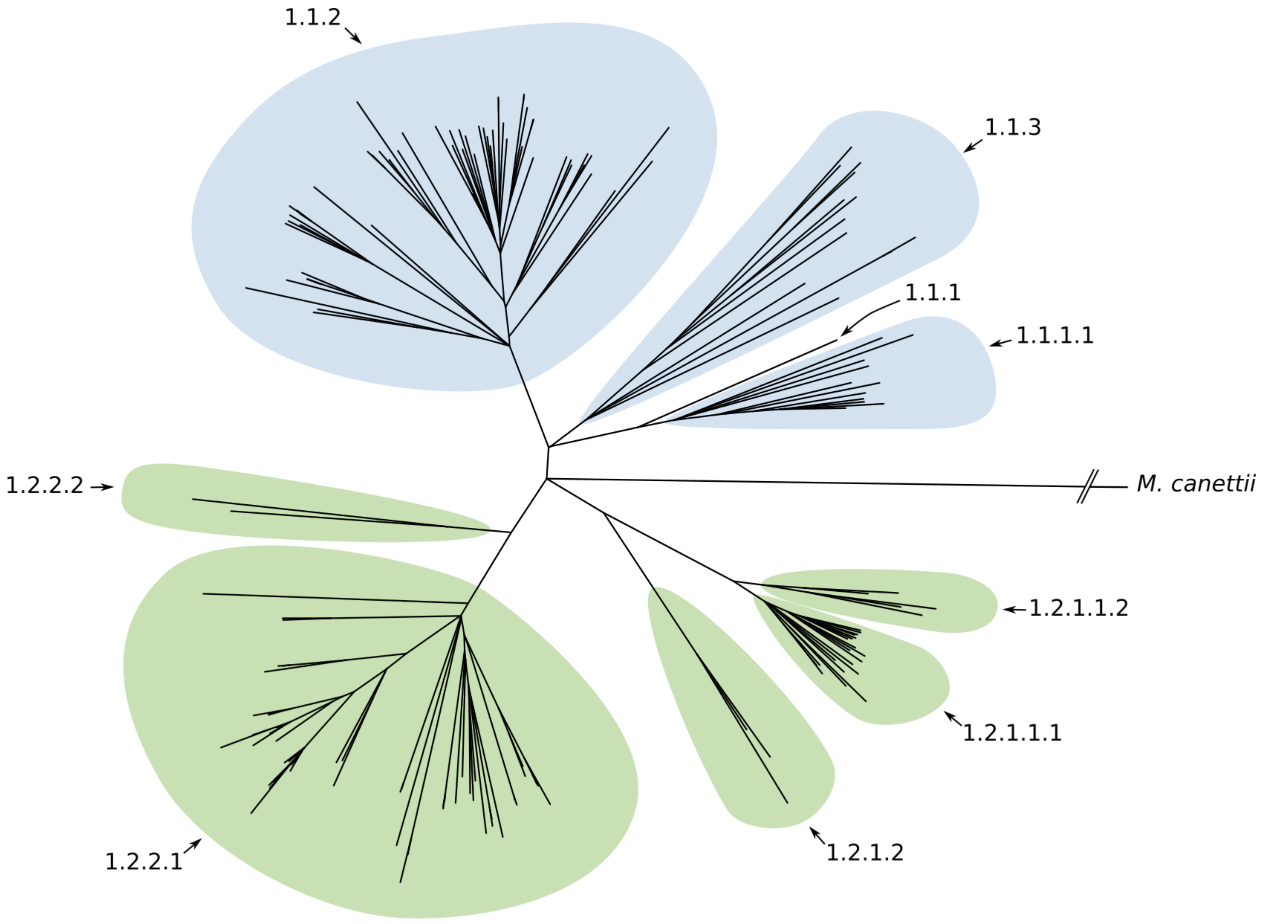

Suppl. Figure 7. Phylogenetic tree reconstruction of lineage 1 resistant isolates (binary tree). Colored areas define sub-lineages of lineage 1 as described in this study (blue: sub-lineages that match those already described in the literature; green: sub-lineages described here; purple: internal sub-lineages). Source data are provided as a Source Data file.

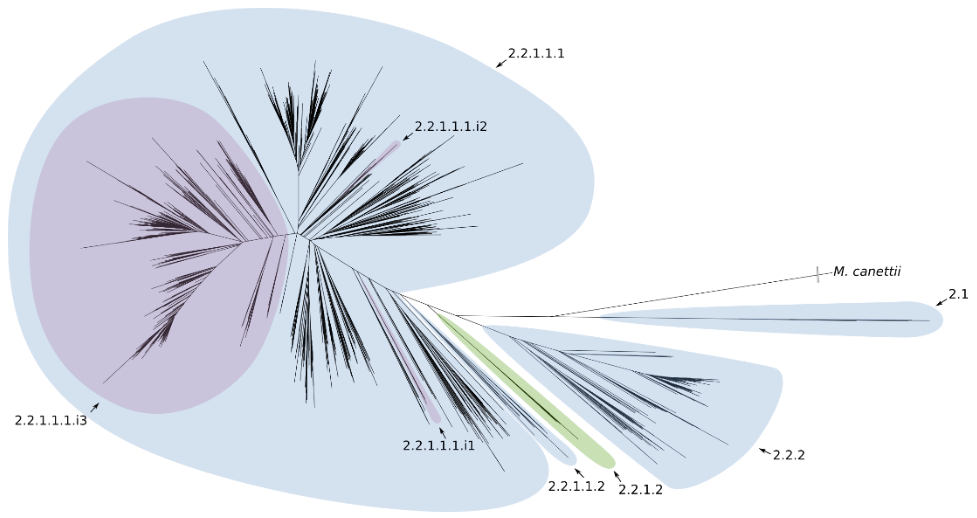

Suppl. Figure 8. Phylogenetic tree reconstruction of lineage 2 resistant isolates (binary tree). Colored areas define sub-lineages of lineage 2 as described in this study (blue: sub-lineages that match those already described in the literature; green: sub-lineages described here; purple: internal sub-lineages). Source data are provided as a Source Data file.

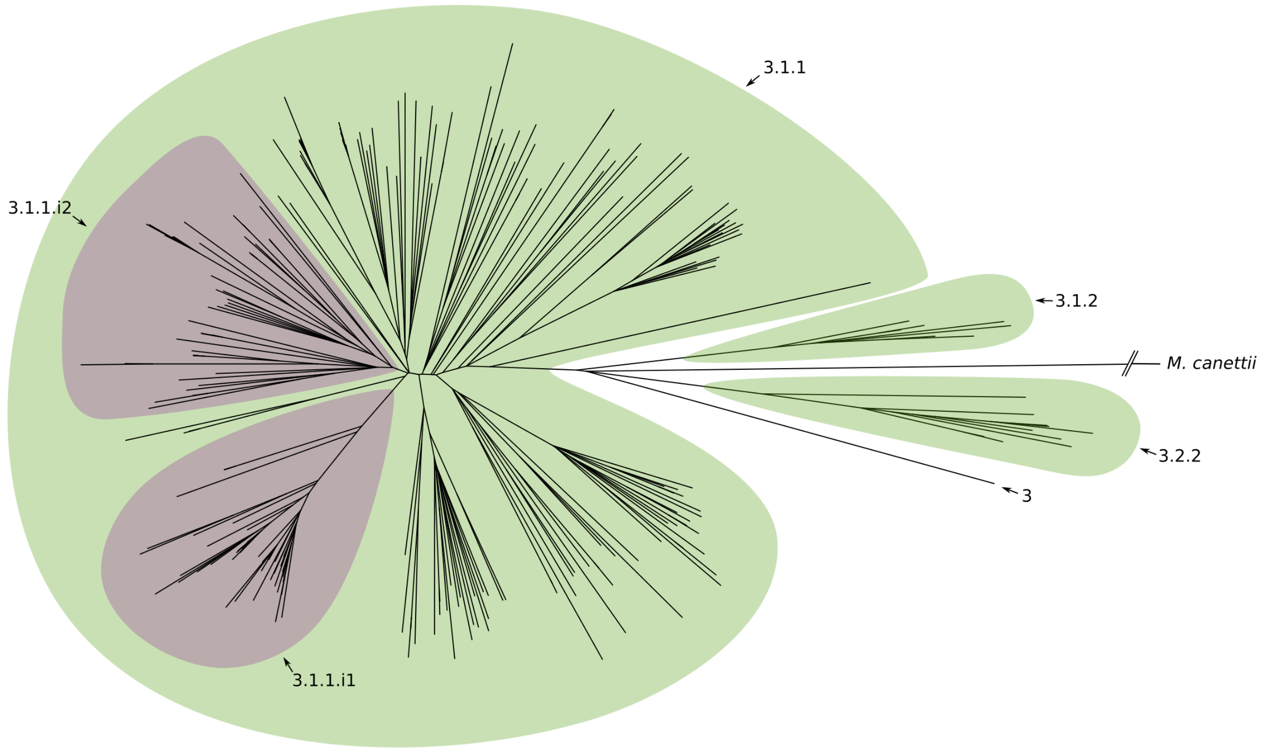

Suppl. Figure 9. Phylogenetic tree reconstruction of lineage 3 resistant isolates (binary tree). Colored areas define sub-lineages of lineage 3 as described in this study (blue:

sub-lineages that match those already described in the literature; green: sub-lineages described here; purple: internal sub-lineages). Source data are provided as a Source Data file.

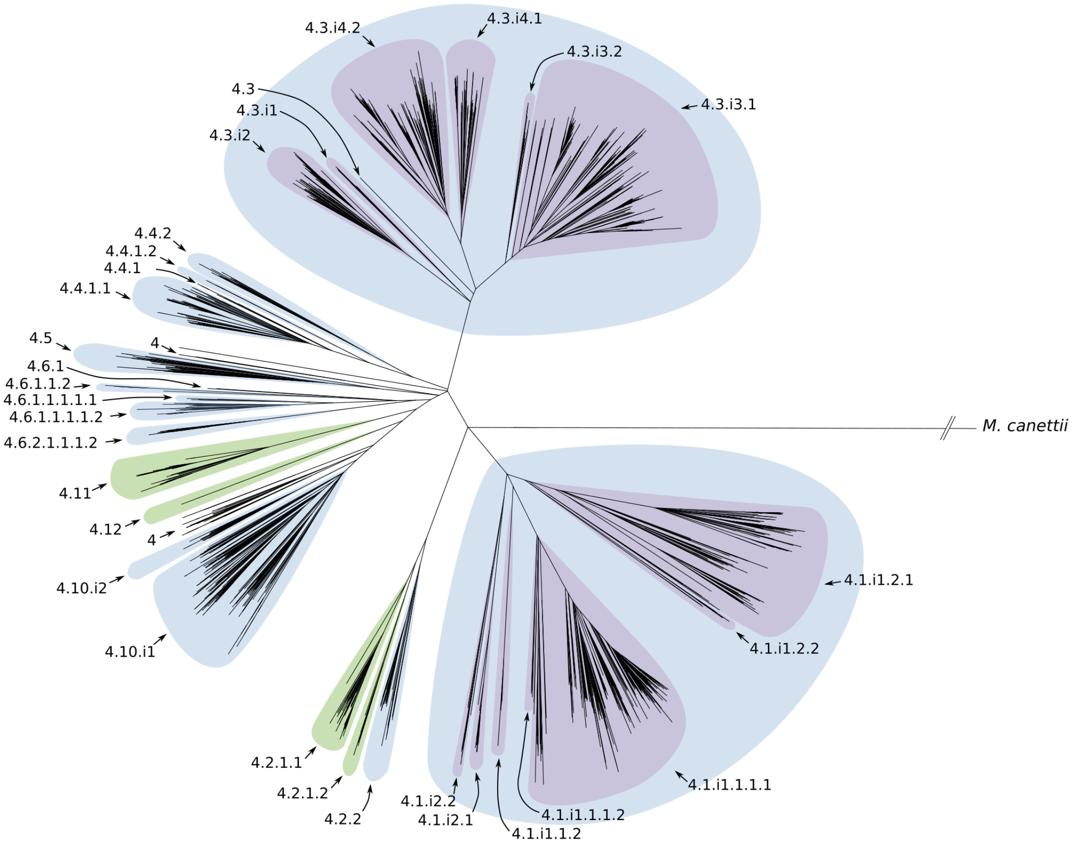

Suppl. Figure 10. Phylogenetic tree reconstruction of lineage 4 resistant isolates (binary tree). Colored areas define sub-lineages of lineage 4 as described in this study (blue: sub-lineages that match those already described in the literature; green: sub-lineages described here; purple: internal sub-lineages). Source data are provided as a Source Data file.

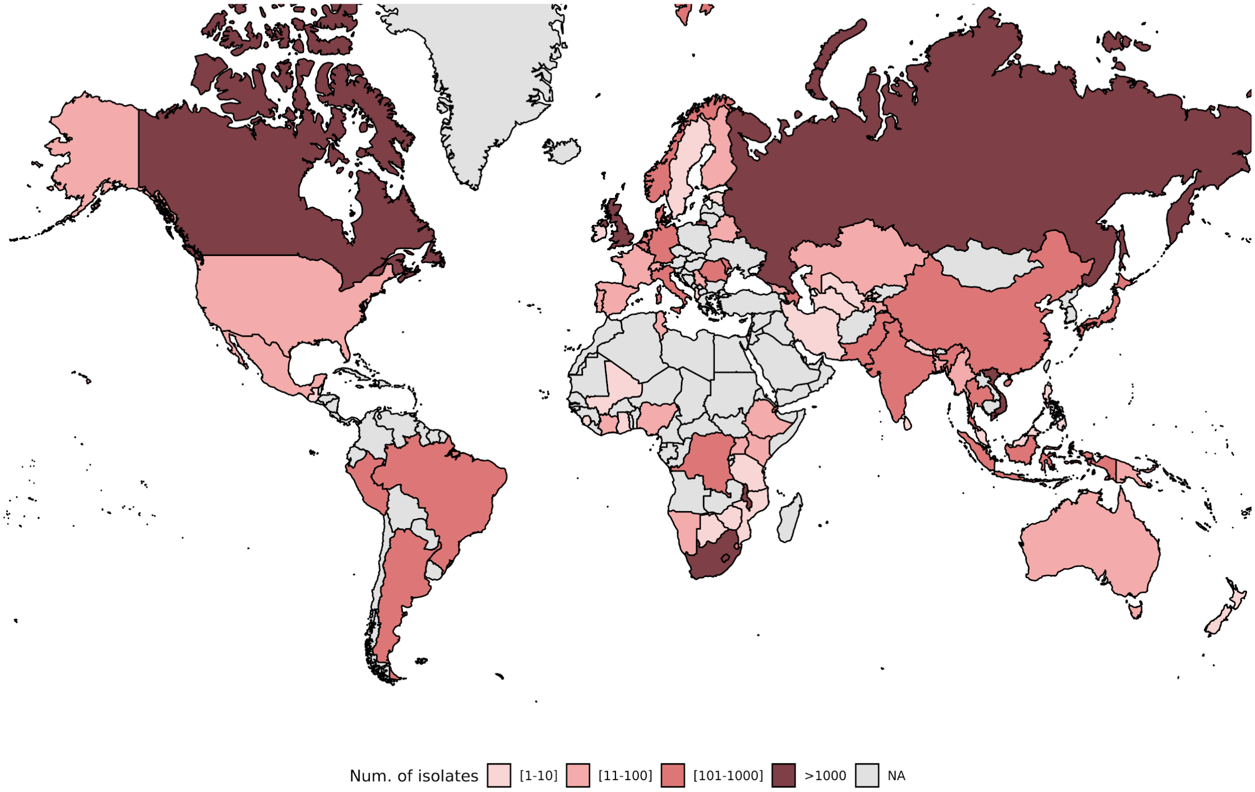

Suppl. Figure 11. Geographic distribution of the dataset of isolates used to study the biogeography of *Mtb* sub-lineages. Source data are provided as a Source Data file.

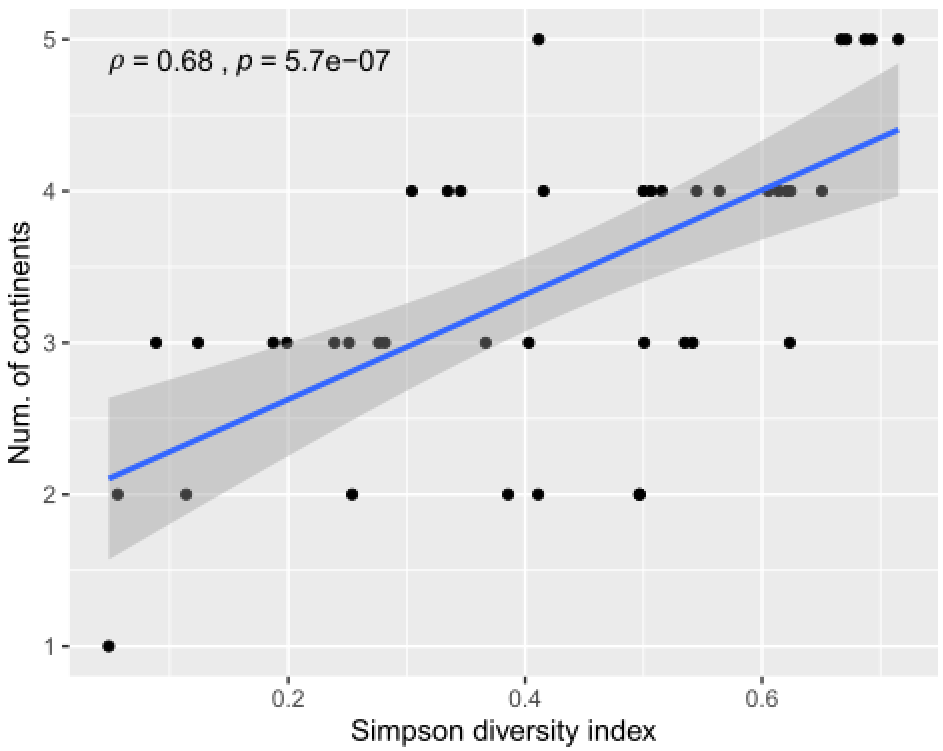

Suppl. Figure 12. Relationship between the Simpson diversity index and the number of continents where a given sub-lineage has been found. The blue line shows a linear regression line and the grey area shows the 95% confidence interval. Spearman's rank correlation coefficient and the associated p-value (two-tailed test) are shown in the top-left part of the plot. Source data are provided as a Source Data file.

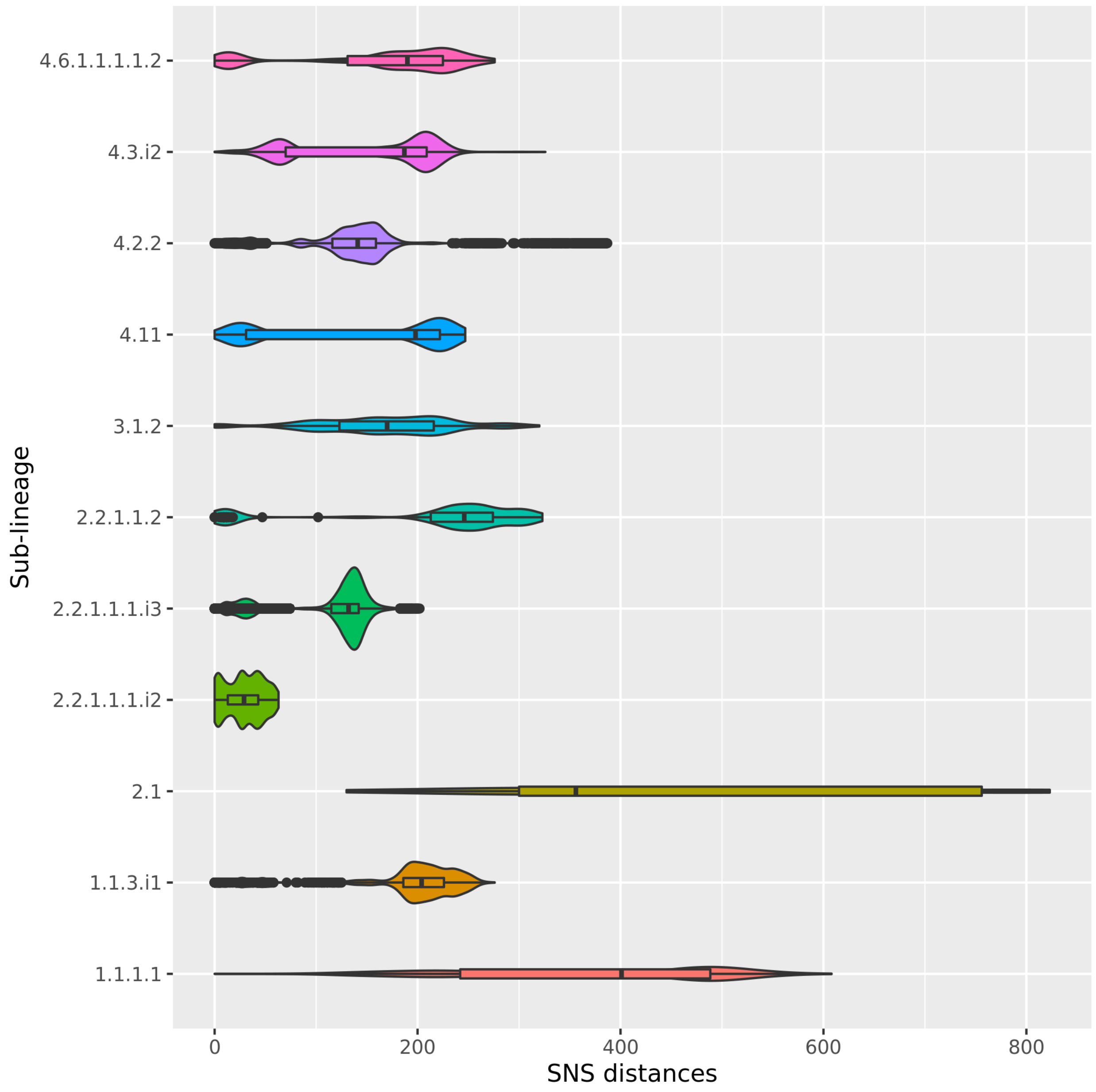

Suppl. Figure 13. Distributions of the pairwise SNS distances of the sub-lineages / internal groups that had Simpson diversity index < 0.28. Colors indicate different sub-lineages, which are listed on the y axis. Description of the distributions (lineage 1.1.1.1: n = 903, Min: 0, 1st Quartile: 242, Median: 401, 3rd Quartile: 488.5, Max: 608; lineage 1.1.3.i1: n = 4095, Min: 0, 1st Quartile: 186, Median: 204, 3rd Quartile: 226, Max: 276; lineage 2.1: n = 21, Min: 130, 1st Quartile: 300, Median: 356, 3rd Quartile: 756, Max: 823; lineage 2.2.1.1.1.i2: n = 820, Min: 0, 1st Quartile: 13, Median: 29, 3rd Quartile: 43, Max: 63; lineage 2.2.1.1.1.i3: n = 303810, Min: 0, 1st Quartile: 115, Median: 132, 3rd Quartile: 142, Max: 202; lineage 2.2.1.1.2: n = 300, Min: 0, 1st Quartile: 213, Median: 246, 3rd Quartile: 274, Max: 323; lineage 3.1.2: n = 990, Min: 0, 1st Quartile: 123, Median: 170, 3rd Quartile: 216, Max: 320; lineage 4.11: n = 4656,

Min: 0, 1st Quartile: 31, Median: 198, 3rd Quartile: 222, Max: 247; lineage 4.2.2: n = 7626, Min: 0, 1st Quartile: 116, Median: 141, 3rd Quartile: 159, Max: 387; lineage 4.3.i2: n = 28203, Min: 0, 1st Quartile: 70, Median: 187, 3rd Quartile: 209, Max: 326; lineage 4.6.1.1.1.1.2: n = 1830, Min: 0, 1st Quartile: 131, Median: 190, 3rd Quartile: 225, Max: 276). Source data are provided as a Source Data file.

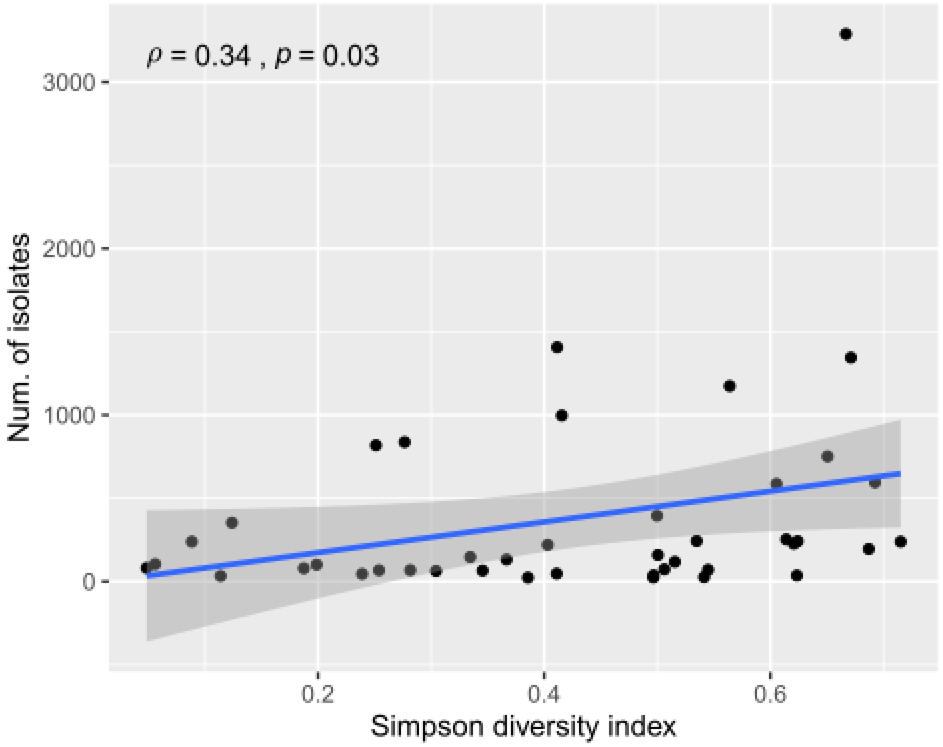

Suppl. Figure 14. Relationship between the Simpson diversity index and the number of isolates of each sub-lineage. The blue line shows a linear regression line and the grey area shows the 95% confidence interval. Spearman's rank correlation coefficient and the associated p-value (two-tailed test) are shown in the top-left part of the plot. Source data are provided as a Source Data file.

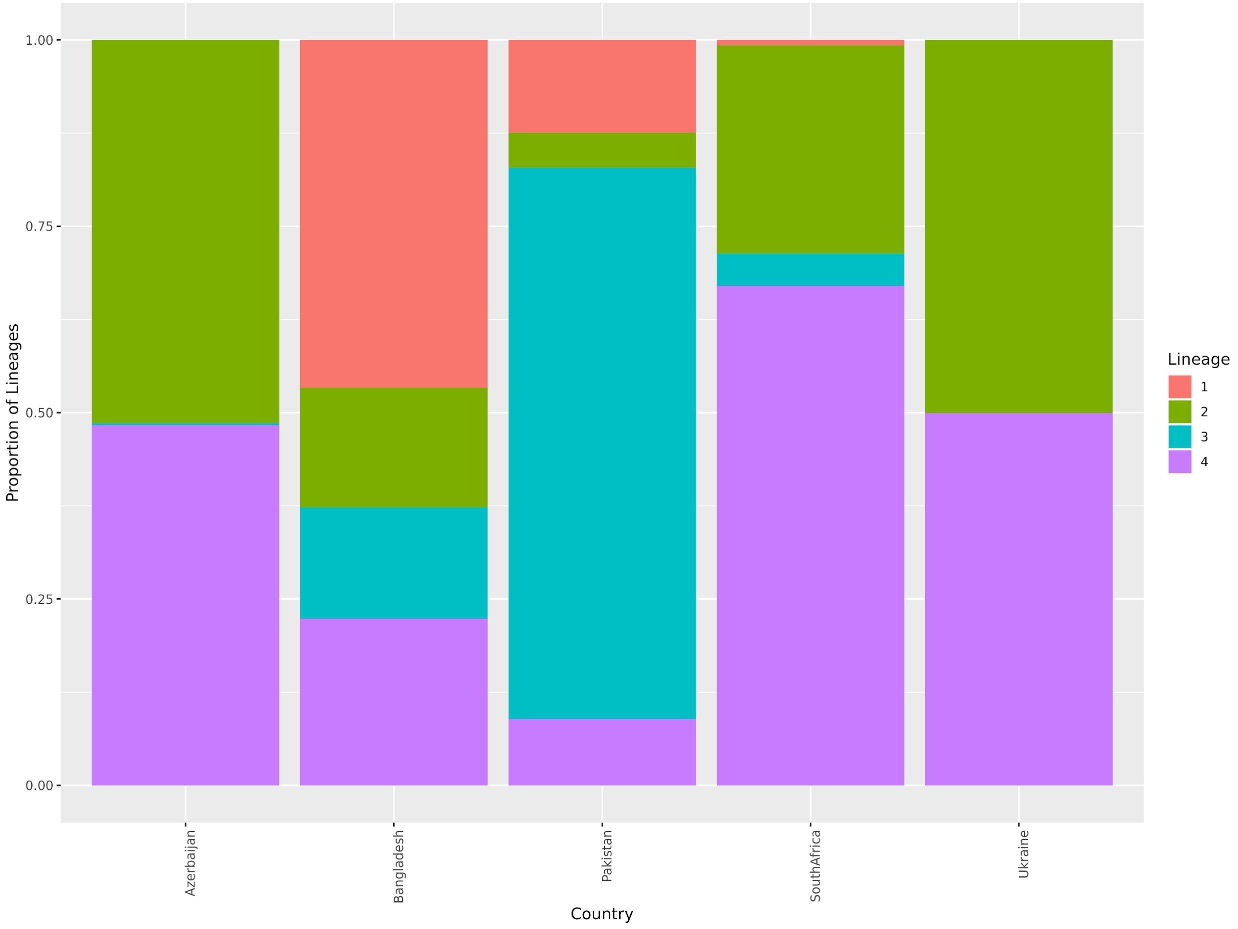

Suppl. Figure 15. Prevalence of the four major Mtb lineages in five countries where isolates were randomly sampled. Colors indicate different sub-lineages (red: lineage 1, green: lineage 2, teal: lineage 3, purple: lineage 4). Dataset: 704 isolates from Azerbaijan, 555 from Bangladesh, 169 from Pakistan, 1206 from South Africa and 1033 from Ukraine. Source data are provided as a Source Data file.

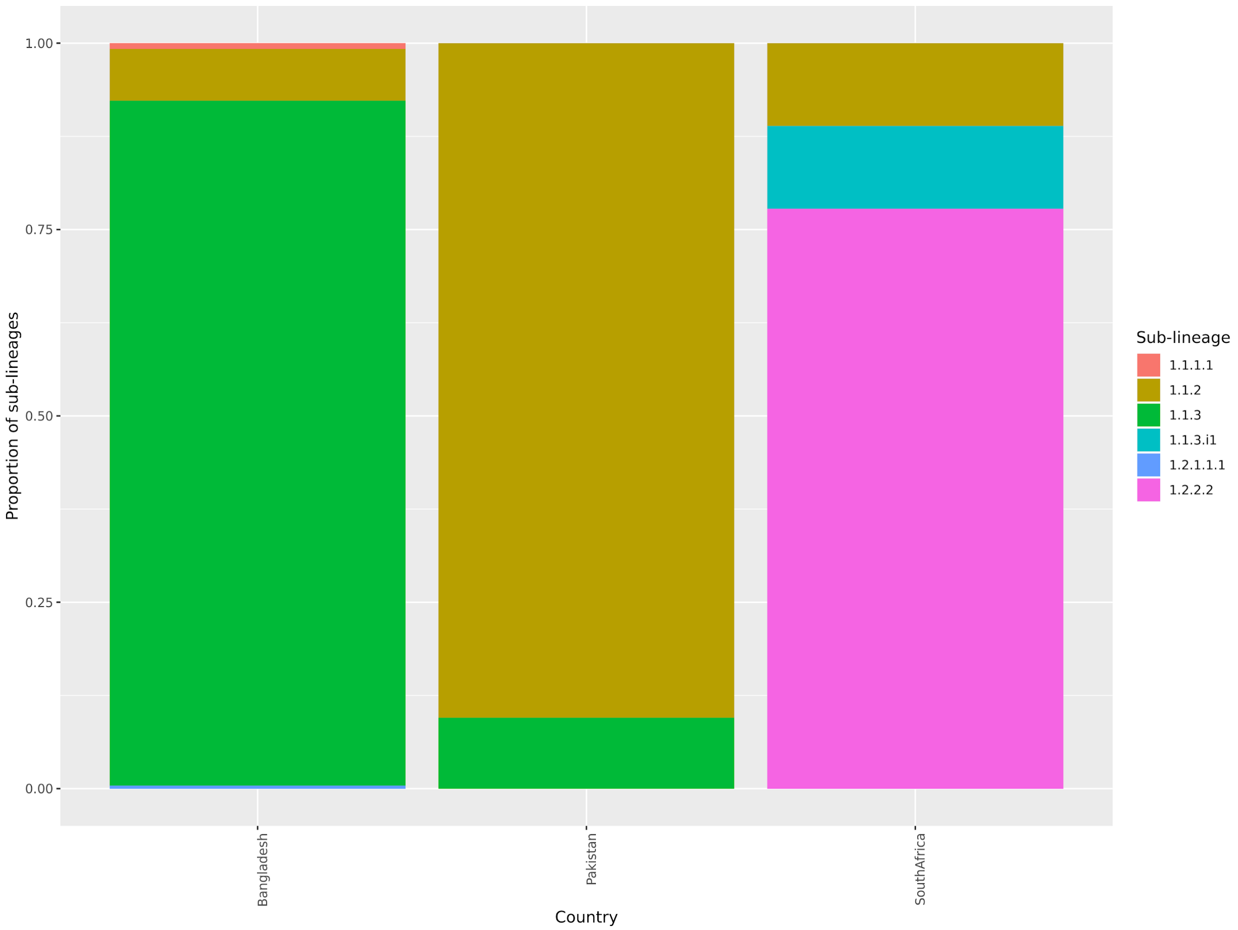

Suppl. Figure 16. Prevalence of different lineage 1 sub-lineages in three countries where isolates were randomly sampled. Colors indicate different sub-lineages. No lineage 1 isolates were found in Azerbaijan and Ukraine. Data: 259 isolates from Bangladesh, 21 from Pakistan and 9 from South Africa. Source data are provided as a Source Data file.

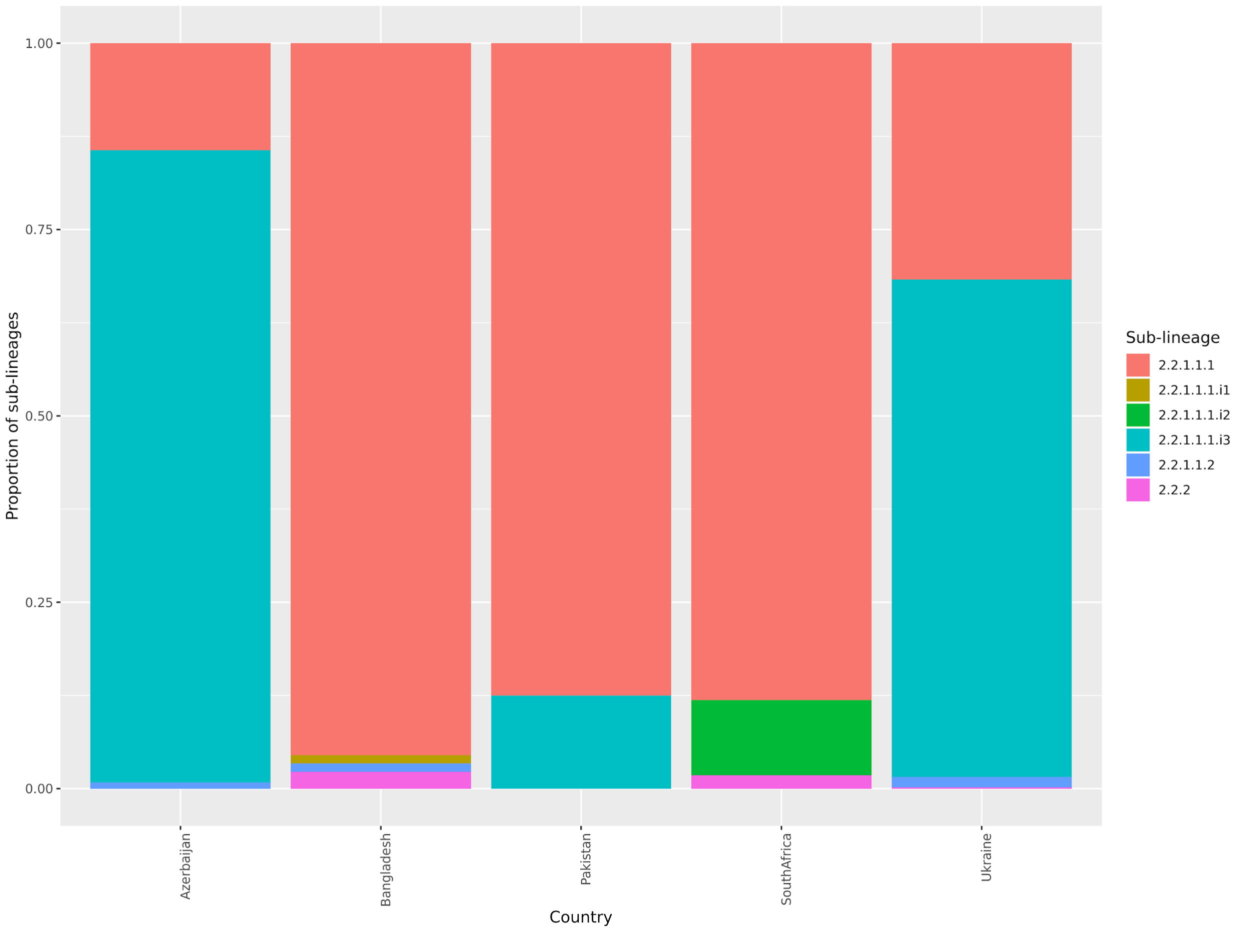

Suppl. Figure 17. Prevalence of different lineage 2 sub-lineages in five countries where isolates were randomly sampled. Colors indicate different sub-lineages. Data: 362 isolates from Azerbaijan, 89 from Bangladesh, 8 from Pakistan, 337 from South Africa and 517 from Ukraine. Source data are provided as a Source Data file.

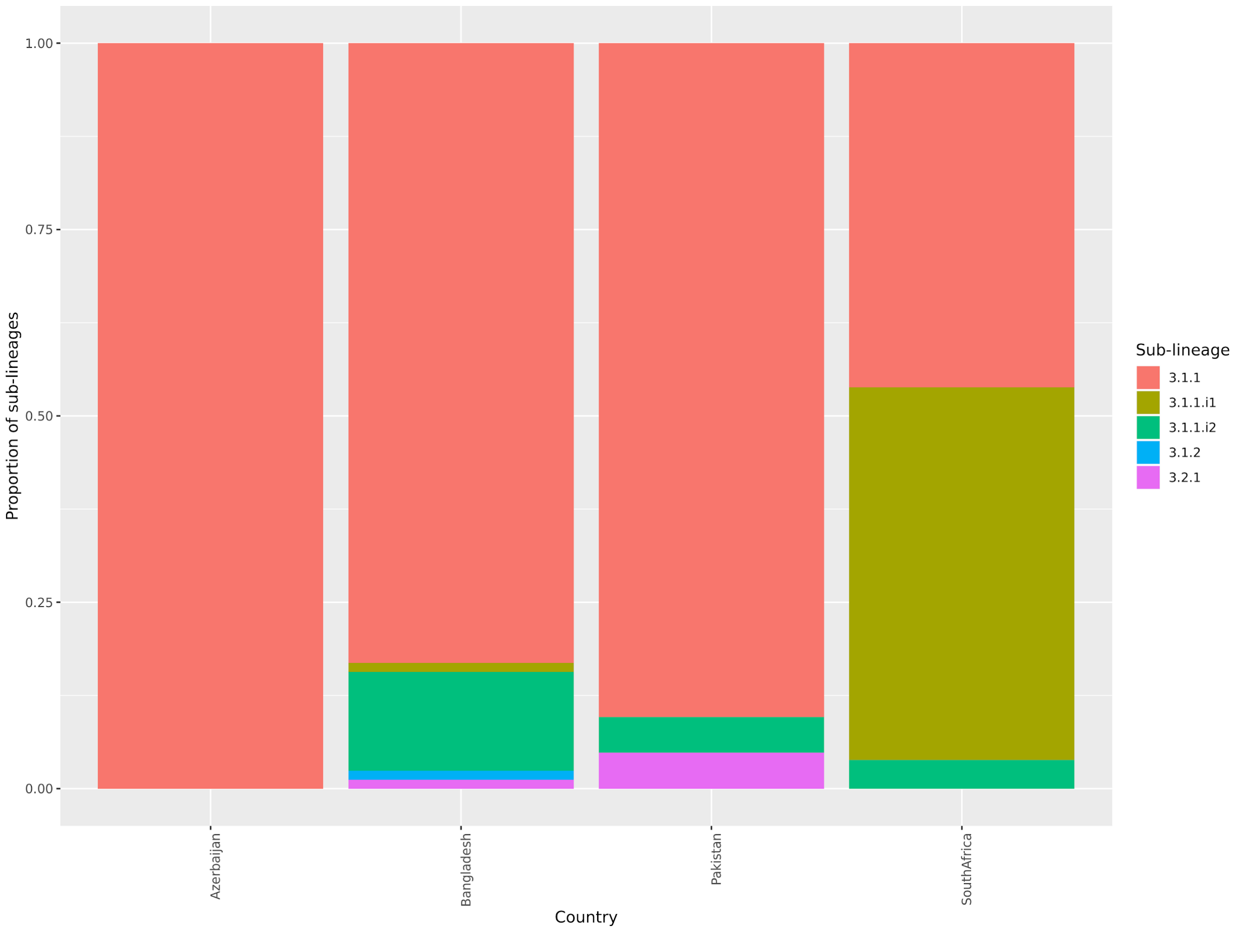

Suppl. Figure 18. Prevalence of different lineage 3 sub-lineages in four countries where isolates were randomly sampled. Colors indicate different sub-lineages. No lineage 3 isolates were found in Ukraine. Data: 2 isolates from Azerbaijan, 83 from Bangladesh, 125 from Pakistan, 52 from South Africa. Source data are provided as a Source Data file.

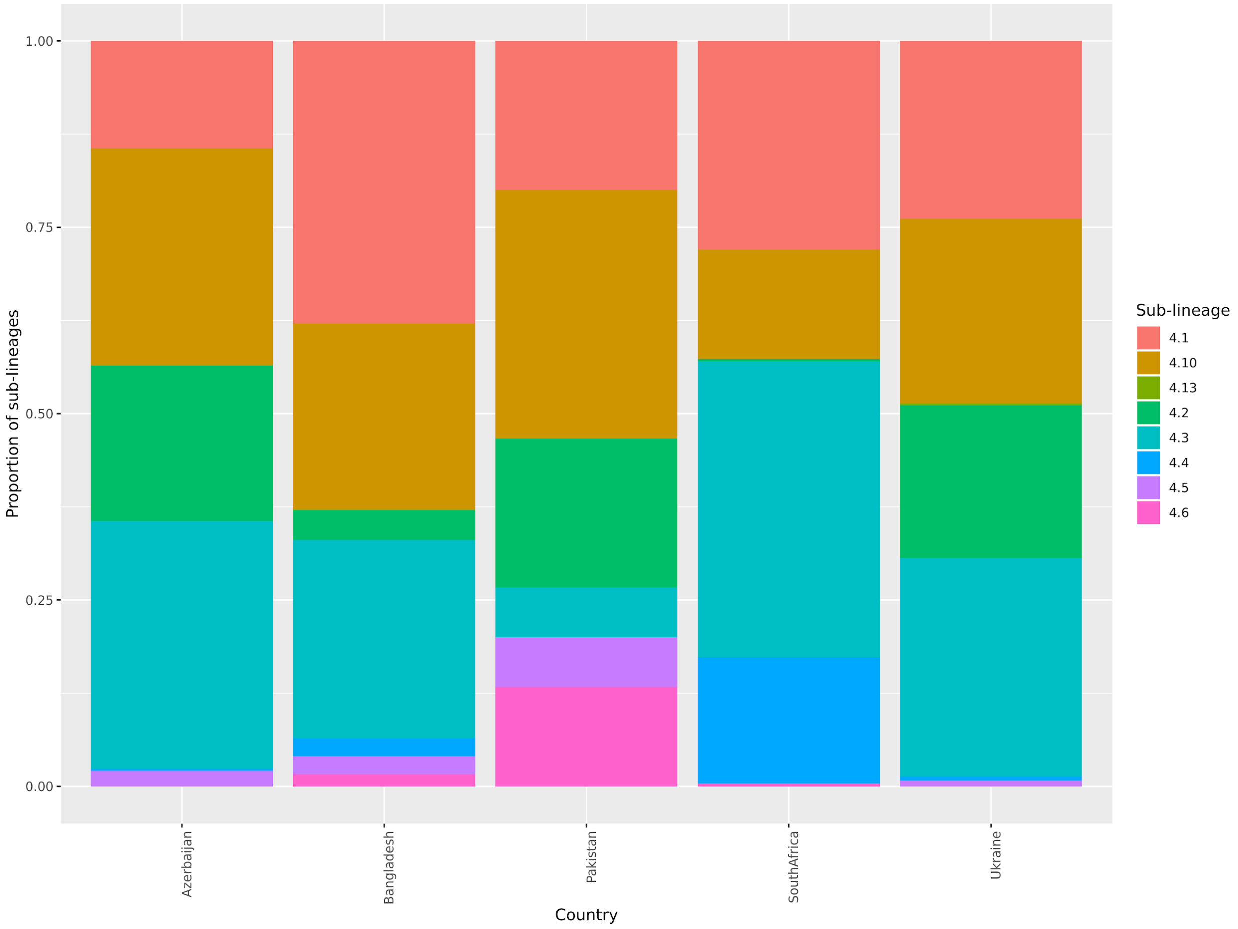

Suppl. Figure 19. Prevalence of different L4 sub-lineages in five countries where isolates were randomly sampled. Colors indicate different sub-lineages. Dataset: 340 isolates from Azerbaijan, 124 from Bangladesh, 15 from Pakistan, 808 from South Africa and 516 from Ukraine. Source data are provided as a Source Data file.

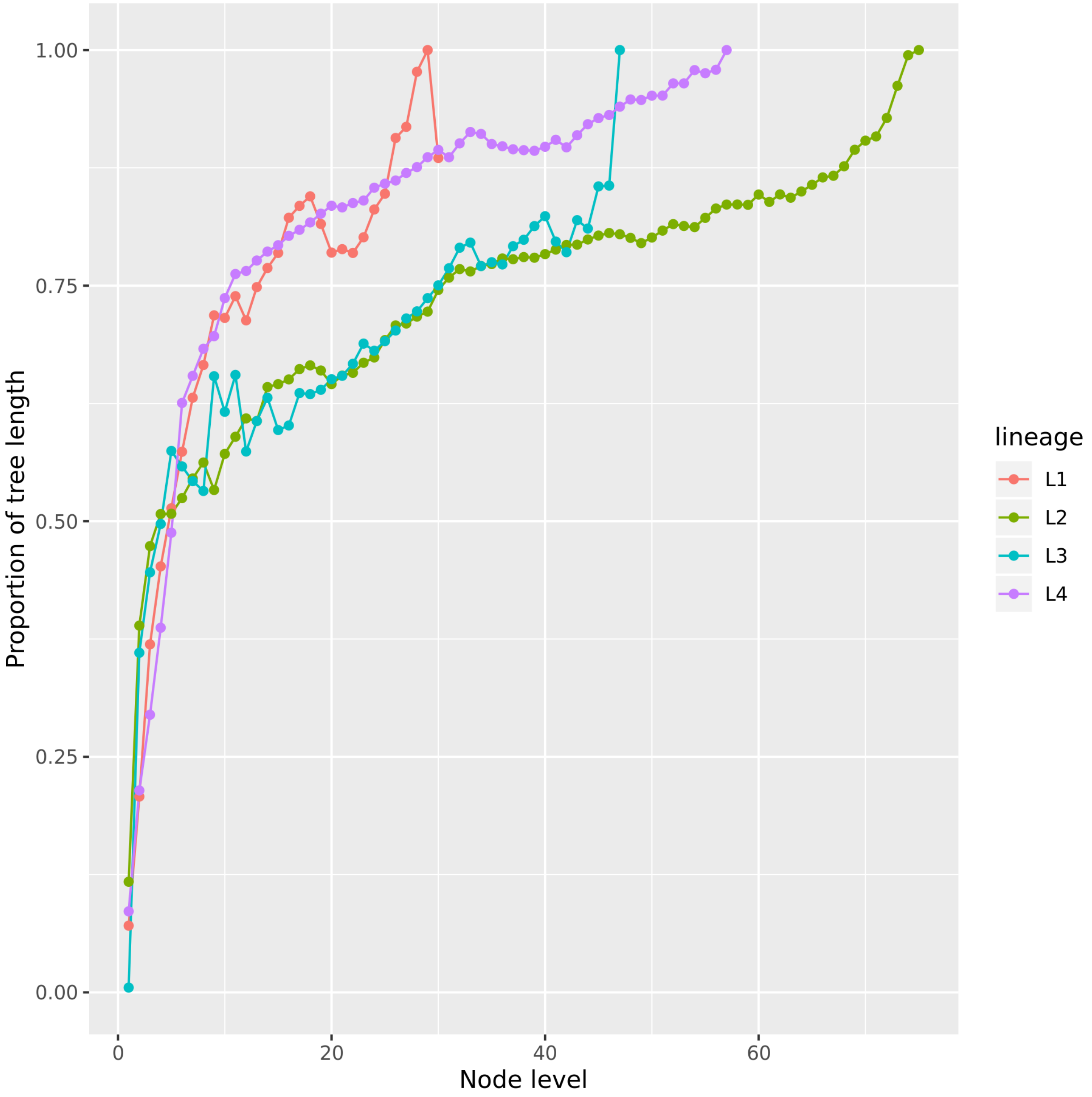

Suppl. Figure 20. Proportion of tree length as a function of node level. Colors identify the four Major *Mtb* lineages. Source data are provided as a Source Data file.

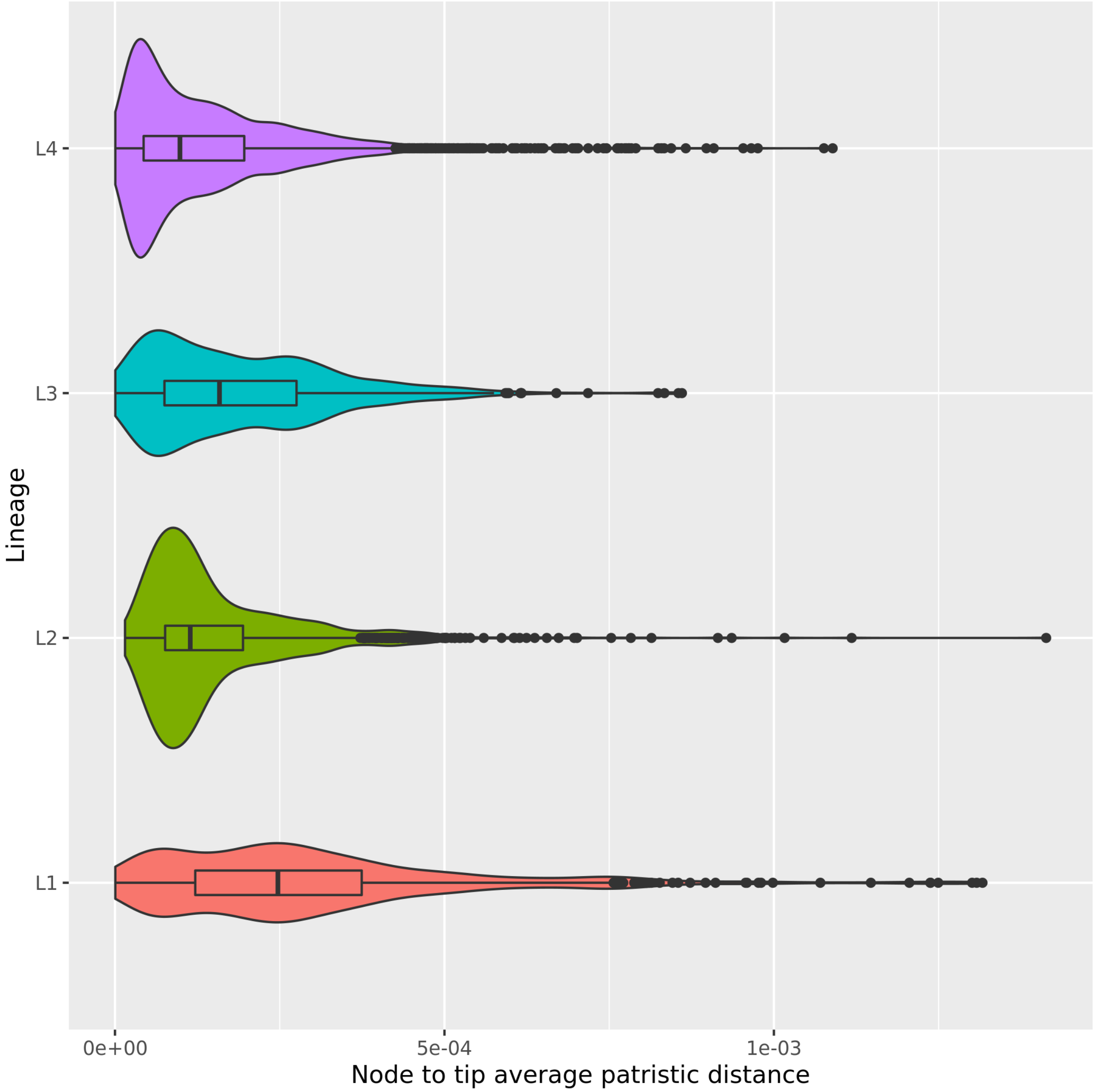

Suppl. Figure 21. Distribution of the node-to-tip distances for all internal nodes for all four *Mtb* lineages (L1-L4). Two sided Wilcoxon Rank Sum tests were performed to find if two distributions are significantly different. Medians: 9.85×10^{-5} (L4), 11.4×10^{-5} (L2), 15.8×10^{-5} (L3),

24.7×10^{-5} (L1). Comparisons: L1 vs L2 (p-value < 5.4×10^{-60}); L1 vs L3 (p-value < 7.3×10^{-23}); L1 vs L4 (p-value < 7.3×10^{-99}); L2 vs L3 (p-value < 4.7×10^{-9}); L2 vs L4 (p-value < 2.0×10^{-24}); L3 vs L4 (p-value < 1.6×10^{-37}). Description of the distributions (L1: n = 740, Min: 0.05×10^{-5}, 1st Quartile: 12.1×10^{-5}, Median: 24.7×10^{-5}, 3rd Quartile: 37.4×10^{-5}, Max: 131×10^{-5}; L2: n = 2194, Min: 1.52×10^{-5}, 1st Quartile: 7.6×10^{-5}, Median: 11.4×10^{-5}, 3rd Quartile: 19.4×10^{-5}, Max: 141×10^{-5}; L3: n = 1105, Min: 0.05×10^{-5}, 1st Quartile: 7.5×10^{-5}, Median: 15.8×10^{-5}, 3rd Quartile: 27×10^{-5}, Max: 86×10^{-5}; L4: n = 5524, Min: 0.05×10^{-5}, 1st Quartile: 4.3×10^{-5}, Median: 9.85×10^{-5}, 3rd Quartile: 19×10^{-5}, Max: 108×10^{-5}). Source data are provided as a Source Data file.

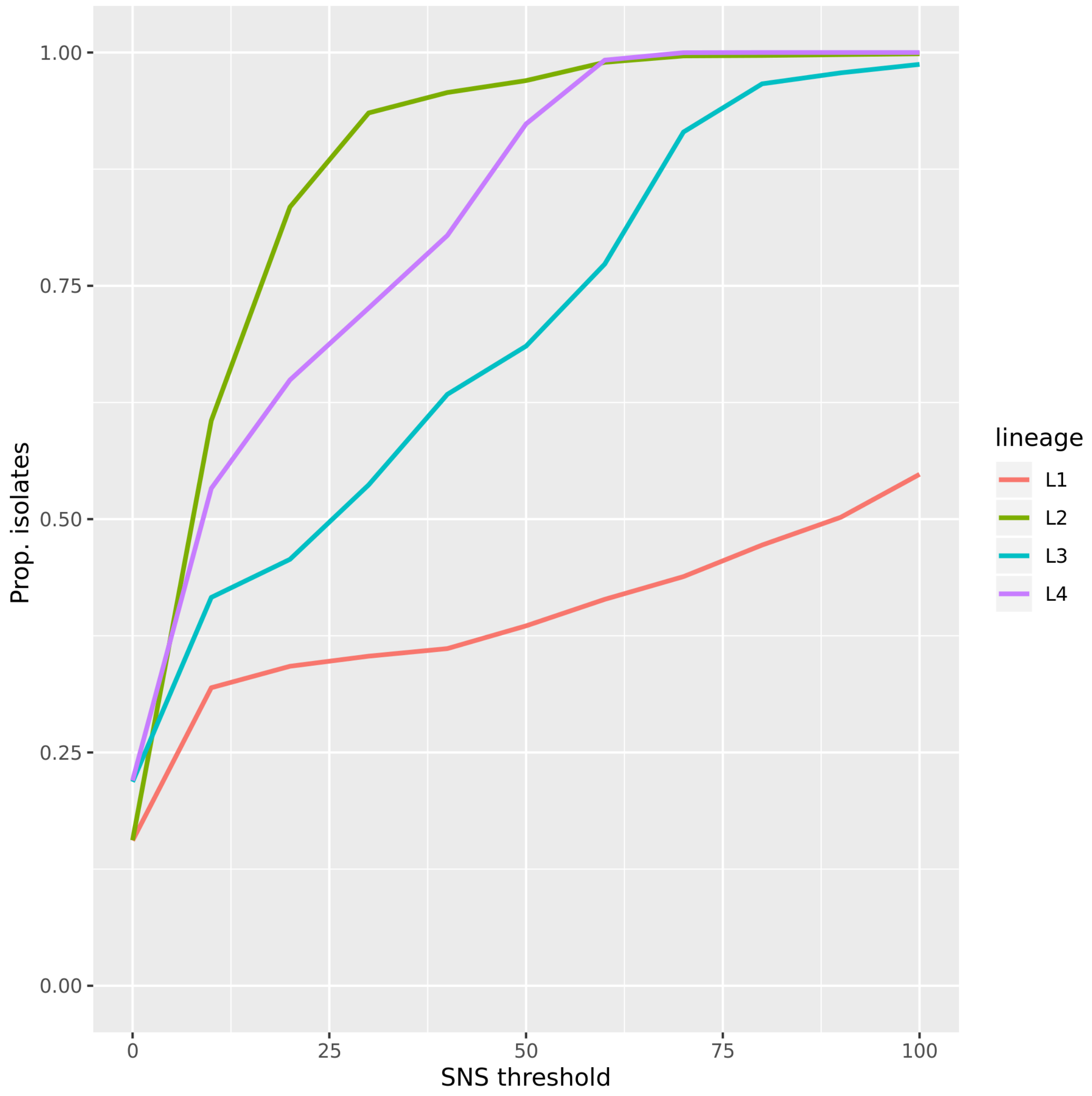

Suppl. Figure 22. Proportion of isolates from each one of the four major lineages (L1-4) that belong to clusters defined at different pairwise SNS difference thresholds. Colors identify the four Major *Mtb* lineages. Source data are provided as a Source Data file.

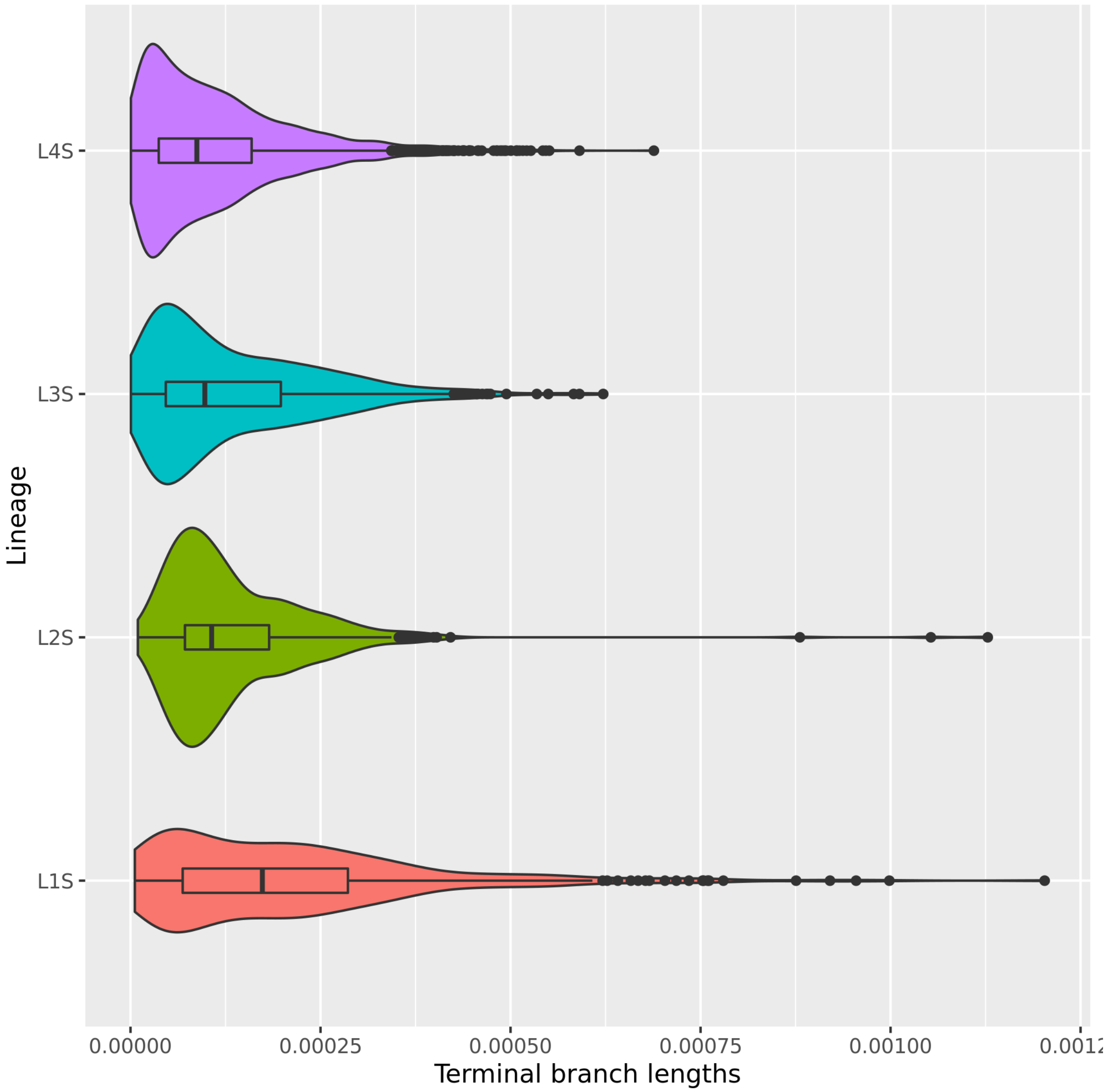

Suppl. Figure 23. Distributions of terminal branch lengths for the susceptible isolates belonging to the four global *Mtb* lineages (L1-L4) present in our curated dataset with resistance phenotypes. Two sided Wilcoxon Rank Sum tests were performed to test that two distributions were significantly different. Medians: 8.7×10^{-5} (L4S, n = 2951), 9.8×10^{-5} (L3S, n = 859), 10.6×10^{-5} (L2S, n = 564), 17×10^{-5} (L1S, n = 564). Comparisons: L1S vs L2S (4.9×10^{-9}); L1S vs L3S (1.1×10^{-14}); L1S vs L4S (5.4×10^{-39}); L2S vs L3S (0.05); L2S vs L4S (1.8×10^{-12}); L3S vs L4S (3.2×10^{-6}). Description of the distributions (L1S: n = 564, Min: 0.57×10^{-5},

1st Quartile: 6.8×10^{-5}, Median: 17×10^{-5}, 3rd Quartile: 28.6×10^{-5}, Max: 120×10^{-5}; L2S: n = 564, Min: 0.96×10^{-5}, 1st Quartile: 7.15×10^{-5}, Median: 10.6×10^{-5}, 3rd Quartile: 18.2×10^{-5}, Max: 112×10^{-5}; L3S: n = 859, Min: 0.05×10^{-5}, 1st Quartile: 4.6×10^{-5}, Median: 9.8×10^{-5}, 3rd Quartile: 19.7×10^{-5}, Max: 62×10^{-5}; L4S: n = 2951, Min: 0.05×10^{-5}, 1st Quartile: 3.7×10^{-5}, Median: 8.7×10^{-5}, 3rd Quartile: 15.9×10^{-5}, Max: 68.8×10^{-5}). Source data are provided as a Source Data file.

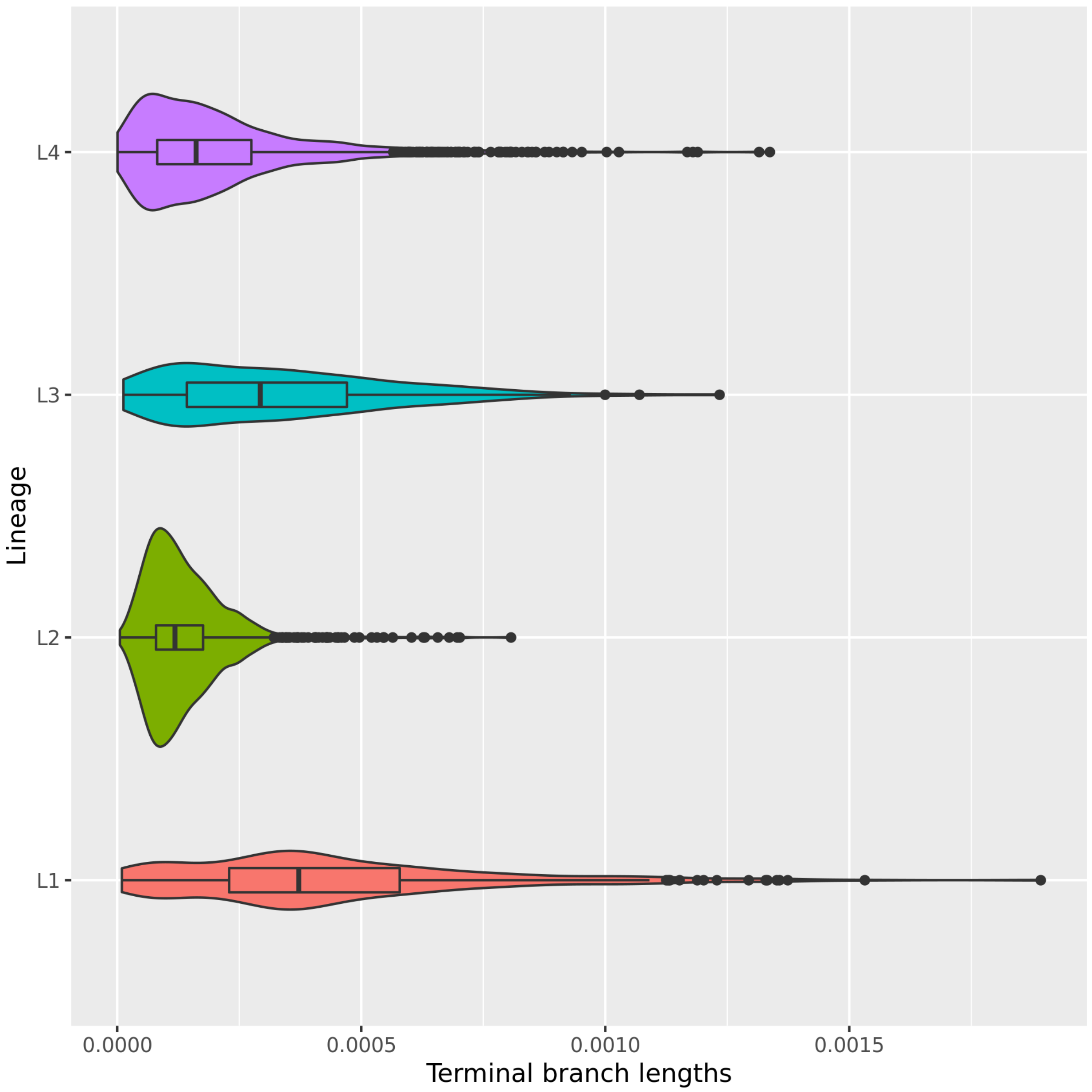

Suppl. Figure 24. Distributions of terminal branch lengths for the isolates belonging to the four global *Mtb* lineages (L1-L4) present in the WHO / Zignol *et al.* dataset where isolates were systematically collected in five countries [3]. Two sided Wilcoxon Rank Sum tests were performed to test that two distributions were significantly different. Medians: 11.8×10^{-5} (L2, n = 1313), 16.1×10^{-5} (L4, n = 1837), 29.3×10^{-5} (L3, n = 264), 37.2×10^{-5} (L1, n = 377). Comparisons: L1 vs L2 (1.3×10^{-83}); L1 vs L3 (1.4×10^{-4}); L1 vs L4 (2.9×10^{-51}); L2 vs L3 (7.0×10^{-43}); L2 vs L4 (1.3×10^{-22}); L3 vs L4 (1.1×10^{-17}). Description of the distributions (L1: n = 377, Min: 0.95×10^{-5}, 1st Quartile: 22×10^{-5}, Median: 37.2×10^{-5}, 3rd Quartile: 57.8×10^{-5}, Max: 189×10^{-5}; L2: n =

1313, Min: 0.5 × 10^{-5}, 1st Quartile: 7.9 × 10^{-5}, Median: 11.8 × 10^{-5}, 3rd Quartile: 17.5 × 10^{-5}, Max: 80.6 × 10^{-5}; L3: n = 264, Min: 1.2 × 10^{-5}, 1st Quartile: 14.2 × 10^{-5}, Median: 29.3 × 10^{-5}, 3rd Quartile: 47.0 × 10^{-5}, Max: 123 × 10^{-5}; L4: n = 1837, Min: 0.04 × 10^{-5}, 1st Quartile: 8.1 × 10^{-5}, Median: 16.1 × 10^{-5}, 3rd Quartile: 27.4 × 10^{-5}, Max: 133 × 10^{-5}). Source data are provided as a Source Data file.

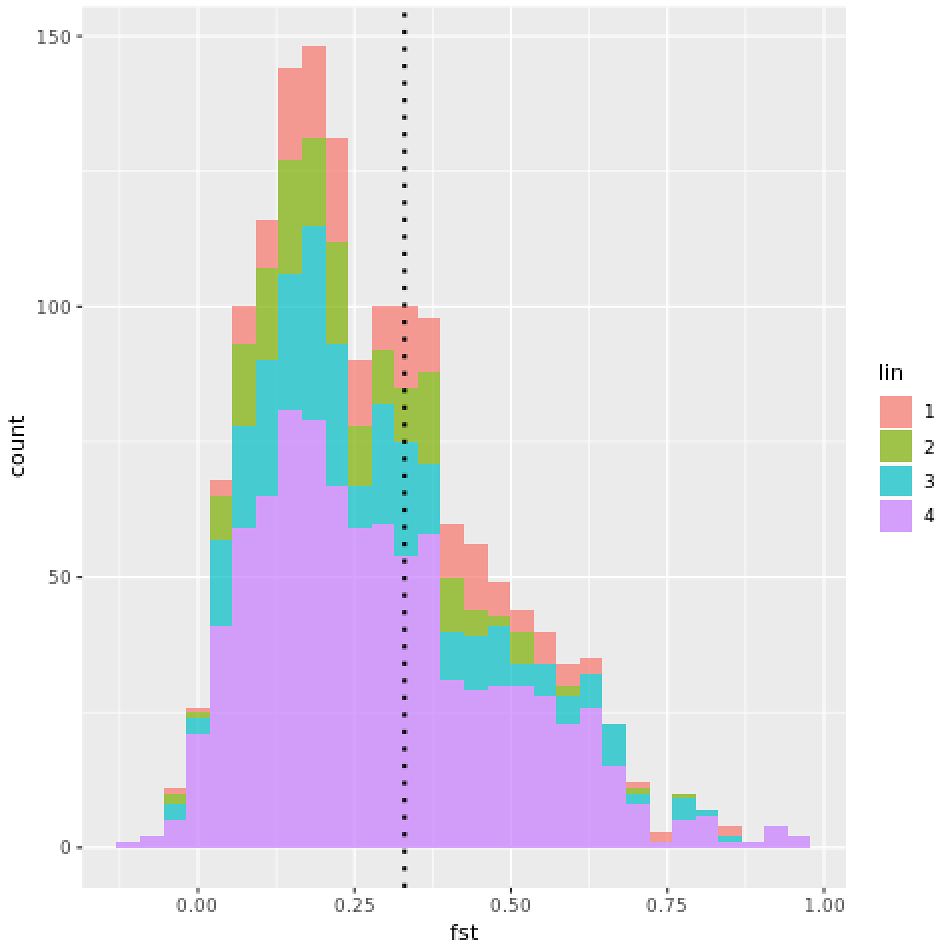

Suppl. Figure 25. Distribution of F_{ST} (fixation index) values calculated on all internal nodes of each of the phylogenetic trees of lineages 1-4 (pan-susceptible isolates). The dotted line shows the threshold we chose to define two sub-lineages as distinct ones (0.33). Source data are provided as a Source Data file.

Supplementary Tables

Sub-lineage	Simpson diversity index
4.6.2/Cameroon	0.278
4.6.1/Uganda	0.280
4.5	0.399
4.1.3/Ghana	0.496

Suppl. Table 1. Lineage 4 Simpson diversity index for the geographically restricted sub-lineages described by Stucki *et al.* [4].

Sub-lineage	Simpson diversity index
4.10/PGG3	0.525
4.3/LAM	0.553
4.1.2/Haarlem	0.608

Suppl. Table 2. Lineage 4 Simpson diversity index for the geographically unrestricted sub-lineages described by Stucki *et al.* [4]

Sub-lineage	Simpson diversity index	Num. of isolates	Num. of countries	Num. of continents	Notes
2.2.1.1.1.i2	0.0484682	81	4	1	Outbreak?
1.1.3.i1 / Malawi	0.0560281	104	2	2	
4.2.1.1	0.0884438	239	14	3	
4.11	0.1138659	33	3	2	

1.1.1.1	0.1239076	353	7	3	
2.1	0.1874700	79	10	3	
2.2.1.1.2	0.1988040	101	12	3	
3.1.2	0.2390123	45	5	3	
4.3.i2	0.2512359	818	18	3	
4.6.1.1.1.1.2	0.2539541	67	7	2	4.6.1/Uganda
2.2.1.1.1.i3	0.2765438	837	15	3	Central Asia

Suppl. Table 3. Summary table of the geographic distribution of sub-lineages / internal groups having Simpson diversity index < 0.28 (which corresponds to the lowest Simpson diversity index value for known geographically restricted sub-lineages)

Sub-lineage	Simpson diversity index	Num. of isolates	Num. of countries	Num. of continents	Notes
4.10.i2	0.7152186	239	21	5	Corresp. to 4.9 / Internal group of 4.10/PGG3
4.3.i3.1	0.6925104	595	31	5	Internal group of 4.3/LAM
4.3.i4.1	0.6870217	195	19	5	Internal group of 4.3/LAM
4.1.i1.1.1.1	0.6712246	1345	33	5	Internal group of 4.1/ corresp to 4.1.2/Haarlem
2.2.1.1.1	0.6668162	3289	24	5	~ modern Beijing
4.1.i1.2.1	0.6505840	751	24	4	
2.2.2	0.6241035	242	14	4	
1.2.1.1.2	0.6234568	36	6	3	
4.2.2	0.6207767	227	20	4	
1.1.2	0.6140699	253	7	4	
4.4.1.1	0.6053944	586	23	4	Corresp. to 4.4.1.1

Suppl. Table 4. Summary table of the geographic distribution of sub-lineages / internal groups having Simpson diversity index > 0.6 (which corresponds to the highest Simpson diversity index value for known geographically unrestricted sub-lineages)

Supplementary References

1. Coll, F. *et al.* A robust SNP barcode for typing Mycobacterium tuberculosis complex strains. *Nat. Commun.* **5**, 4812 (2014).
2. Shitikov, E. *et al.* Evolutionary pathway analysis and unified classification of East Asian lineage of Mycobacterium tuberculosis. *Sci. Rep.* **7**, 9227 (2017).
3. Zignol, M. *et al.* Genetic sequencing for surveillance of drug resistance in tuberculosis in highly endemic countries: a multi-country population-based surveillance study. *Lancet Infect. Dis.* **18**, 675–683 (2018).
4. Stucki, D. *et al.* Mycobacterium tuberculosis lineage 4 comprises globally distributed and geographically restricted sublineages. *Nat. Genet.* **48**, 1535–1543 (2016).
