# Supplementary material for: Population structure, biogeography and transmissibility of Mycobacterium tuberculosis
Source: Nat Commun. 2021 Oct 20;12:6099. doi: 10.1038/s41467-021-26248-1 (PMC8528816; doi:10.1038/s41467-021-26248-1)
Supplement: Supplementary file 3 — Description of Additional Supplementary Files [file 41467_2021_26248_MOESM3_ESM.pdf]

Description of Additional Supplementary Files

File Name: Supplementary Data 1
Description: Accession numbers, lineage designations, country of origin for the dataset of isolates with drug resistance phenotypic data.

File Name: Supplementary Data 2
Description: Sub-lineage designation support (Fst and bootstrap) and comparison with previous SNS schemes.

File Name: Supplementary Data 3
Description: SNS scheme to perform lineage calling with the the sub-lineage designations described in this work.

File Name: Supplementary Data 4
Description: Accession number, lineage designations, country of origin for the isolates belonging to the NCBI dataset used to determine the geographic distribution of Mtb sub-lineages.

File Name: Supplementary Data 5
Description: Geographic distribution of each *Mycobacterium tuberculosis* sublineage.

File Name: Supplementary Data 6
Description: Accession number, lineage designations, country of origin for the Zignol et al. dataset.

File Name: Supplementary Data 7
Description: Number of isolates per sub-lineage or country for each of the three datasets.

File Name: Supplementary Data 8
Description: Genomic coordinates excluded.
