# Supplementary material for: Population structure, biogeography and transmissibility of Mycobacterium tuberculosis
Source: Nat Commun. 2021 Oct 20;12:6099. doi: 10.1038/s41467-021-26248-1 (PMC8528816; doi:10.1038/s41467-021-26248-1)

Distribution of sub−lineage 1.1.1.1

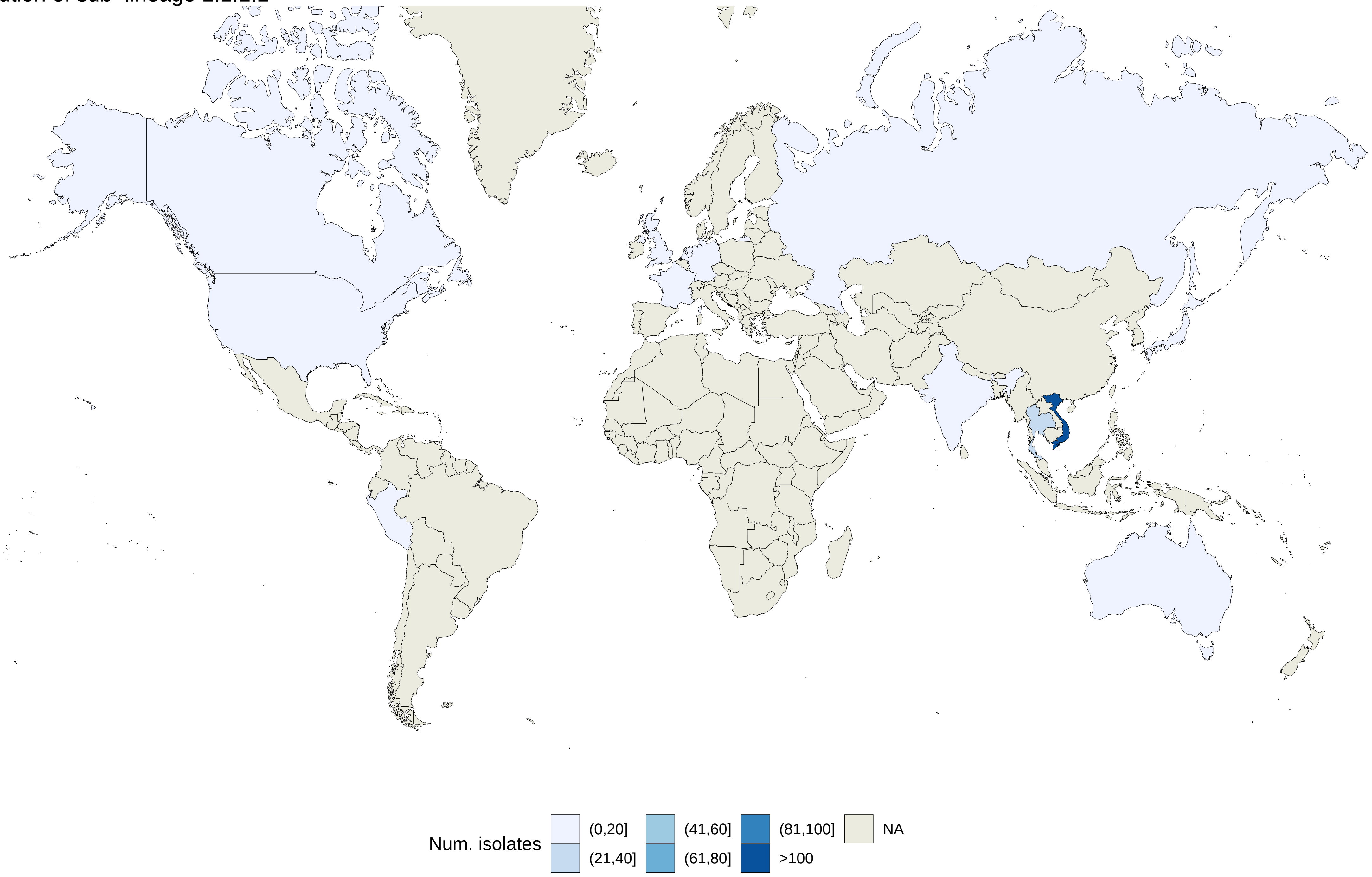

Distribution of sub−lineage 1.1.1.2

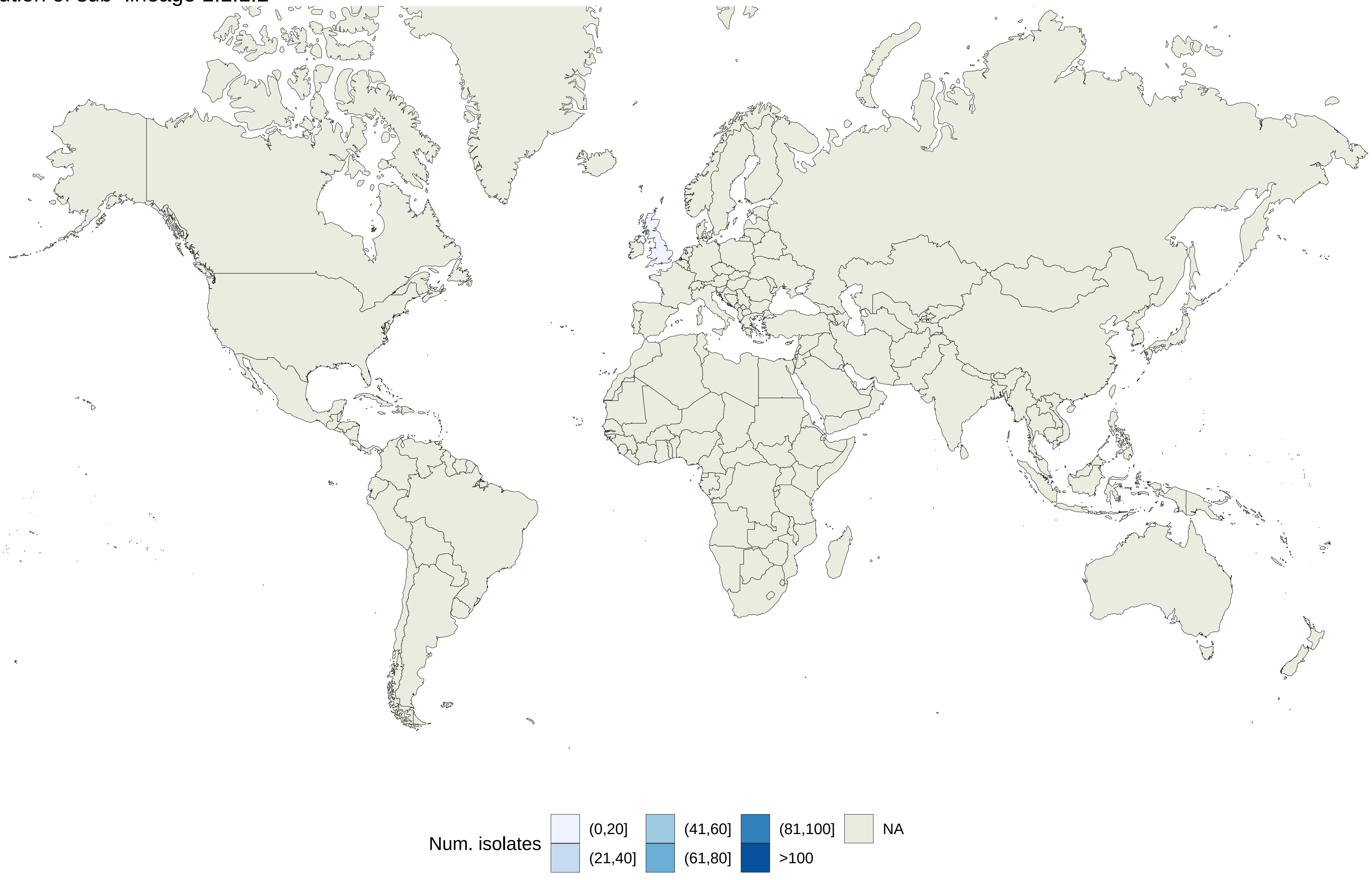

Distribution of sub−lineage 1.1.2

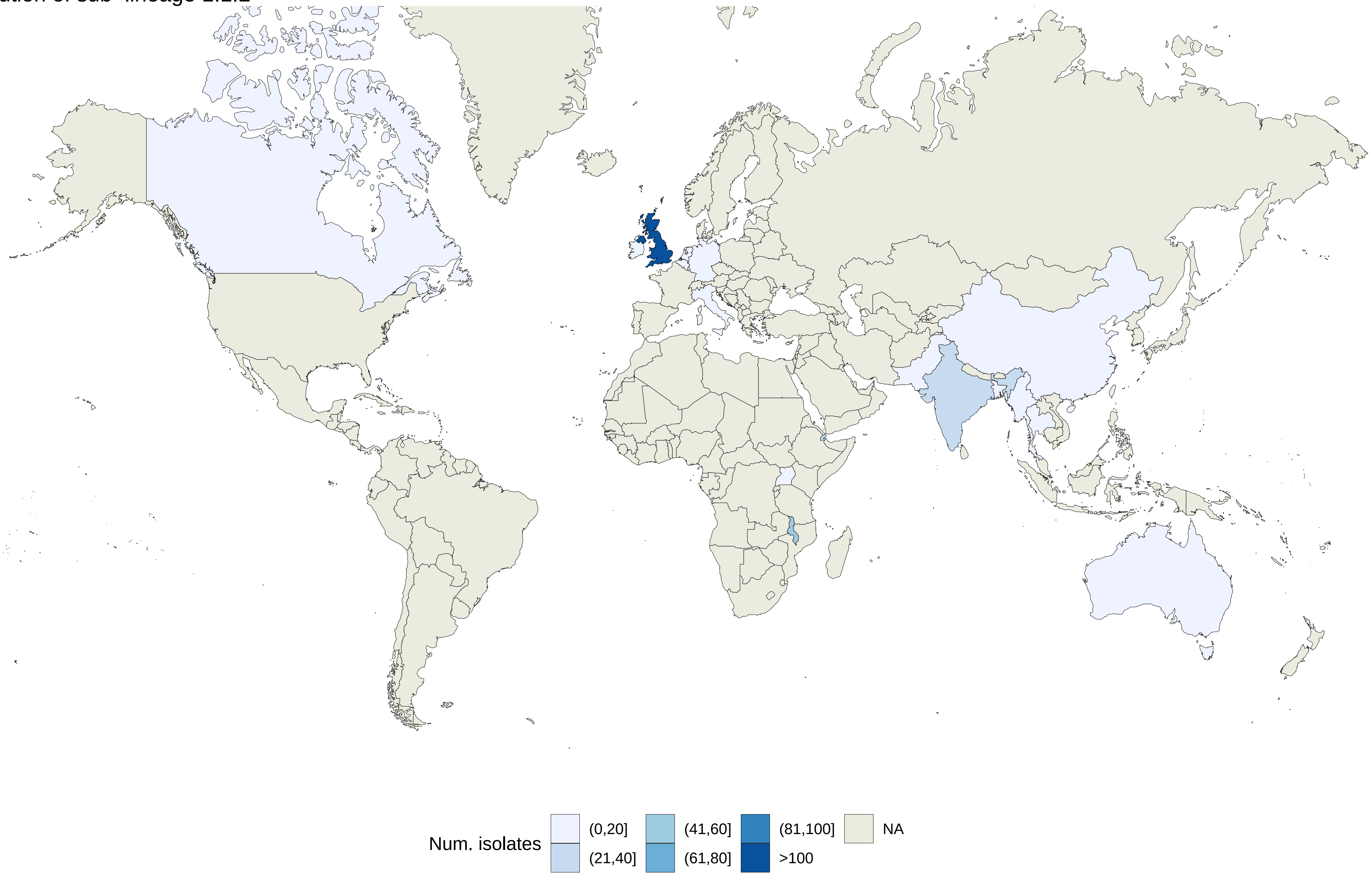

Distribution of sub−lineage 1.1.3.i1

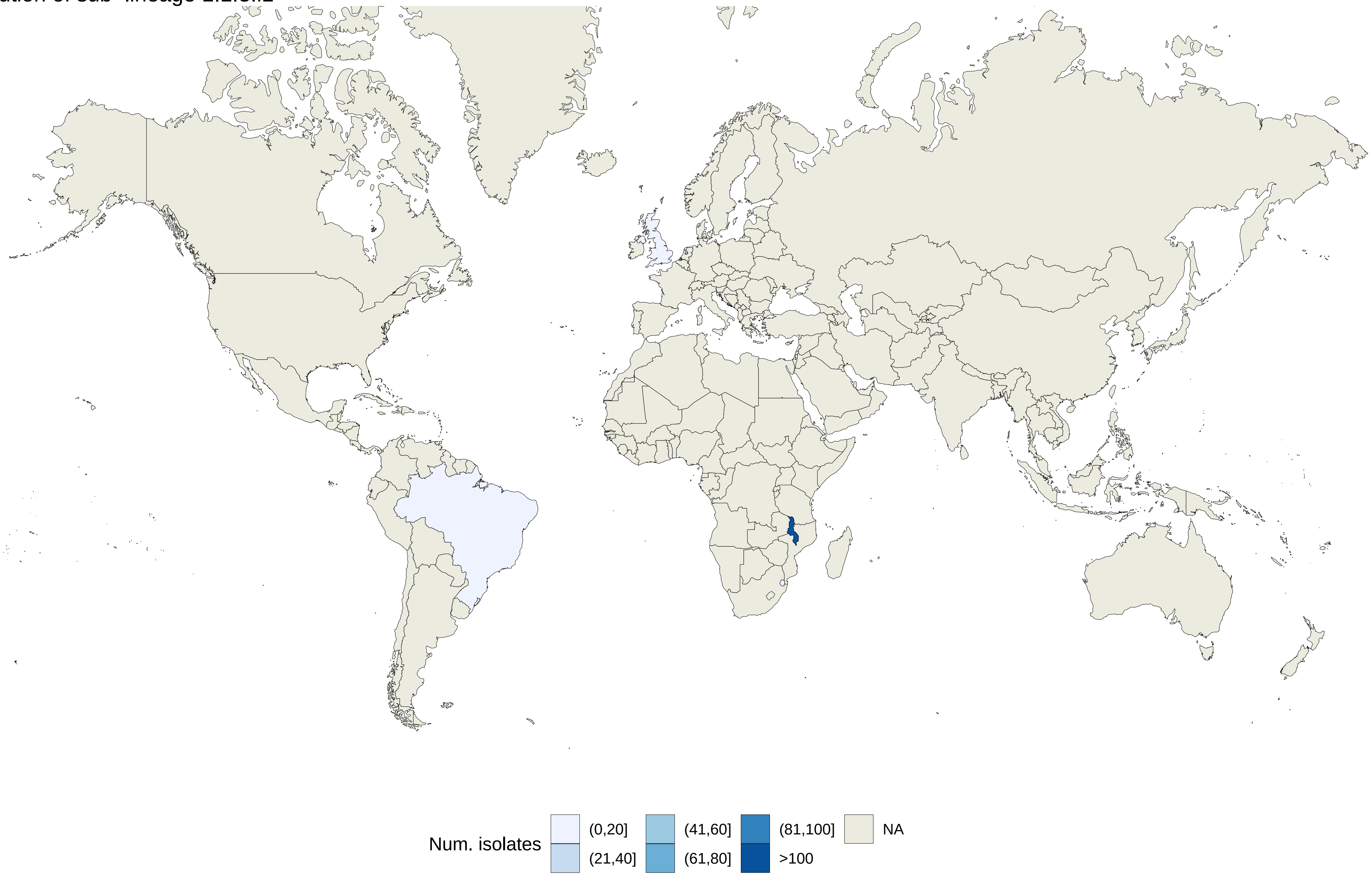

Distribution of sub−lineage 1.1.3

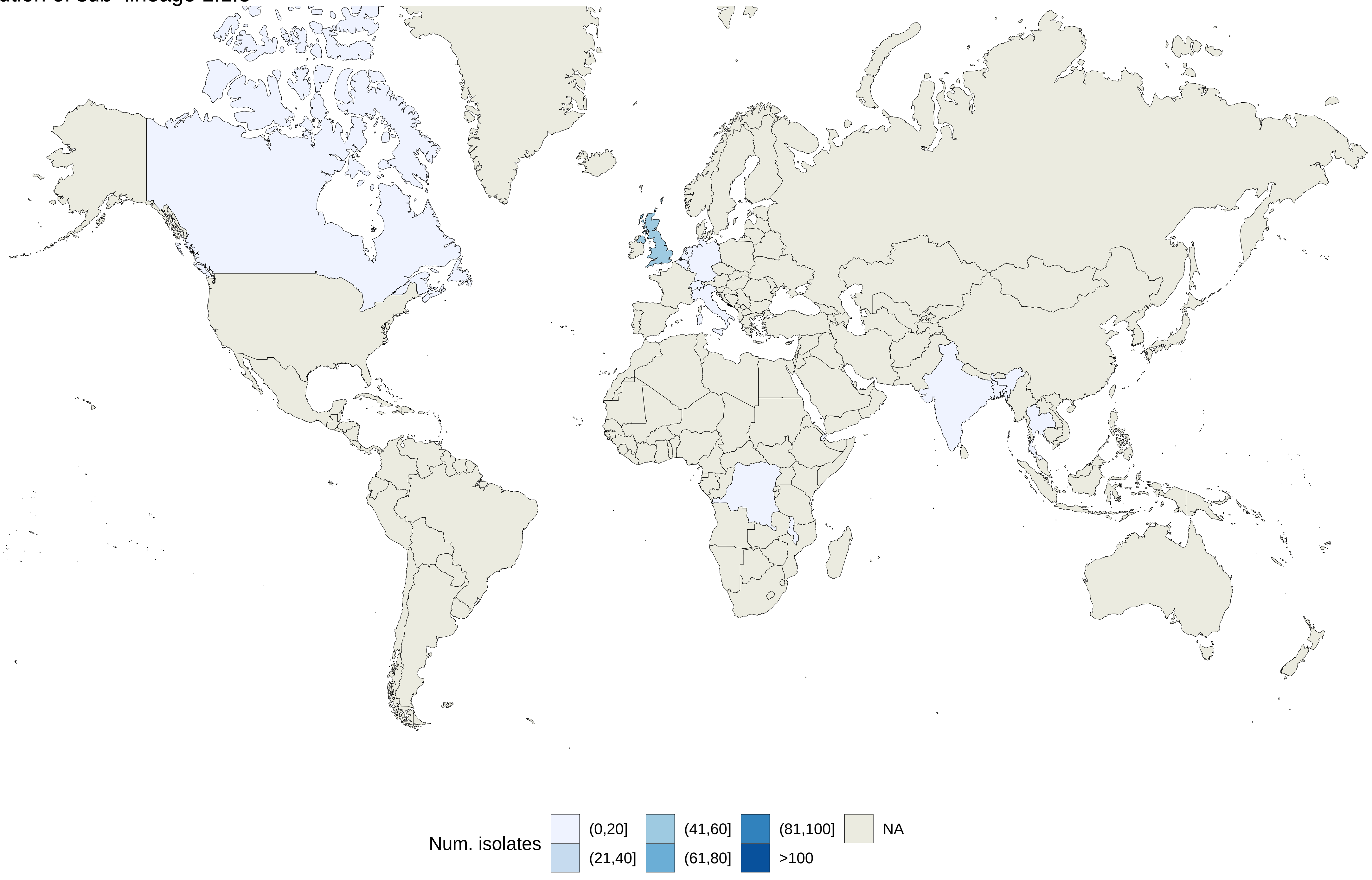

Distribution of sub−lineage 1.2.1.1.1

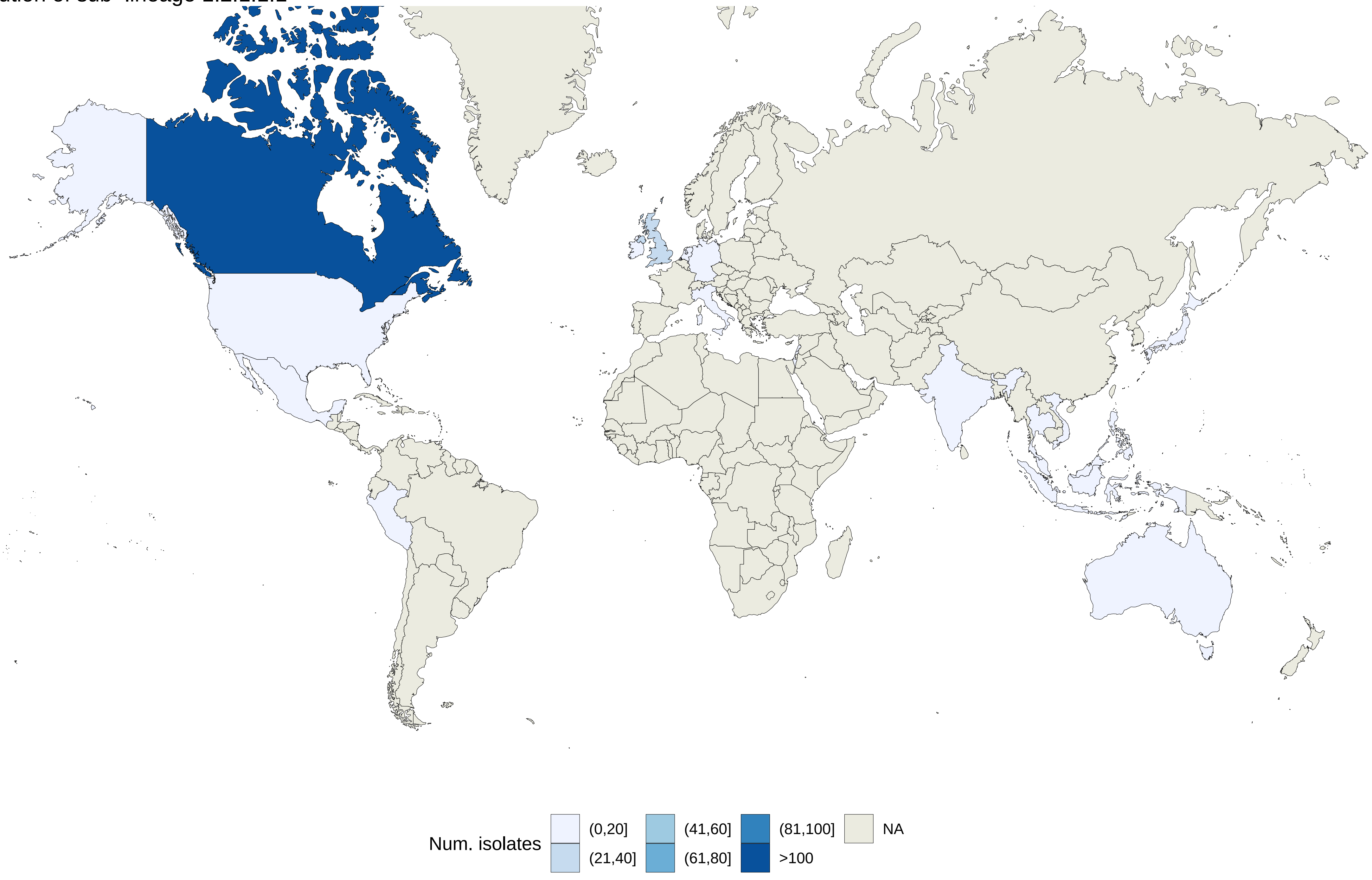

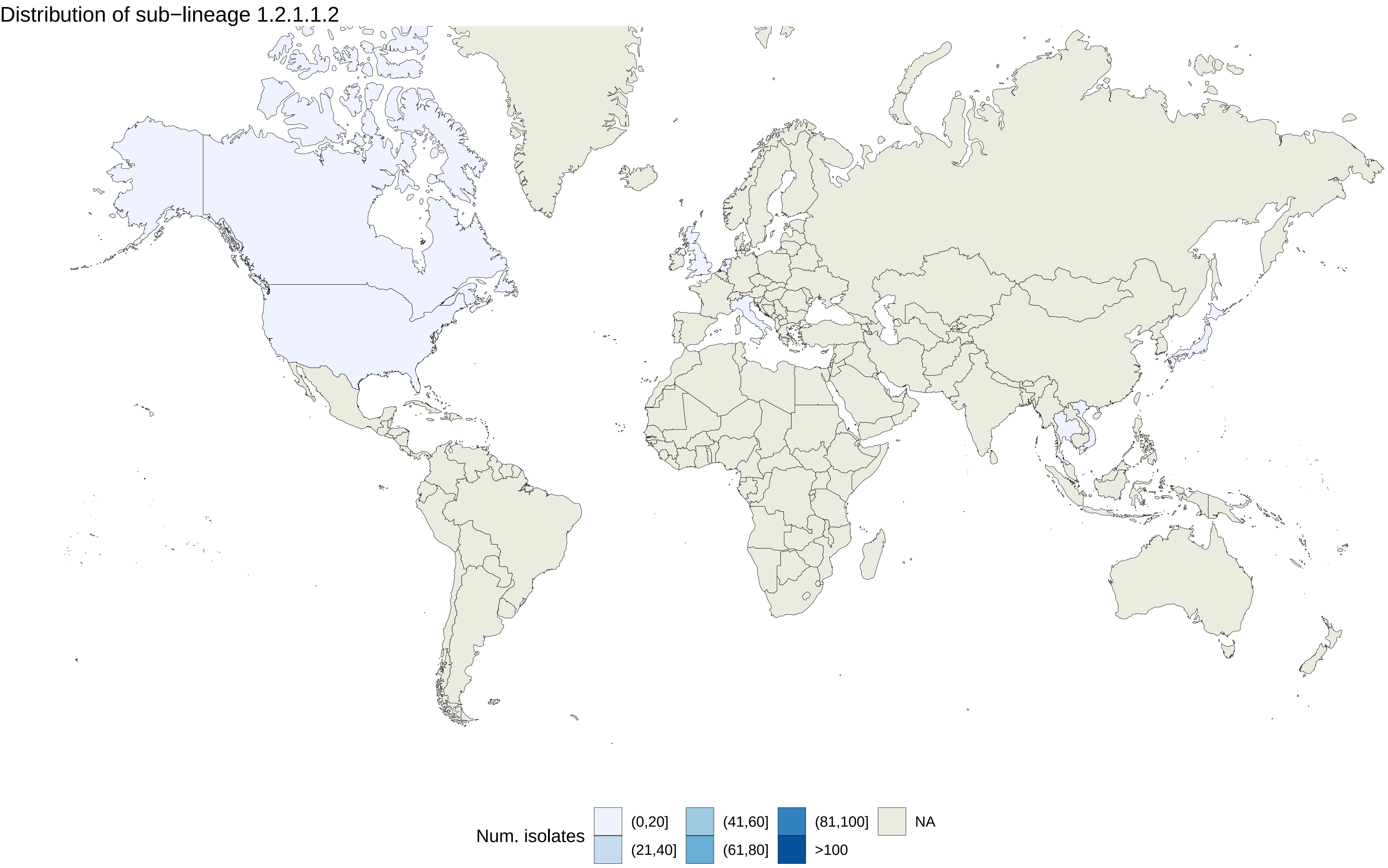
Distribution of sub−lineage 1.2.1.1.2
Num. isolates
(0,20]
(21,40]
(41,60]
(61,80]
(81,100]
>100
NA

Distribution of sub−lineage 1.2.1.2

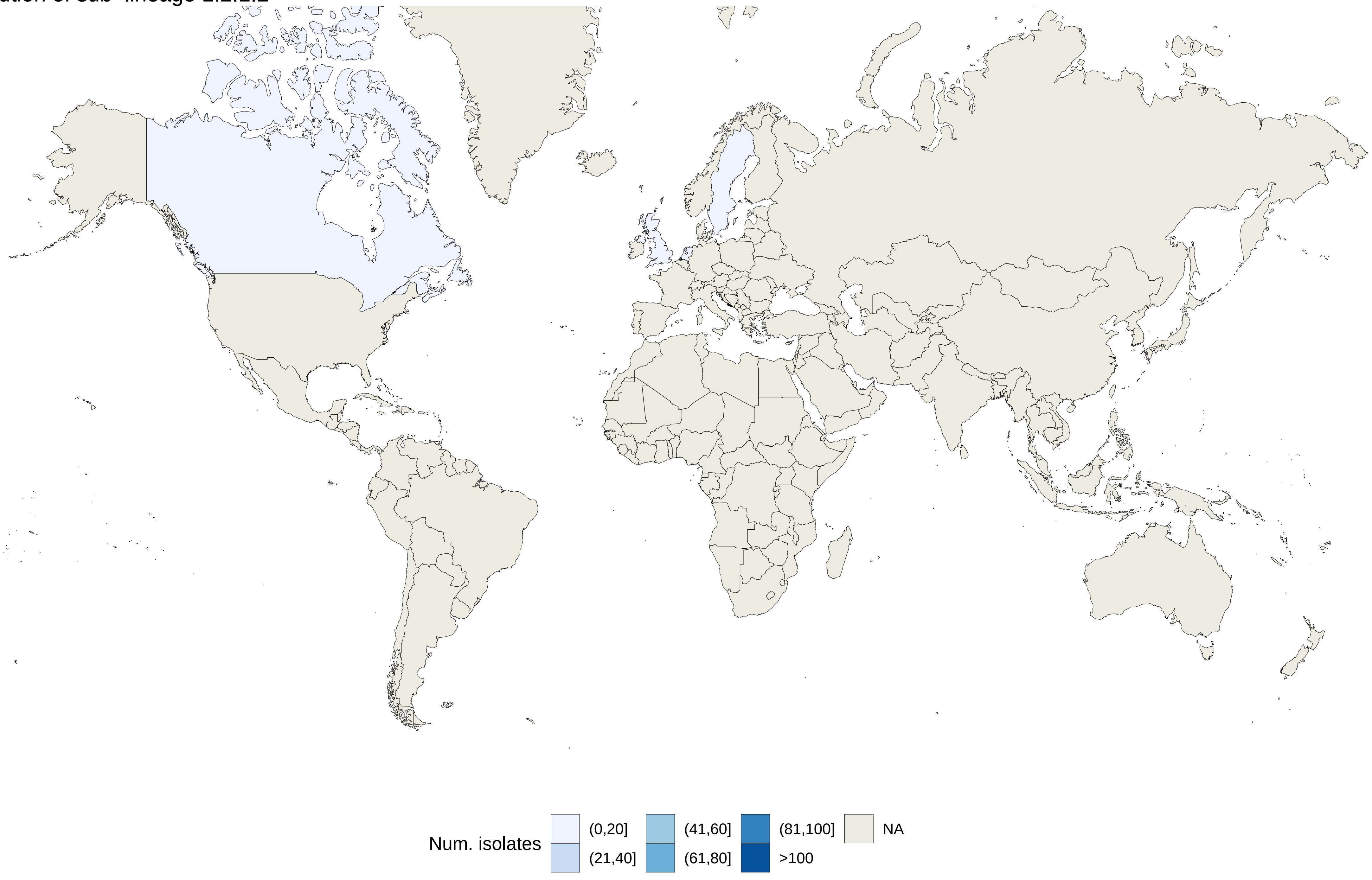

Distribution of sub−lineage 1.2.2.1

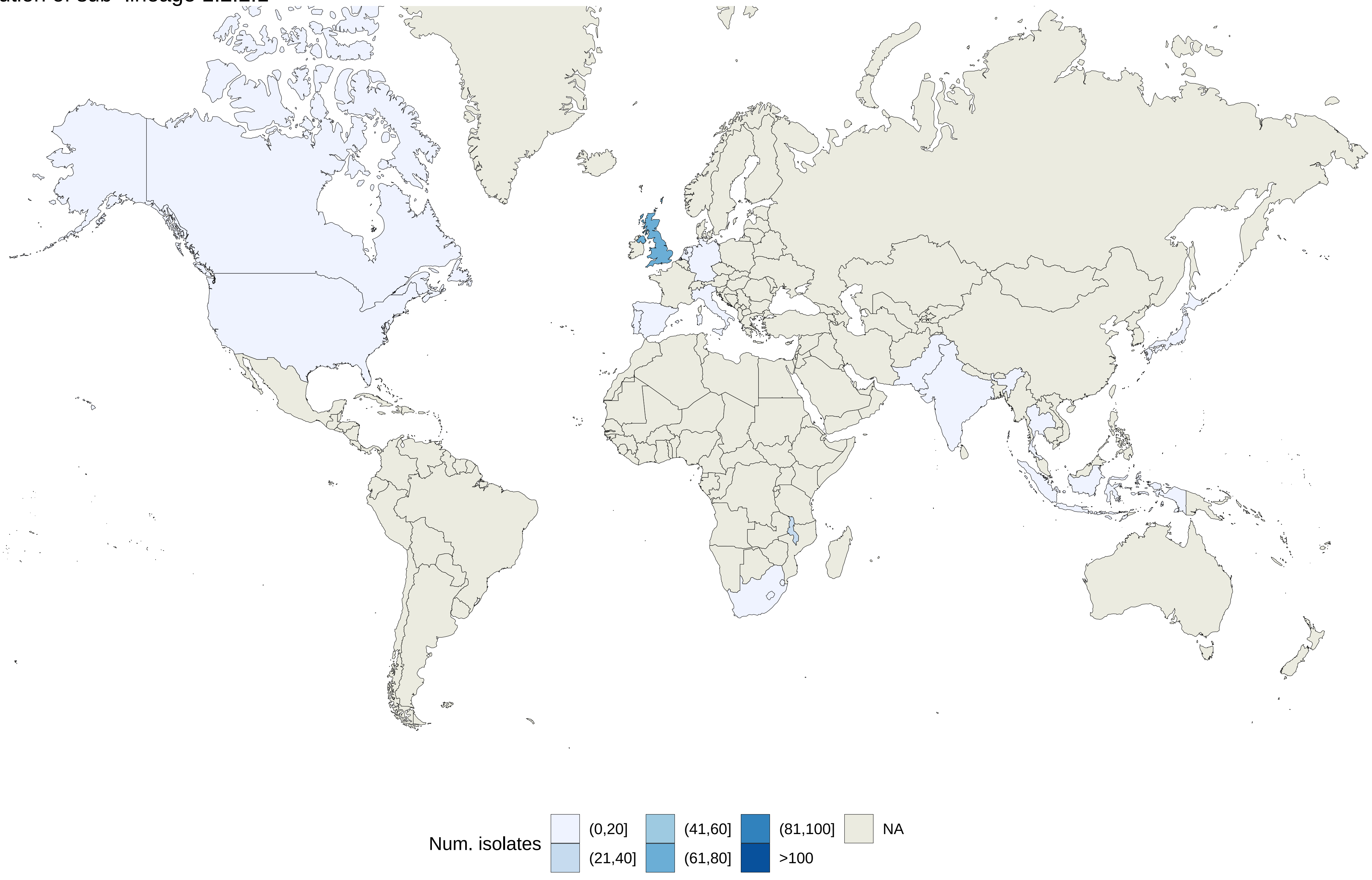

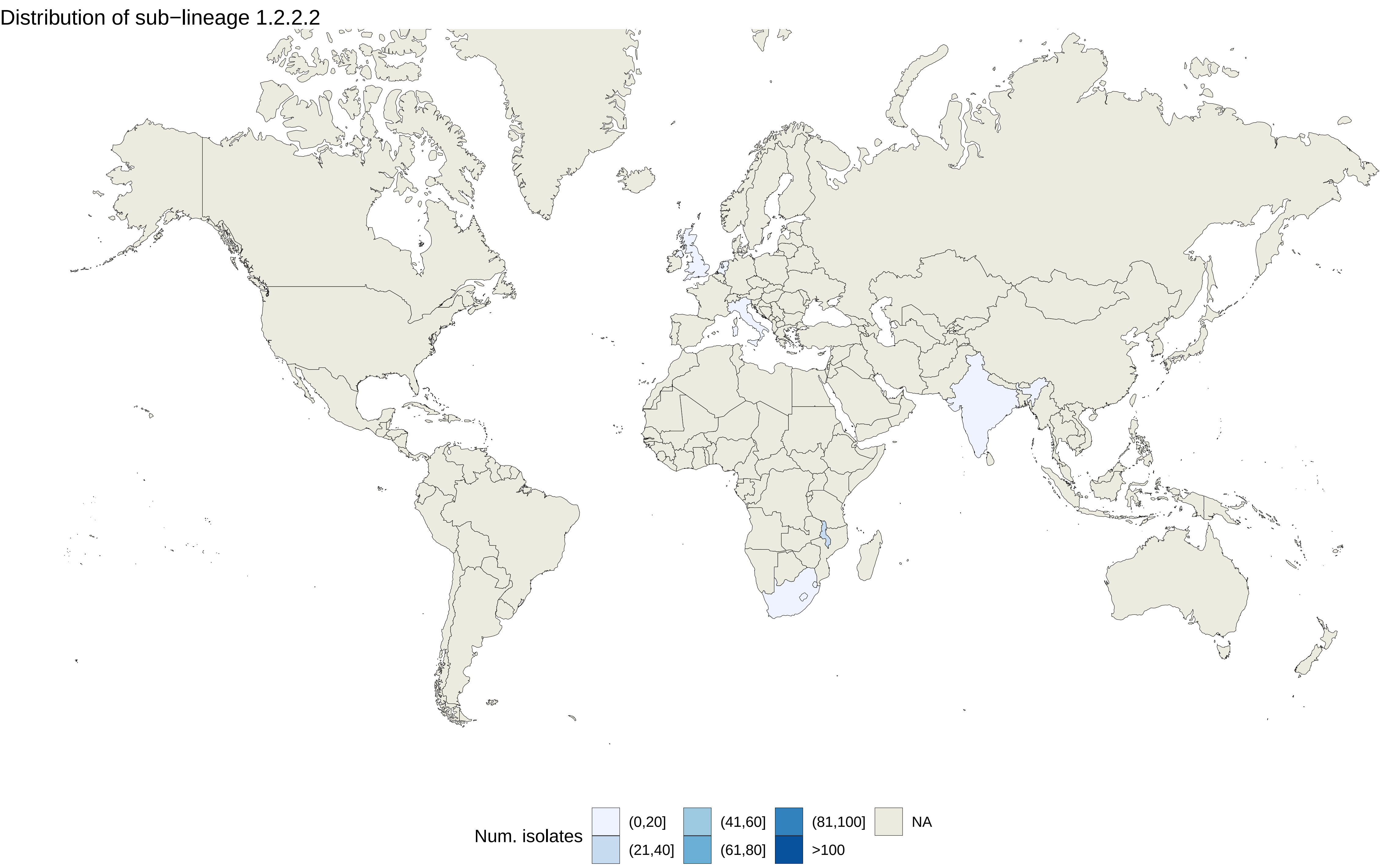
Distribution of sub−lineage 1.2.2.2
Num. isolates
(0,20]
(21,40]
(41,60]
(61,80]
(81,100]
>100
NA

Distribution of sub−lineage 2.1

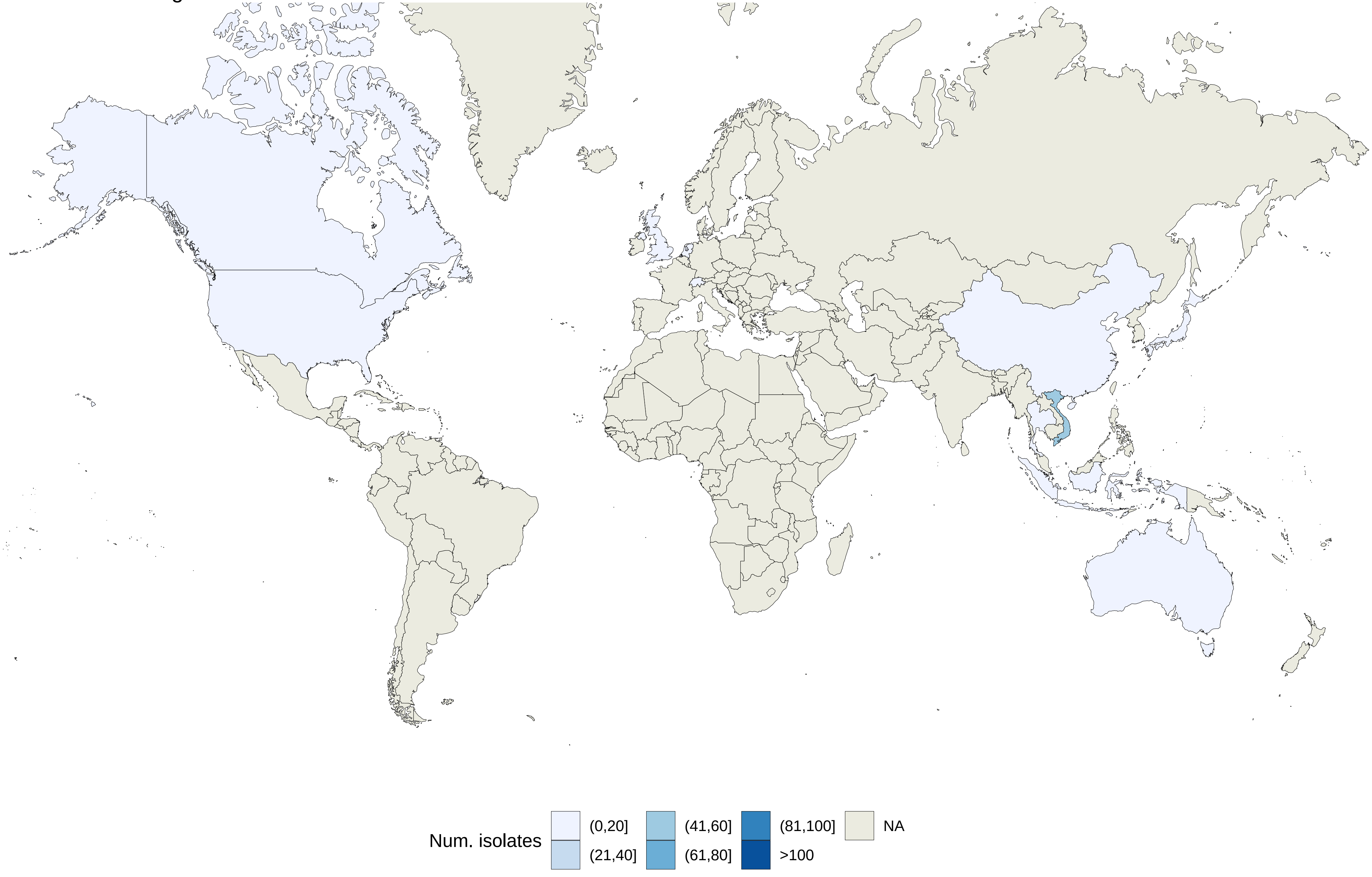

Distribution of sub−lineage 2.2.1.1.1.i1

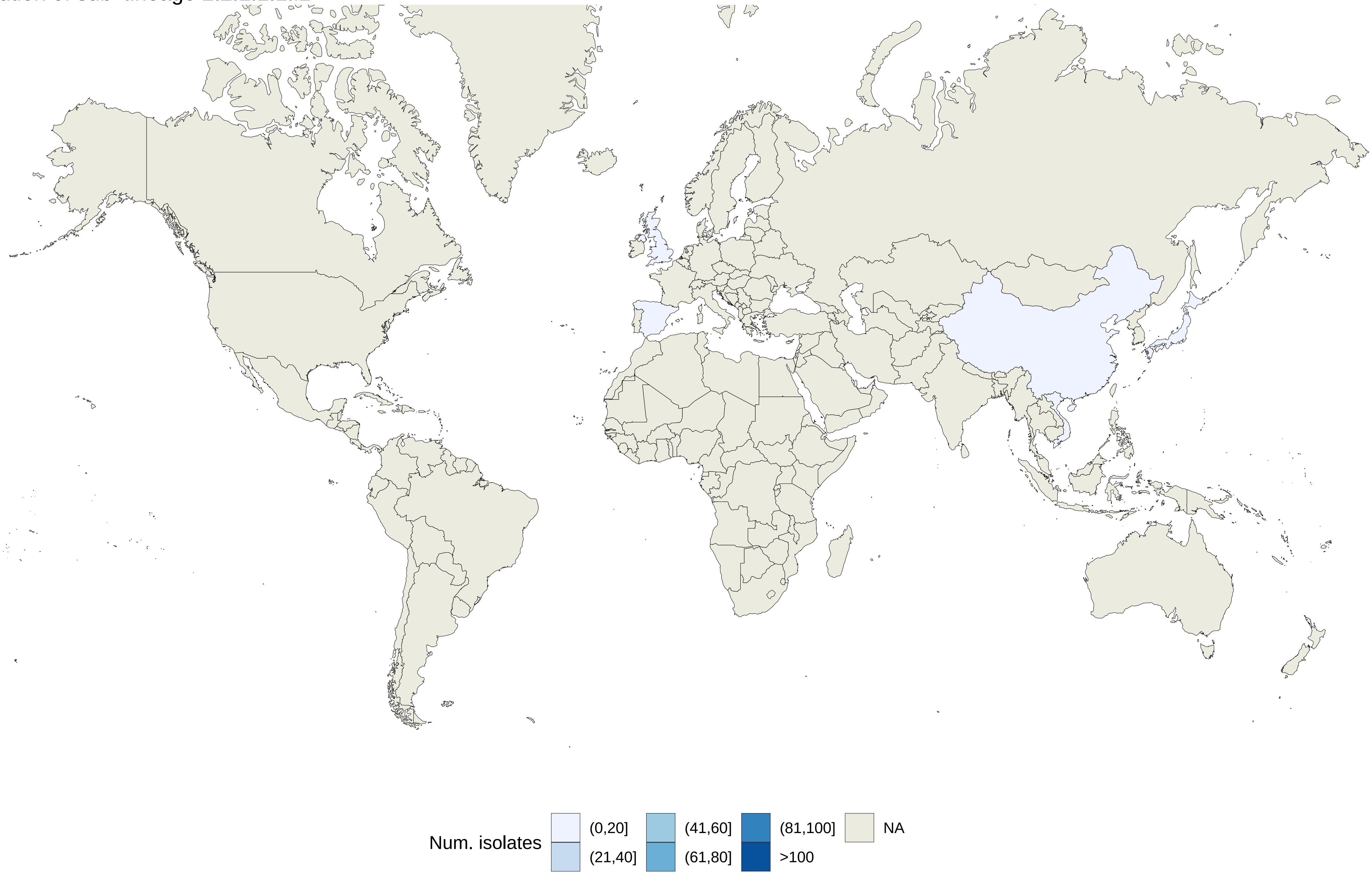

Distribution of sub−lineage 2.2.1.1.1.i2

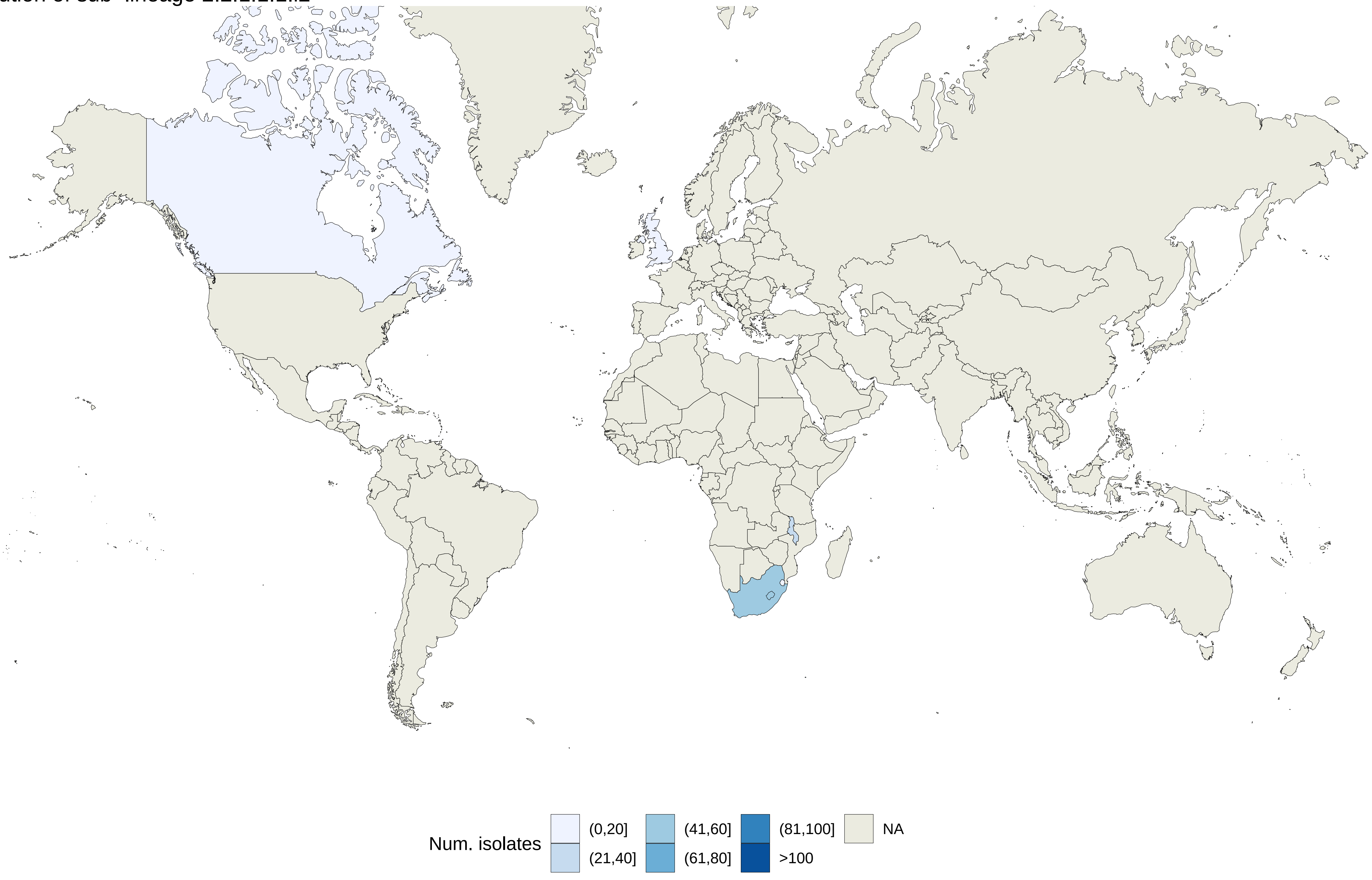

Distribution of sub−lineage 2.2.1.1.1.i3

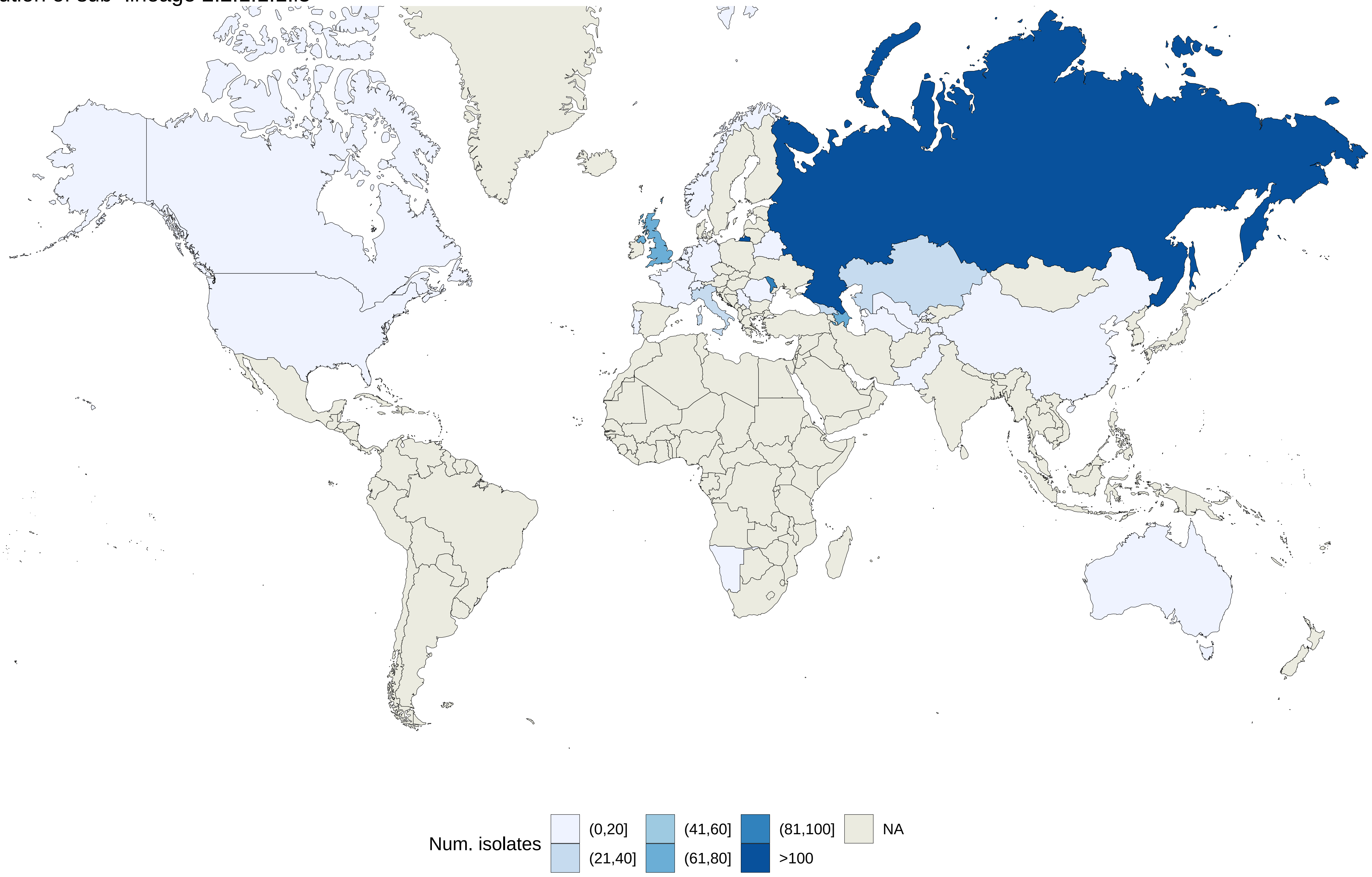

Distribution of sub−lineage 2.2.1.1.1

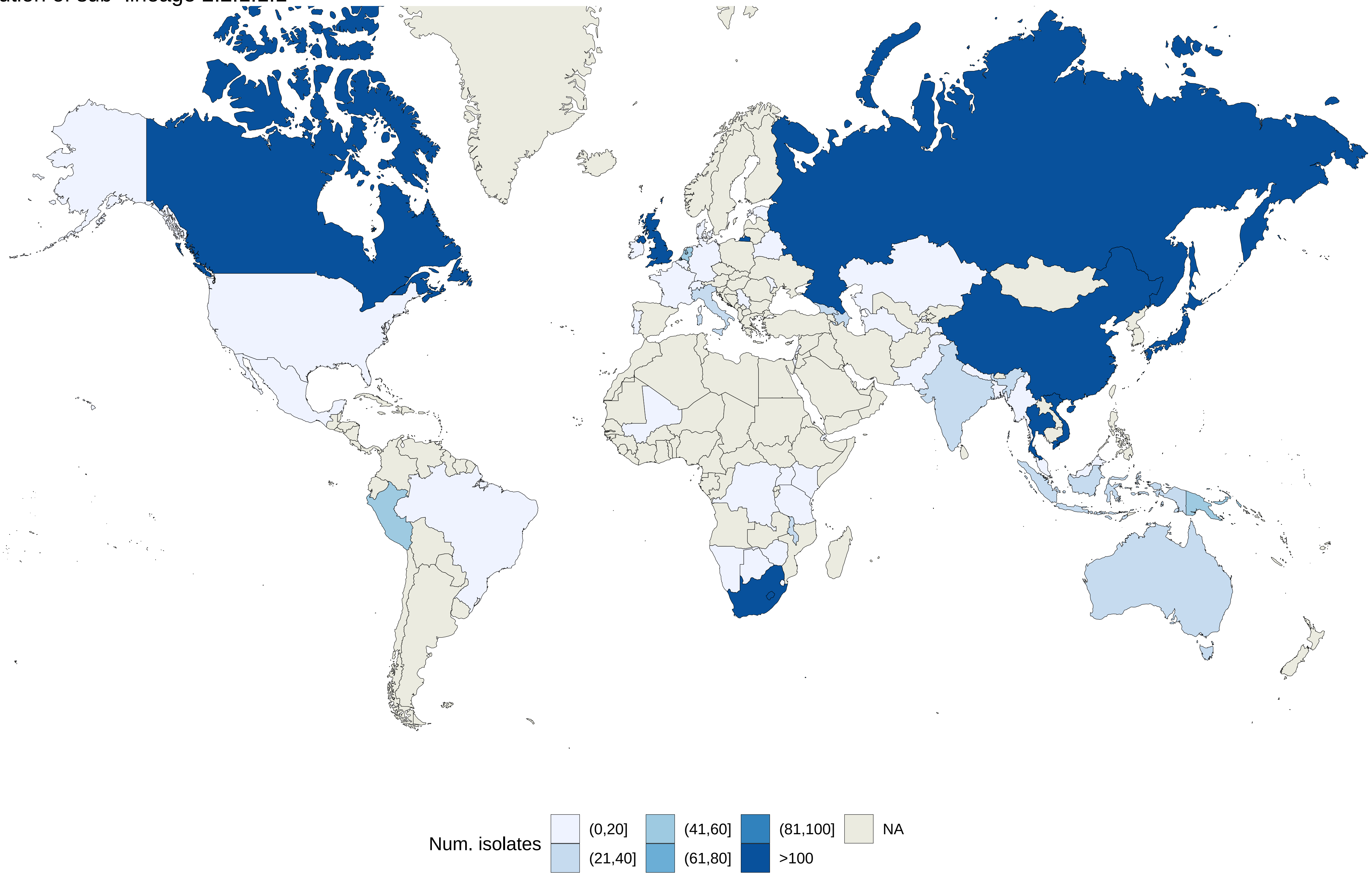

Distribution of sub−lineage 2.2.1.1.2

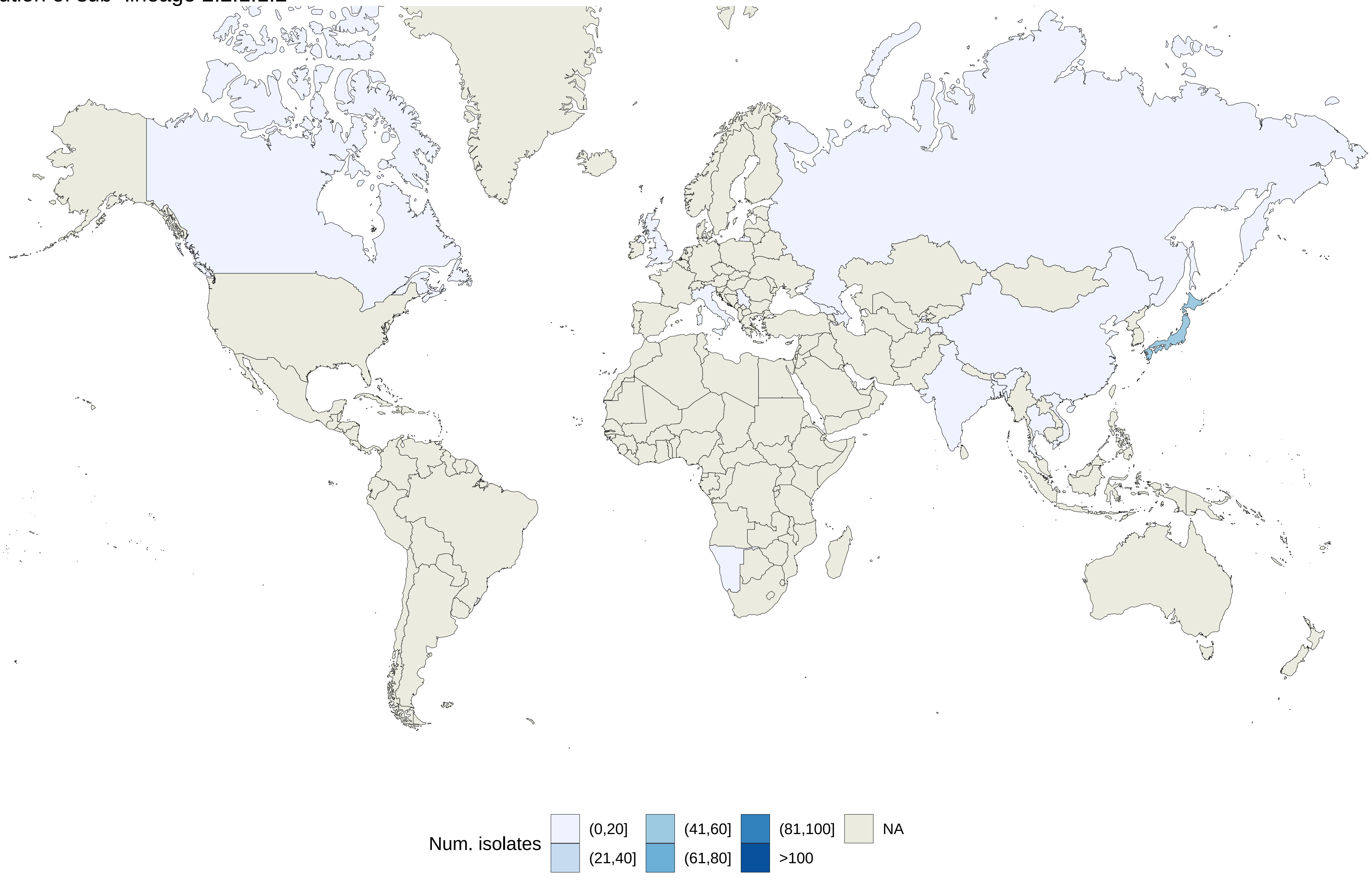

Distribution of sub−lineage 2.2.1.2

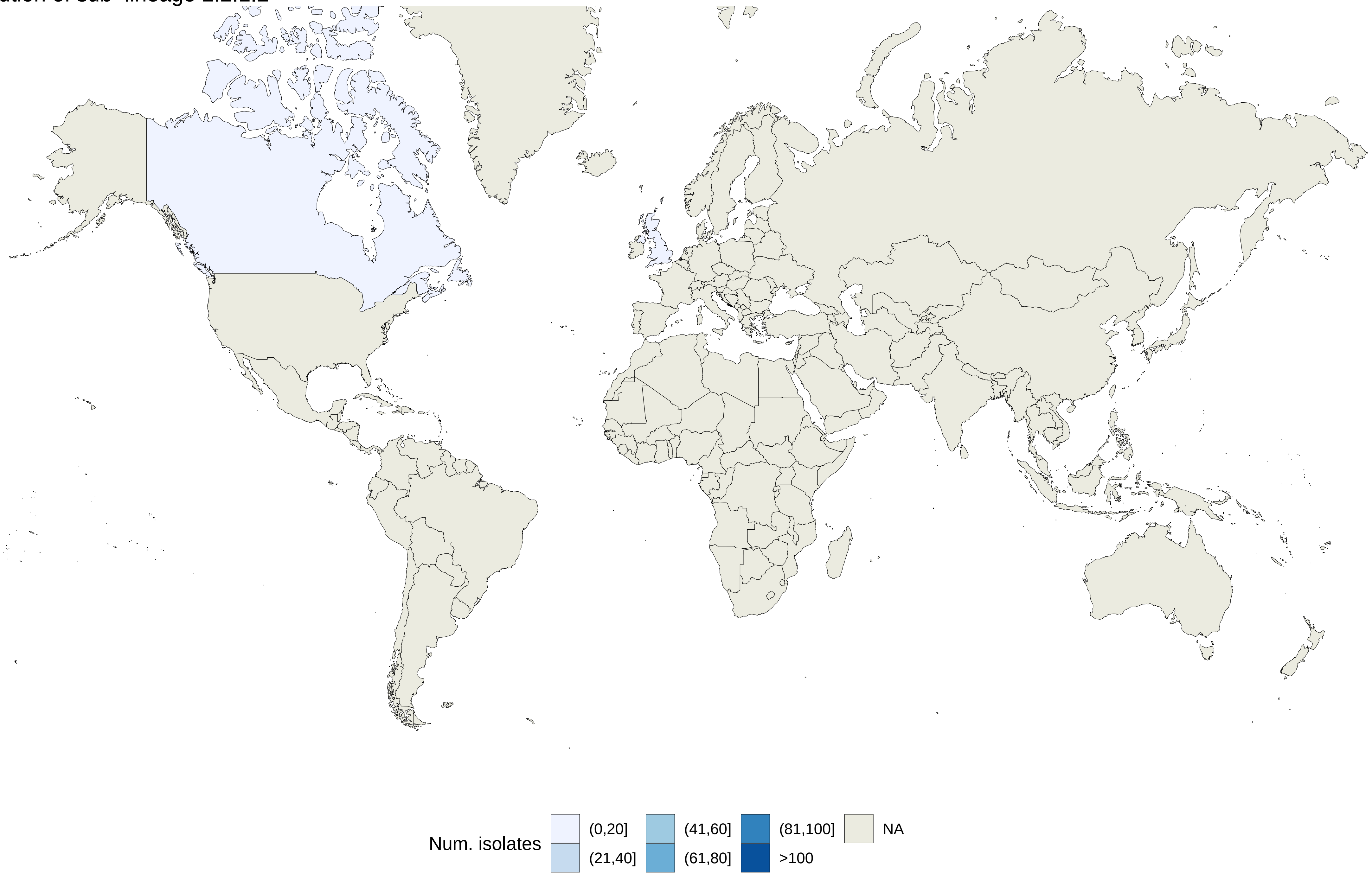

Distribution of sub−lineage 2.2.2

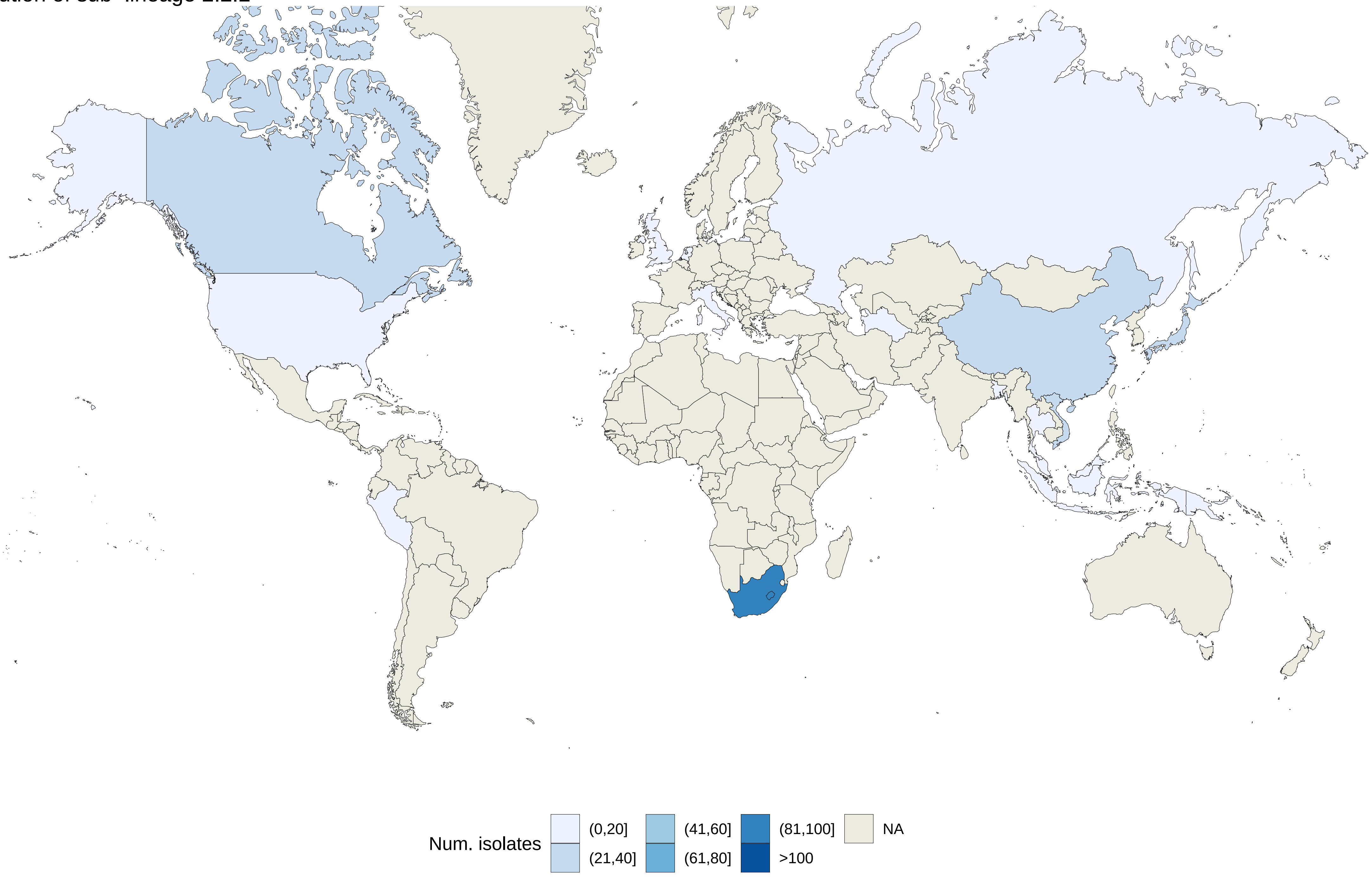

Distribution of sub−lineage 3.1.1.i1

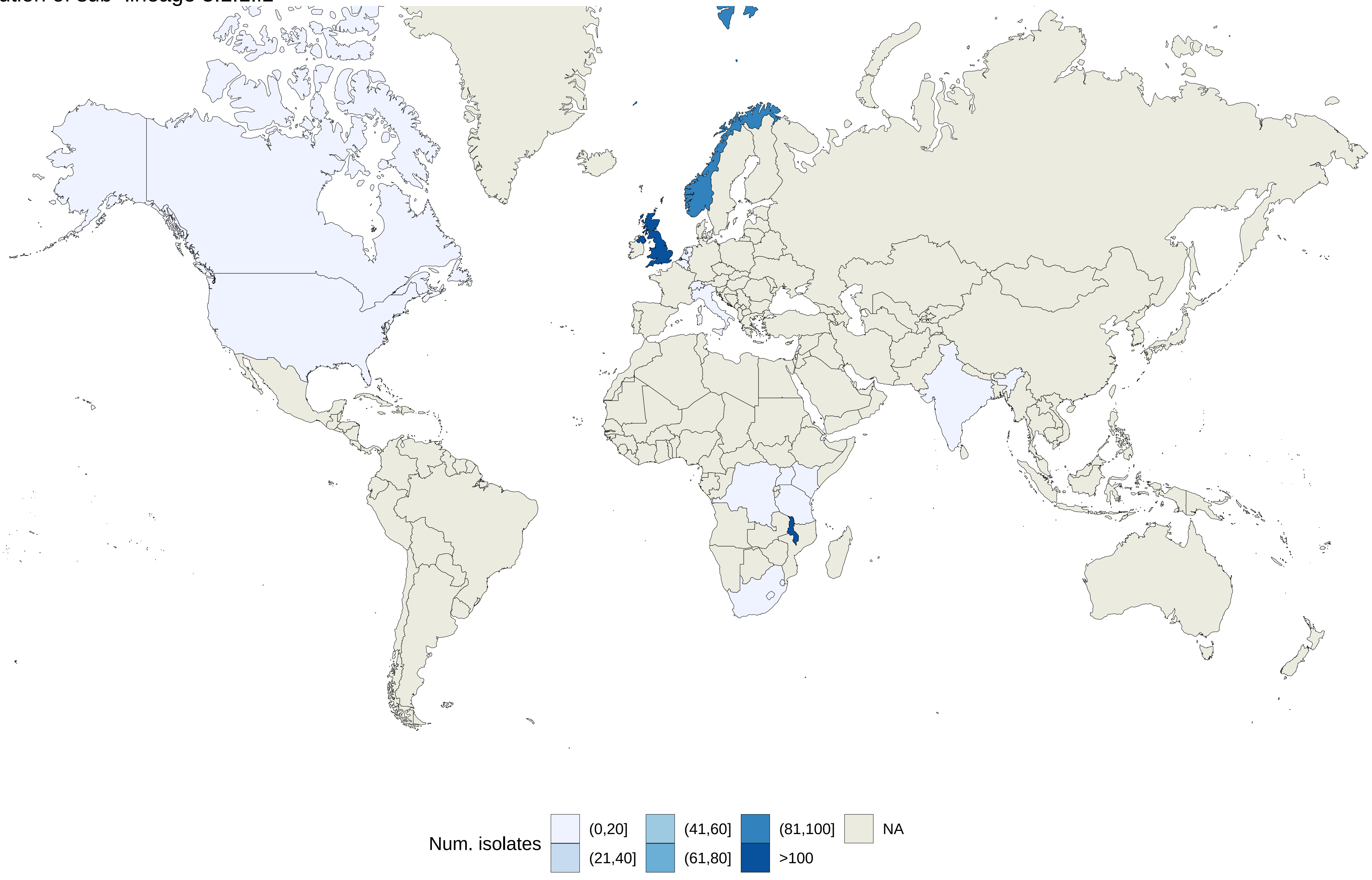

Distribution of sub−lineage 3.1.1.i2

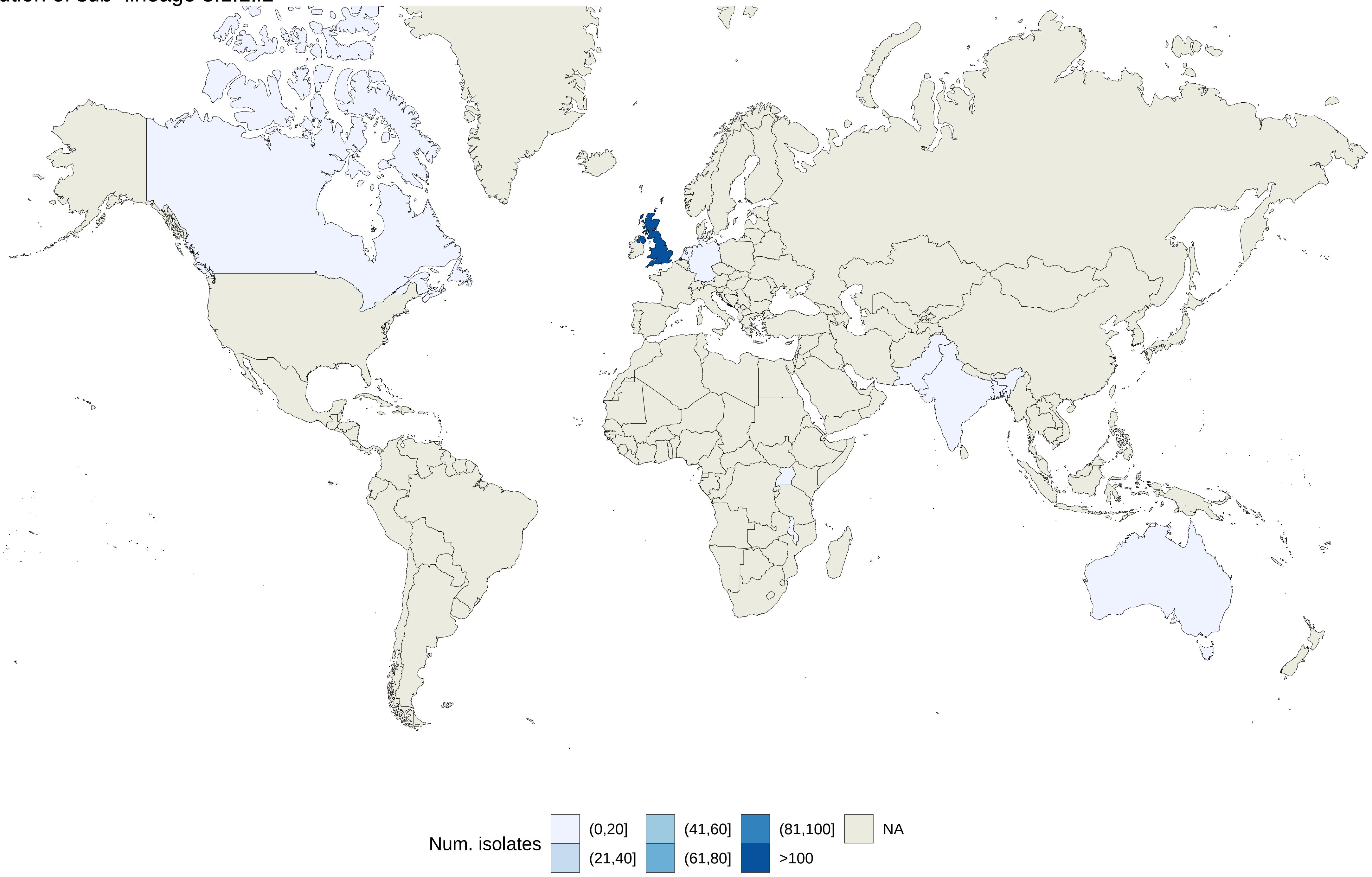

Distribution of sub−lineage 3.1.1

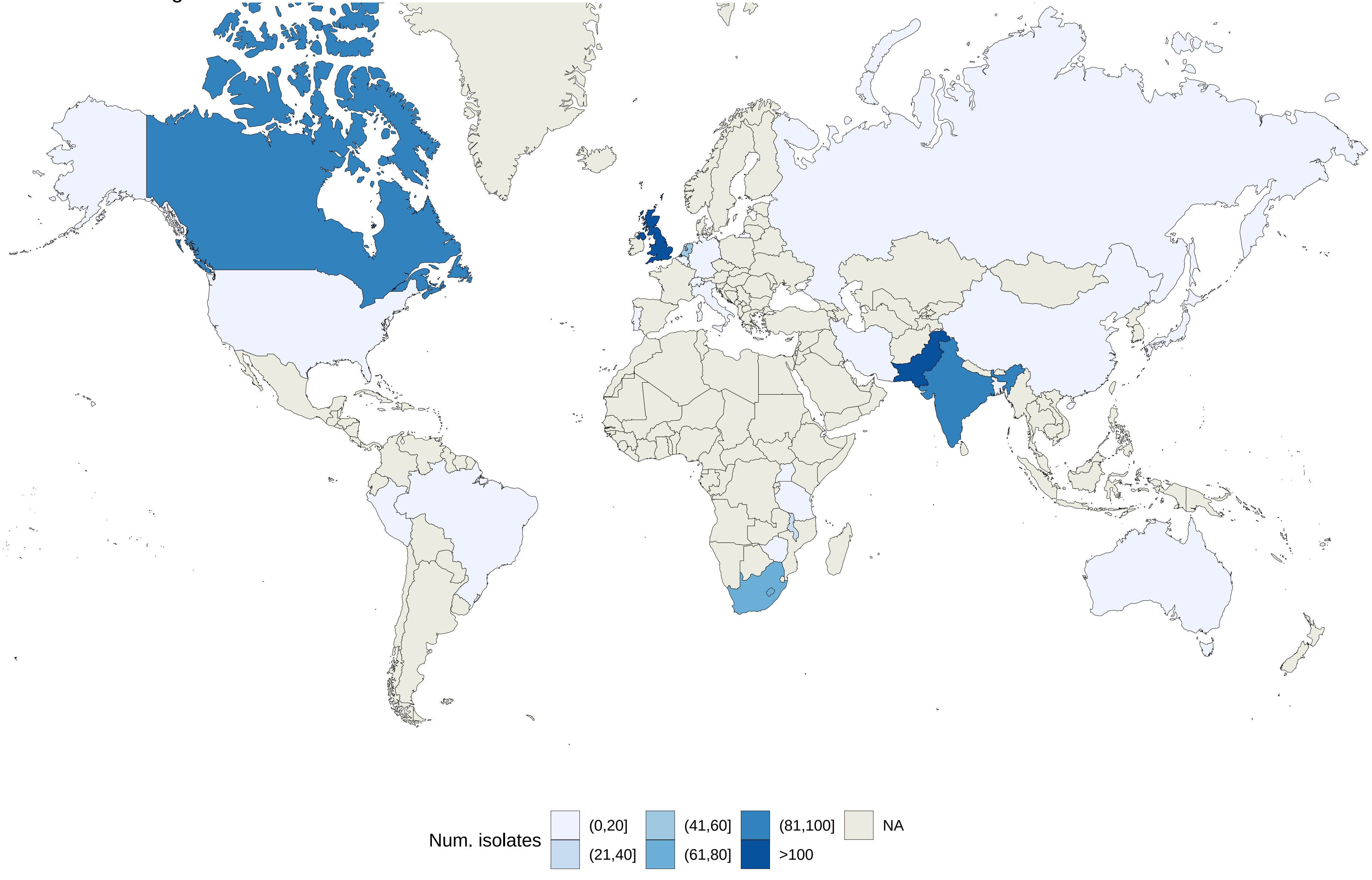

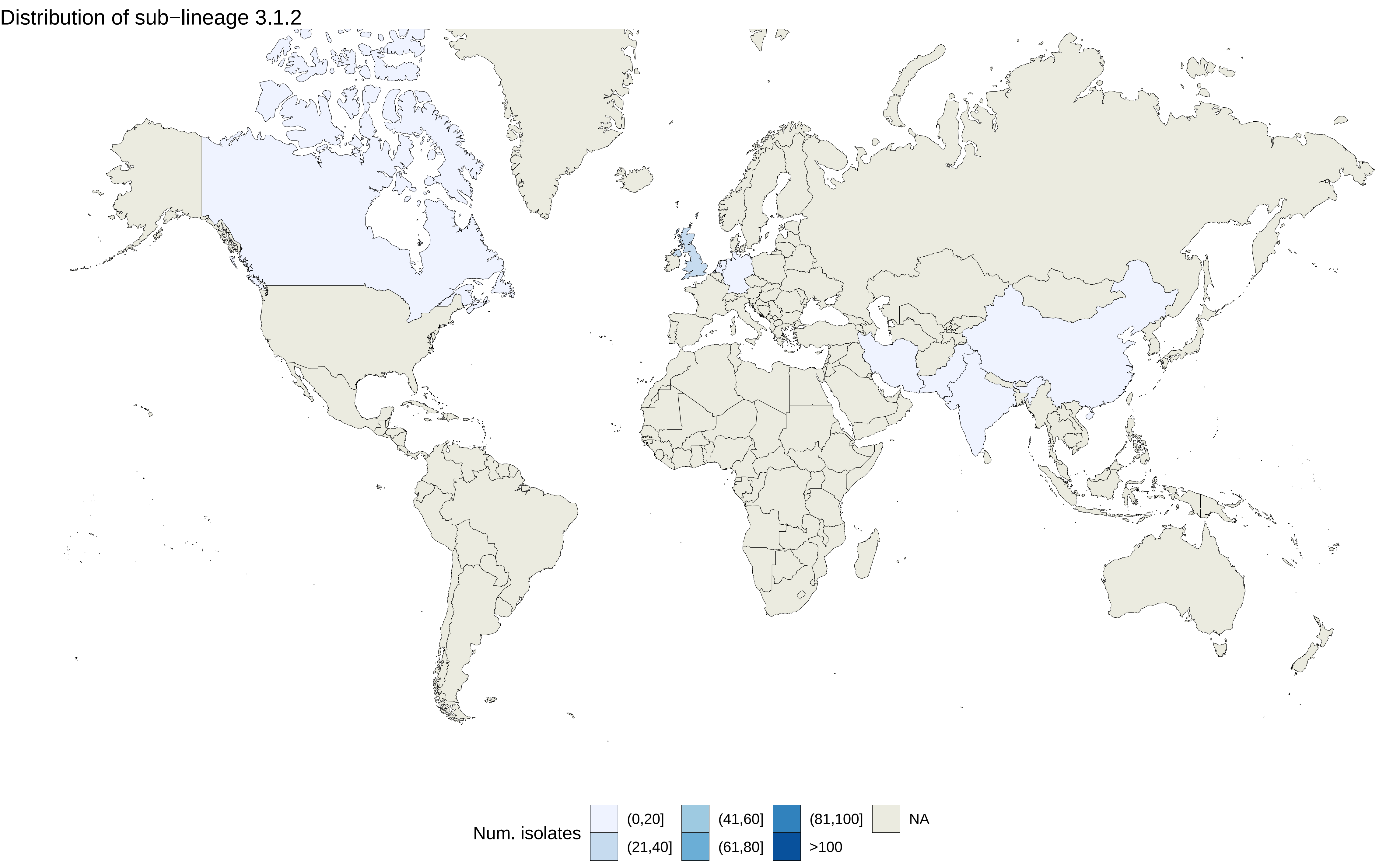
Distribution of sub−lineage 3.1.2
Num. isolates
(0,20]
(21,40]
(41,60]
(61,80]
(81,100]
>100
NA

Distribution of sub−lineage 3.2.1

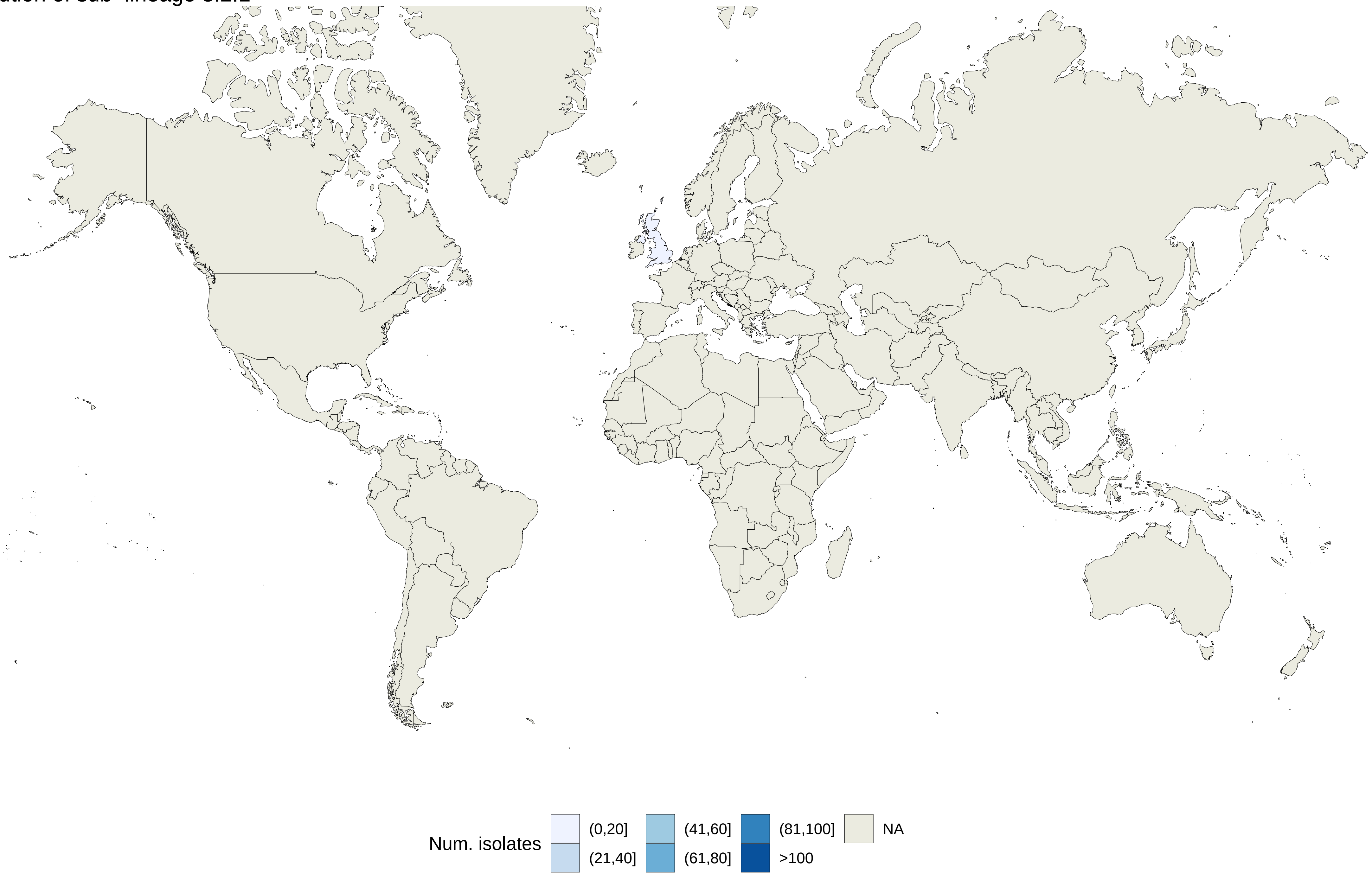

Distribution of sub−lineage 3.2.2

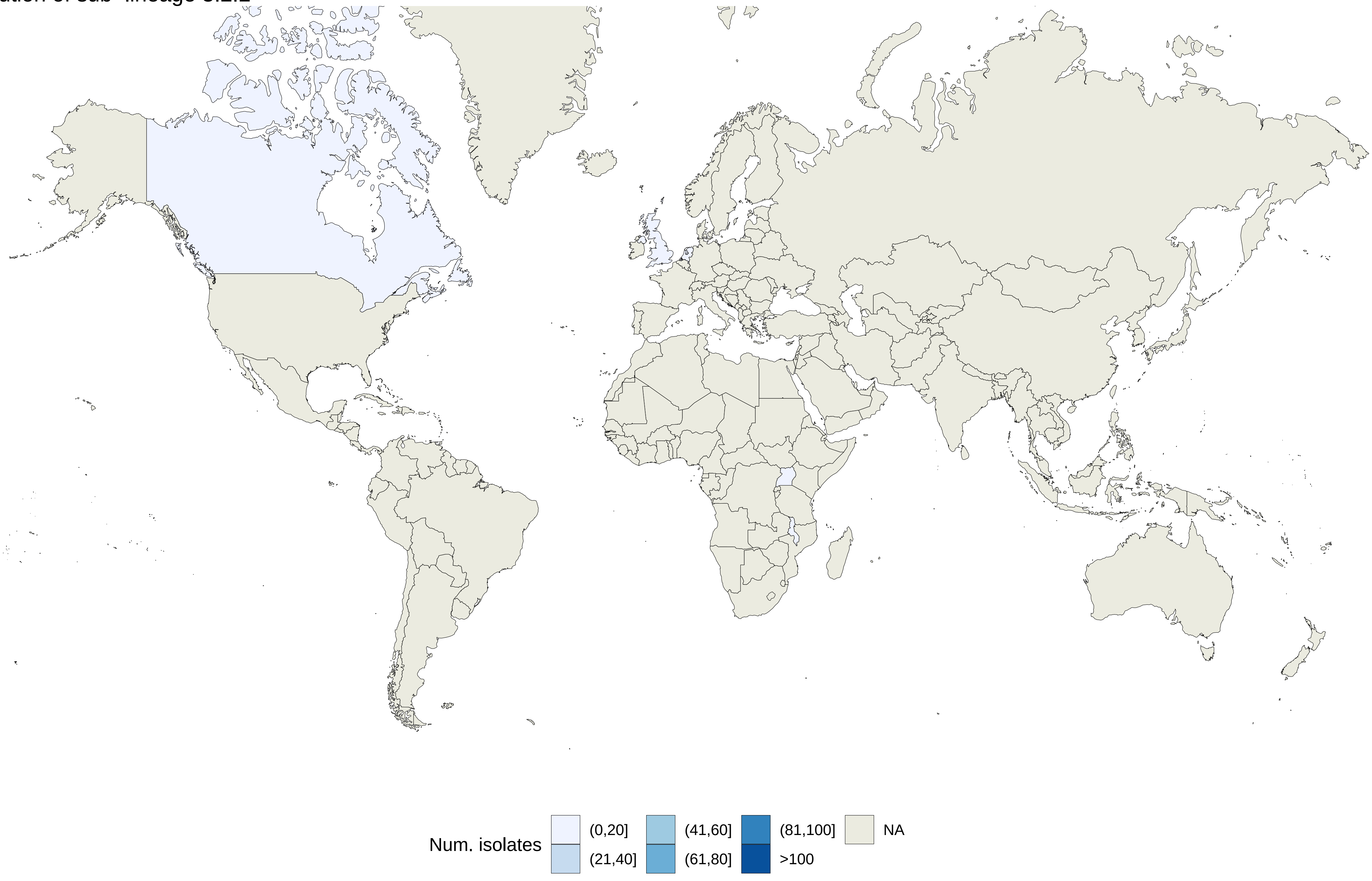

Distribution of sub−lineage 4.1.i1.1.1.1

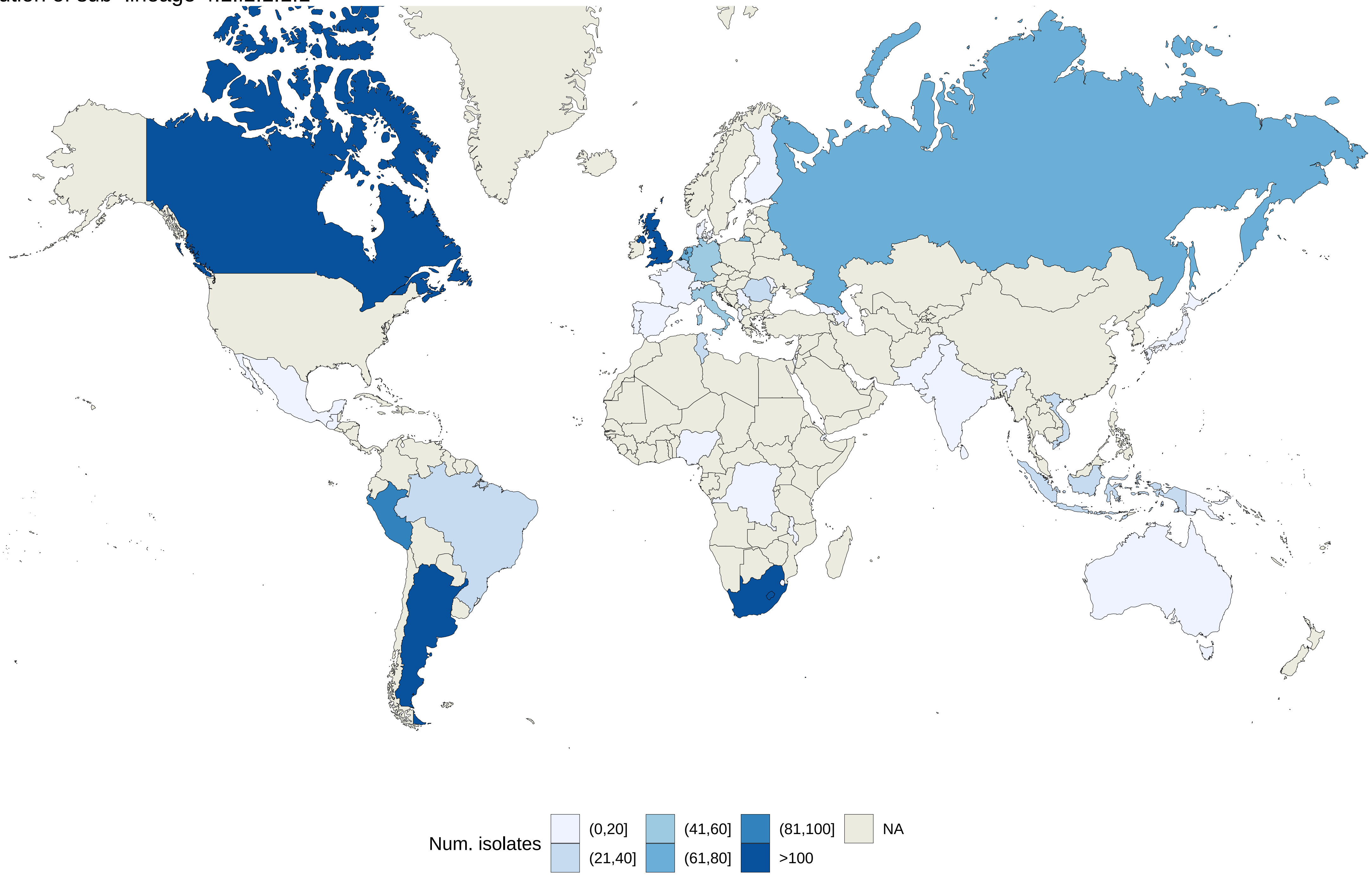

Distribution of sub−lineage 4.1.i1.1.1.2

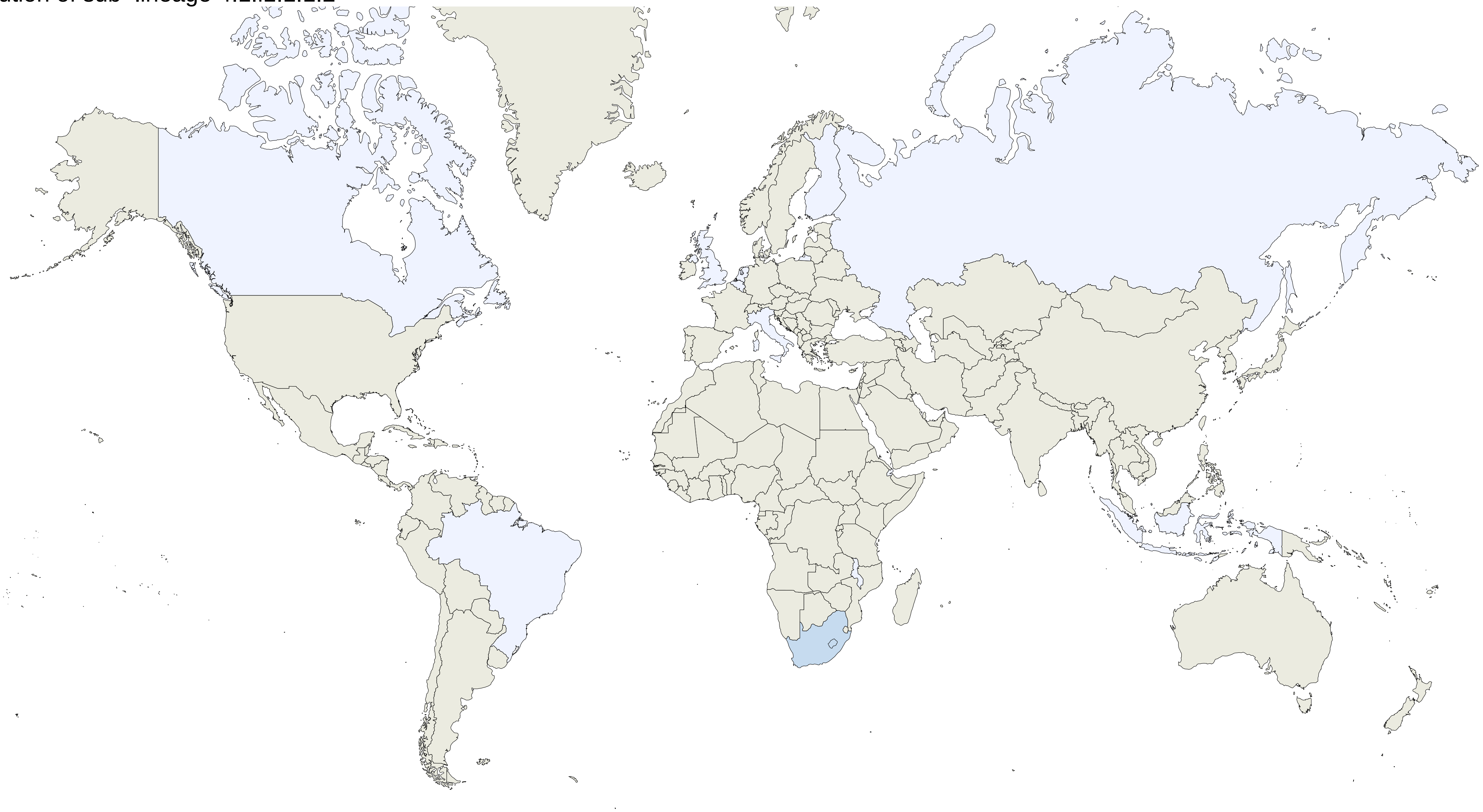

Distribution of sub−lineage 4.1.i1.1.2

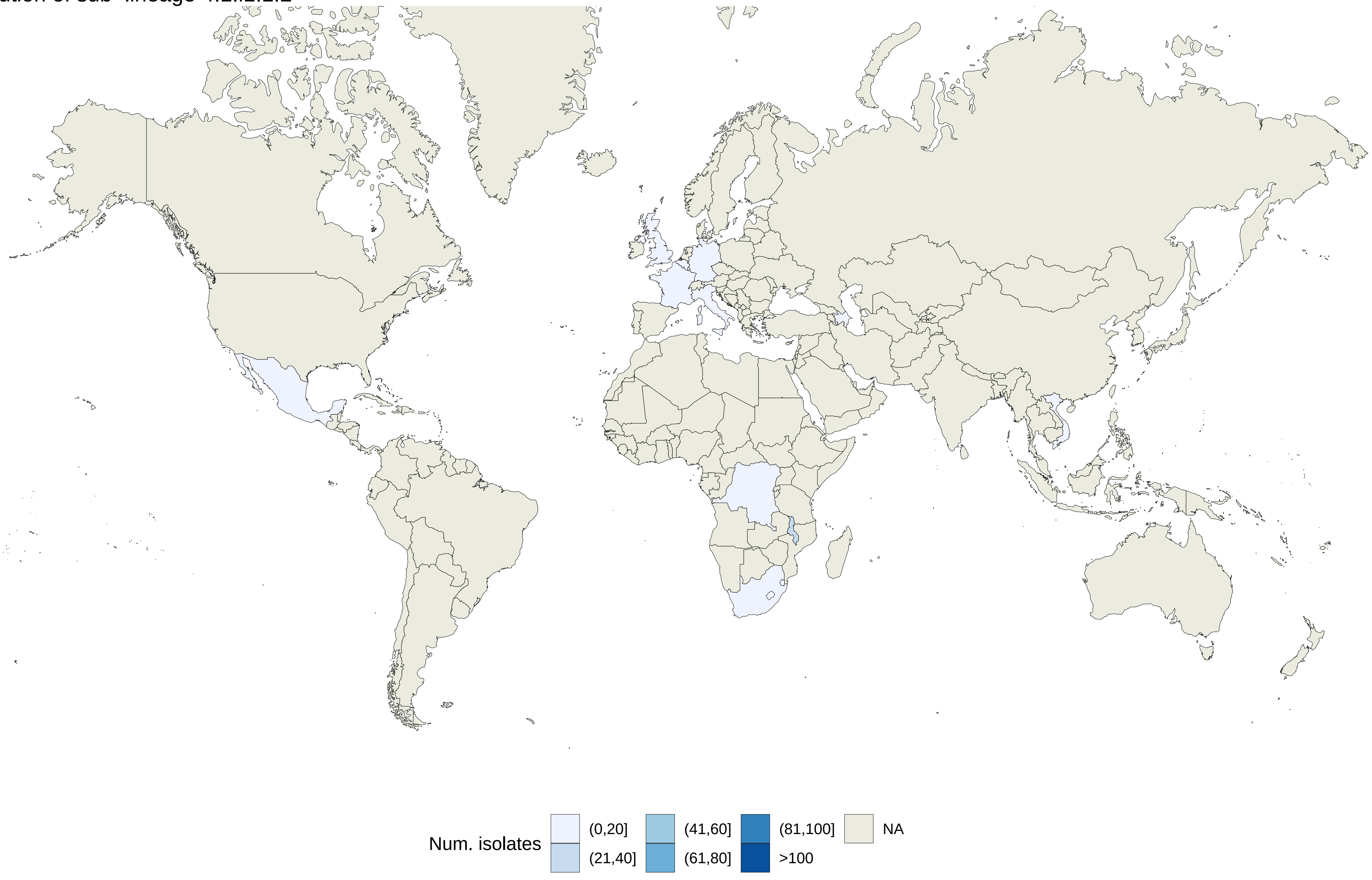

Distribution of sub−lineage 4.1.i1.2.1

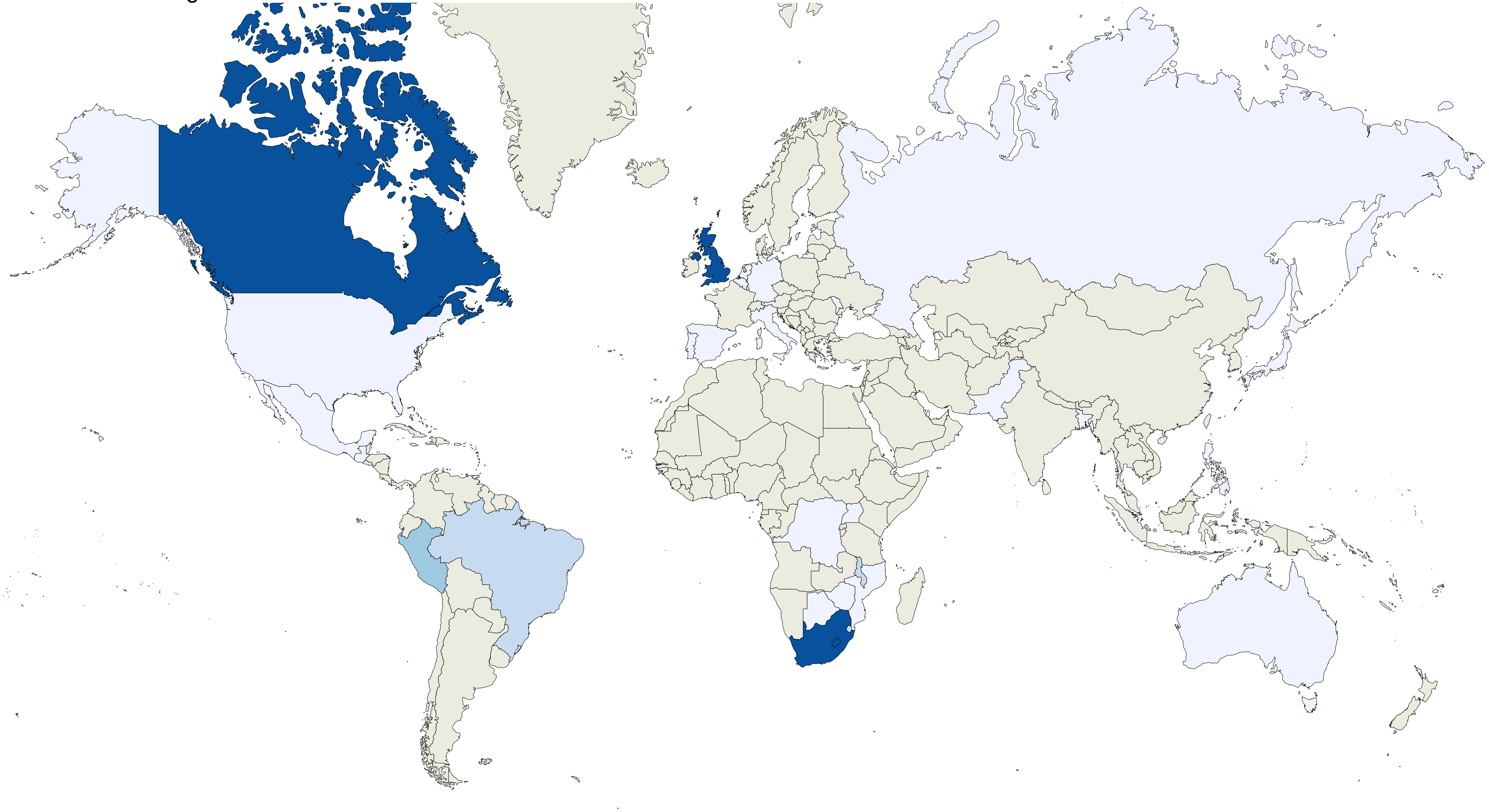

Num. isolates
(0,20]
(21,40]
(41,60]
(61,80]
(81,100]
>100
NA

Distribution of sub−lineage 4.1.i1.2.2

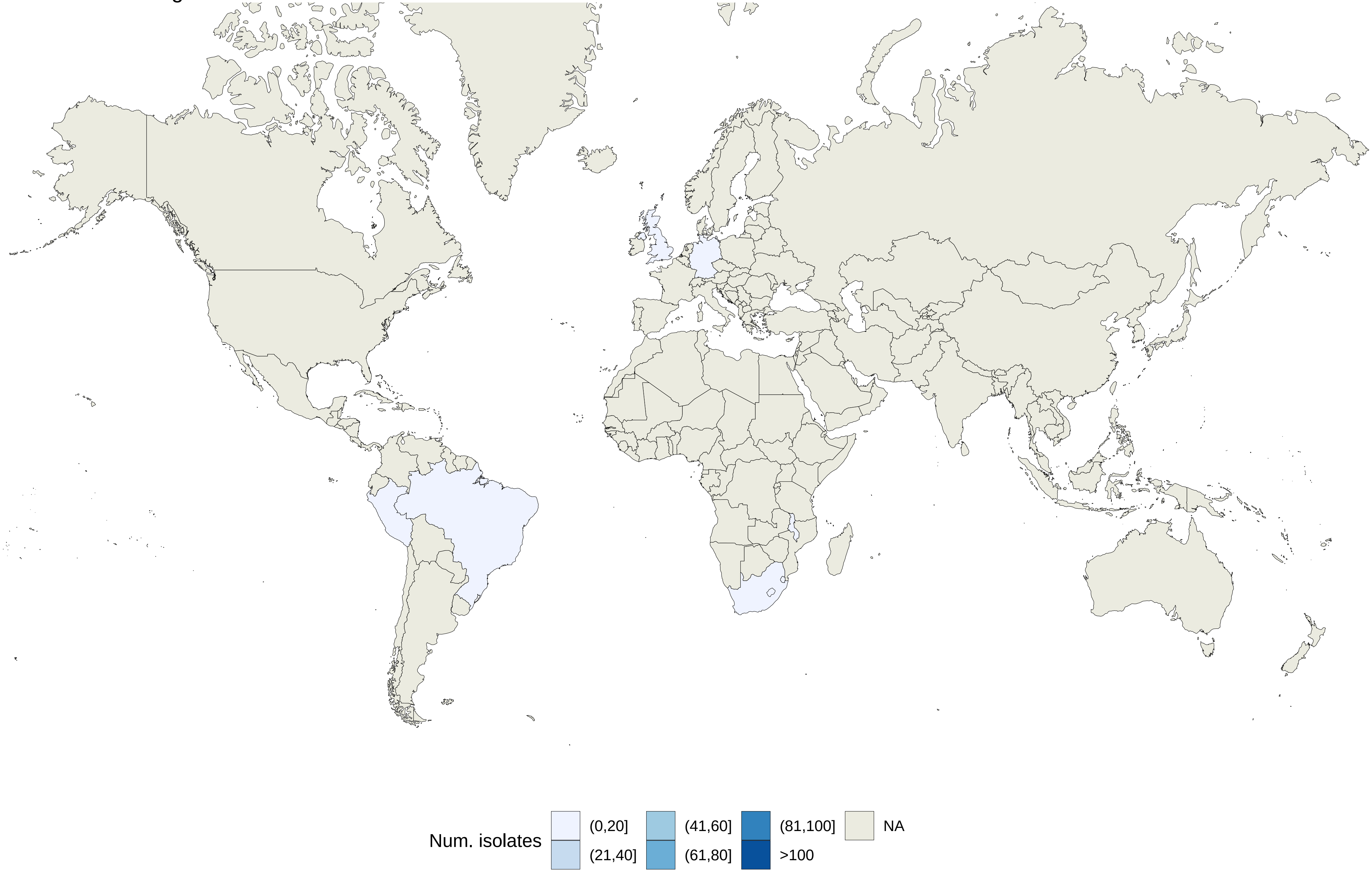

Distribution of sub−lineage 4.1.i2.1

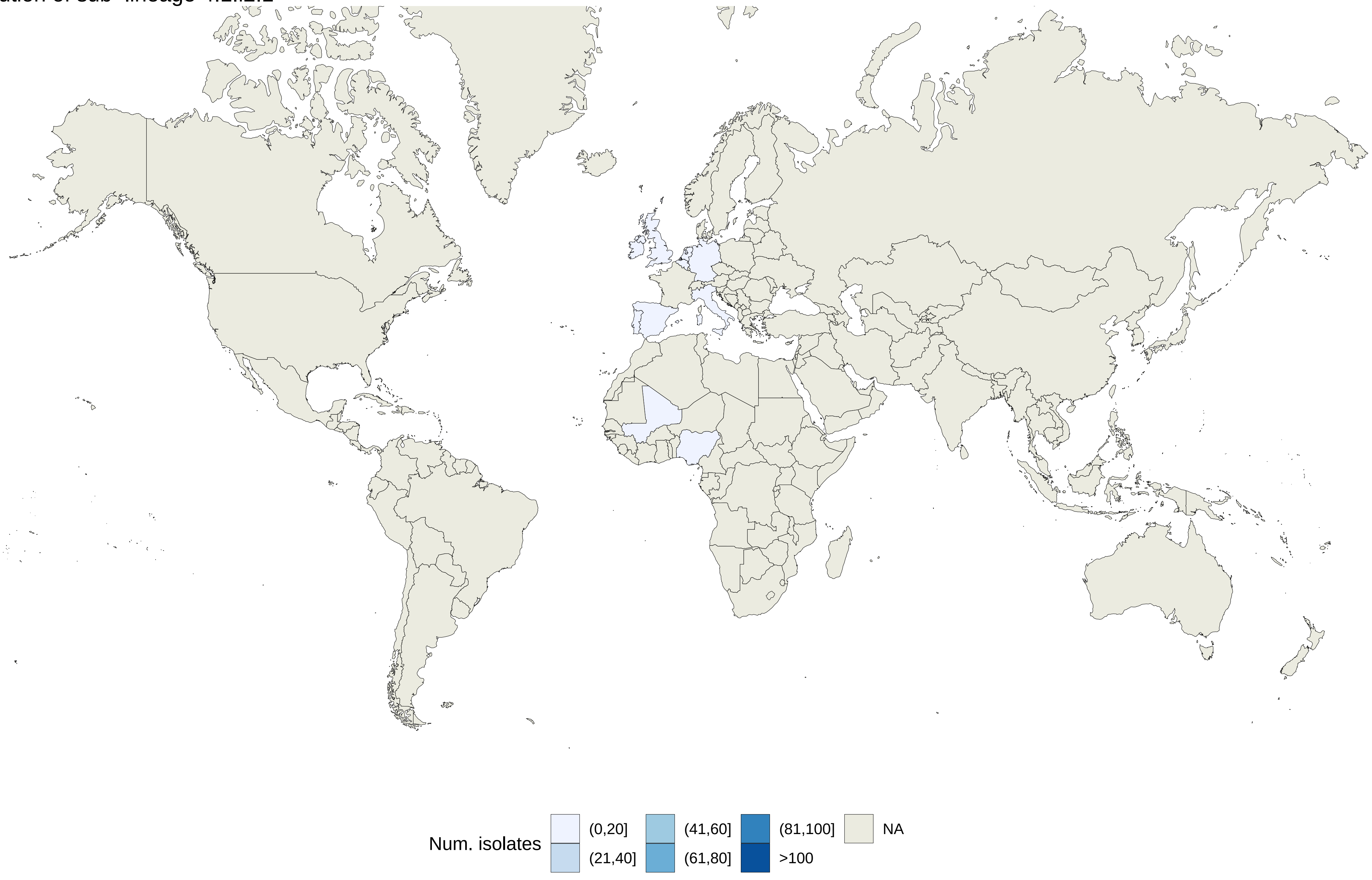

Distribution of sub−lineage 4.1.i2.2

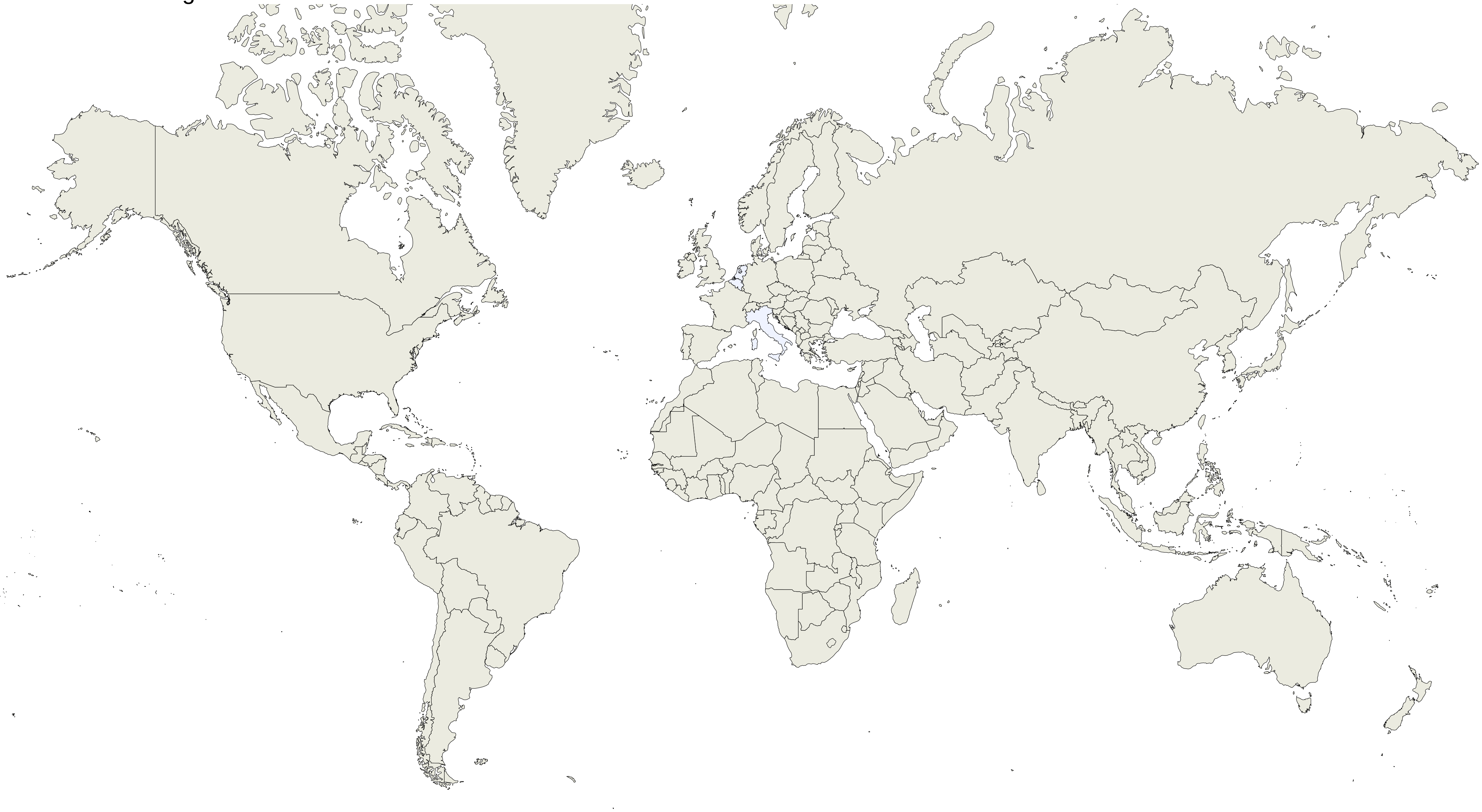

Distribution of sub−lineage 4.10.i1

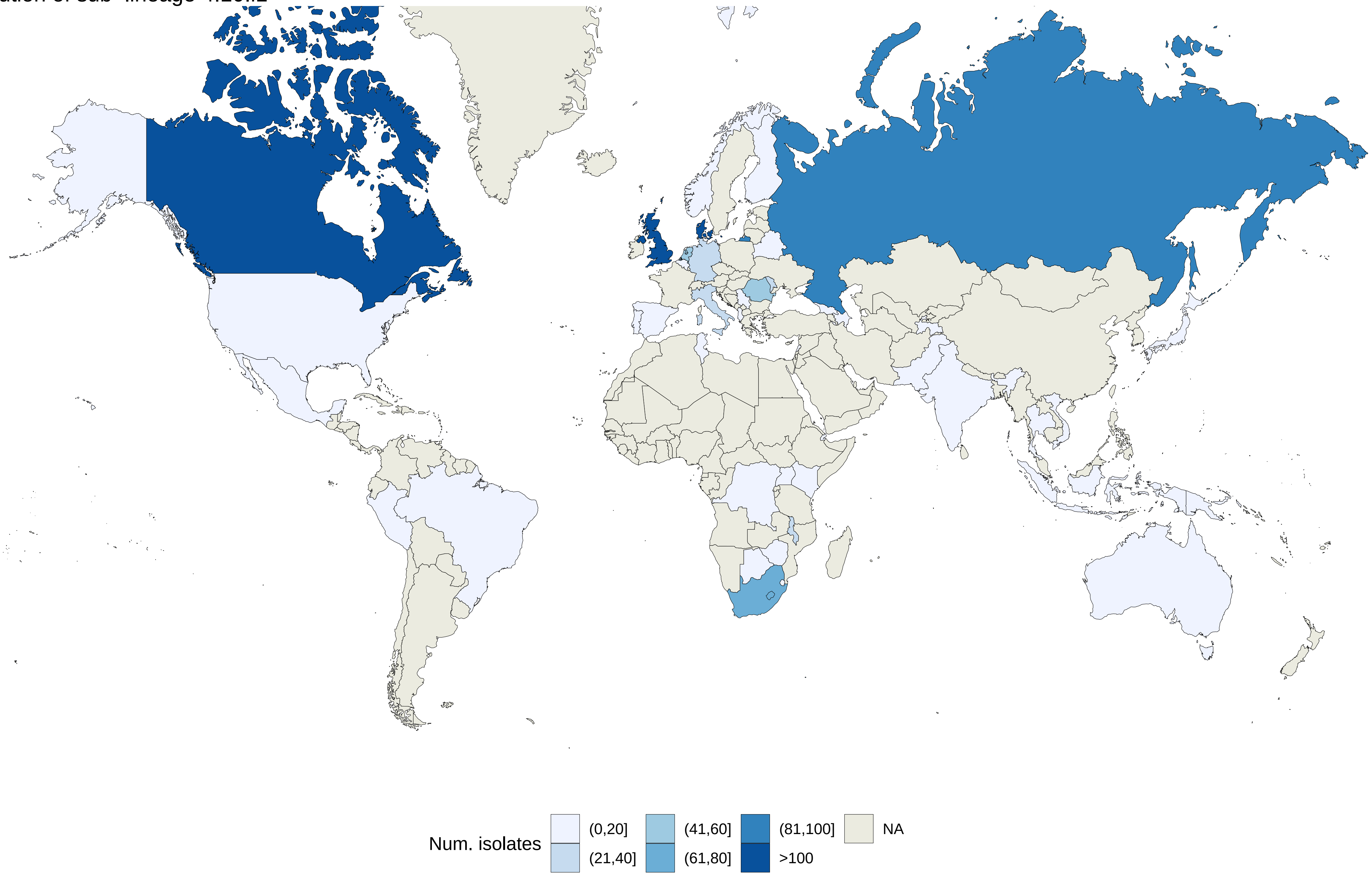

Distribution of sub−lineage 4.10.i2

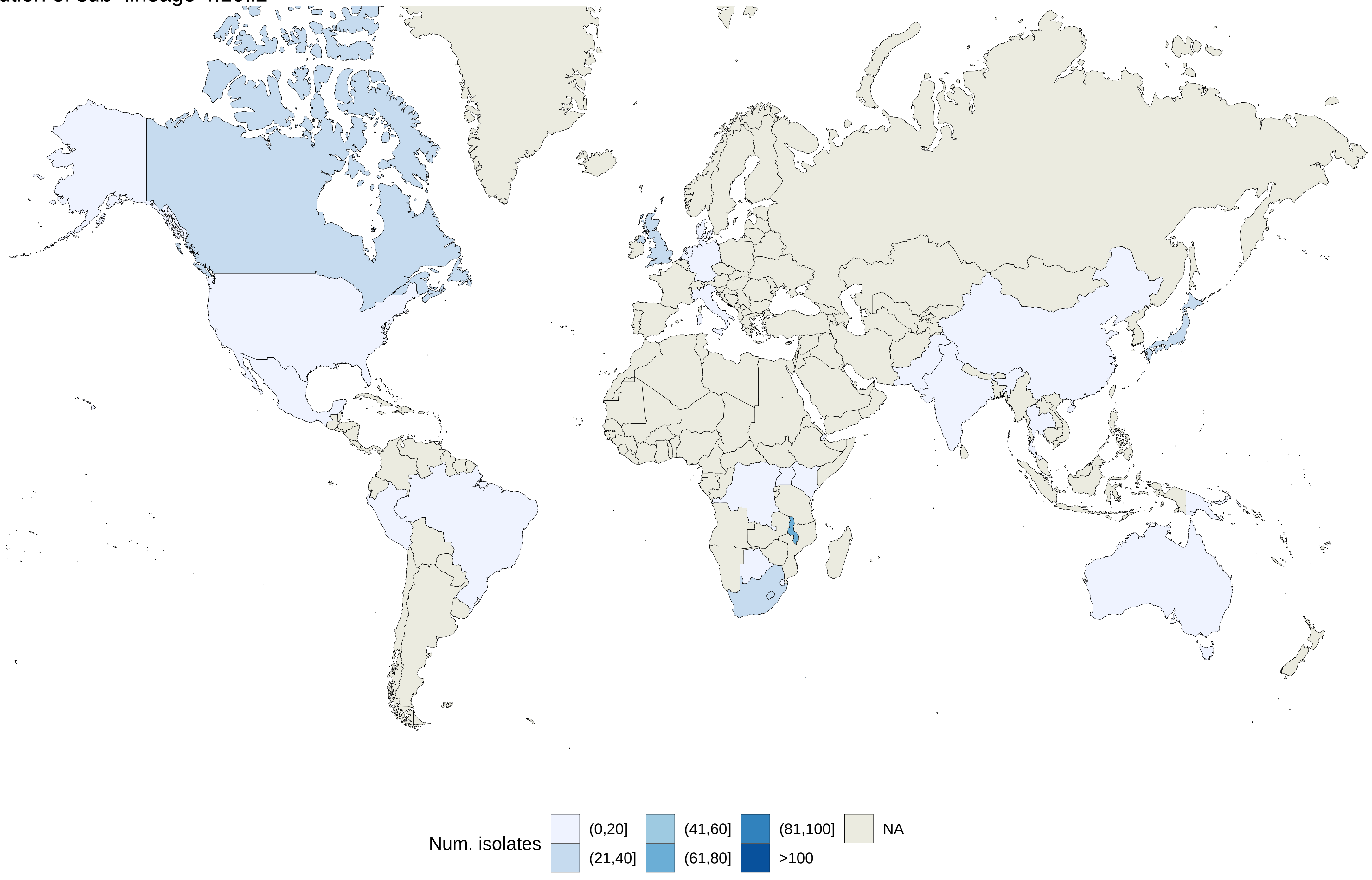

Distribution of sub−lineage 4.11

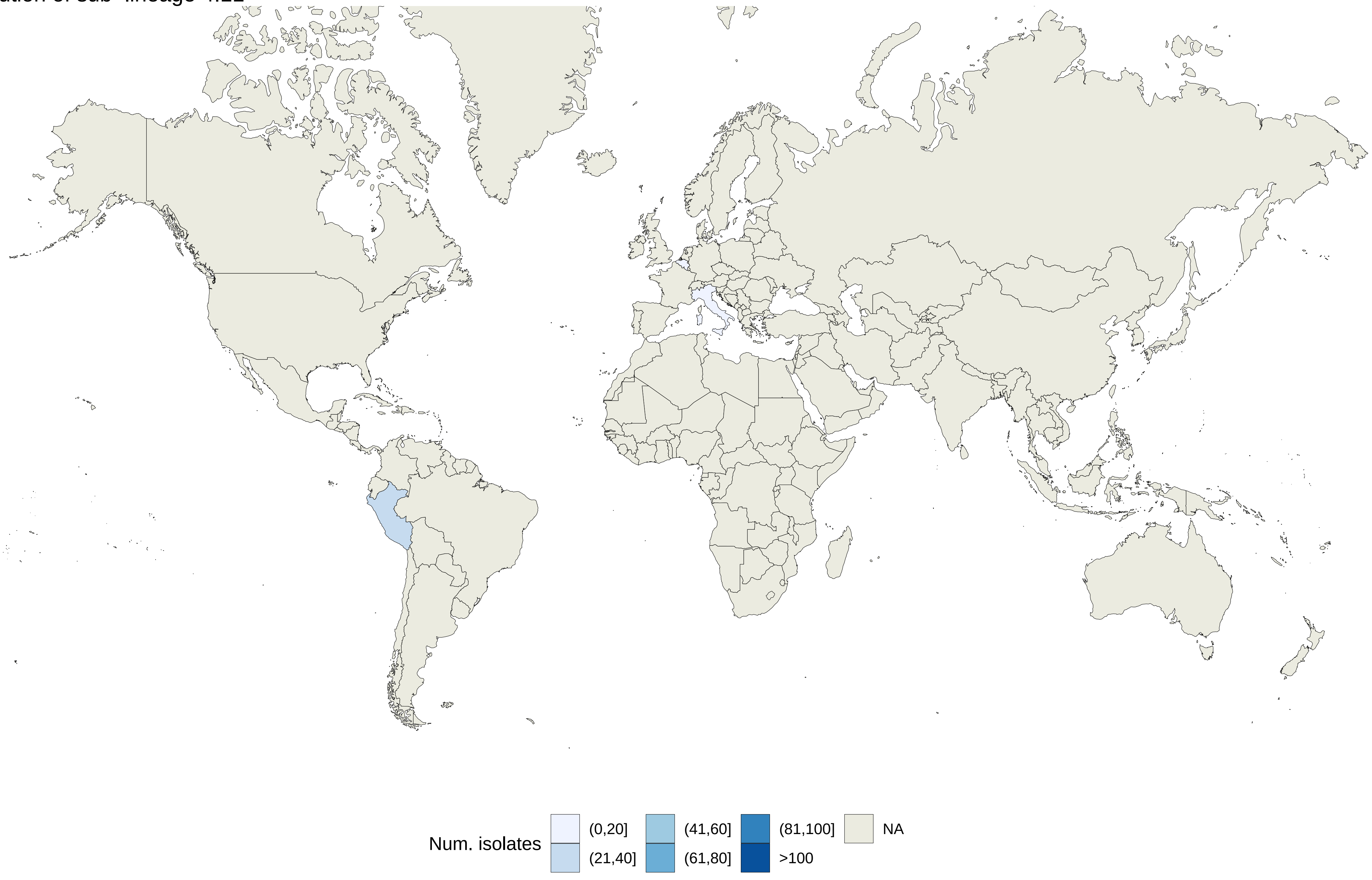

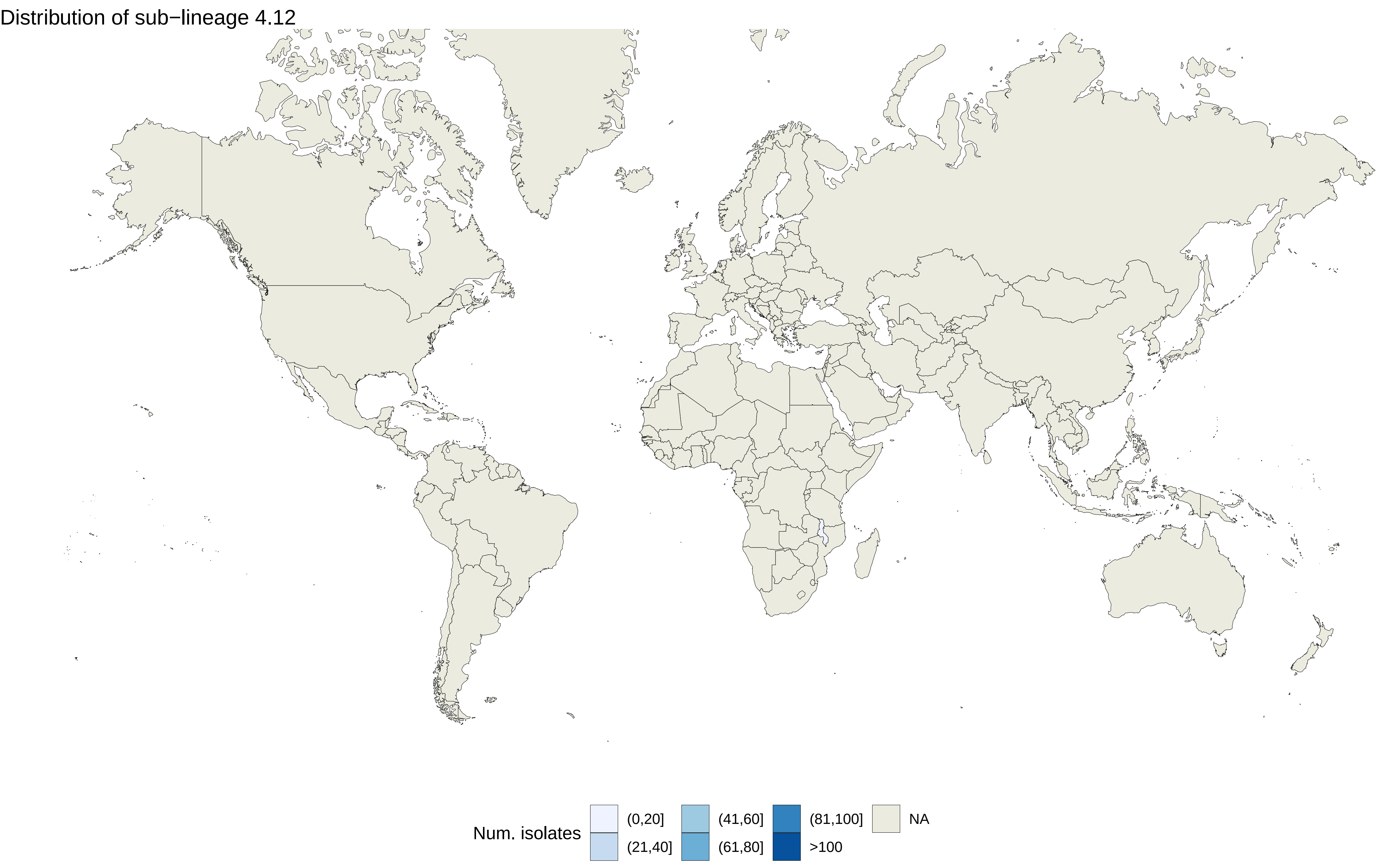
Distribution of sub−lineage 4.12
Num. isolates
(0,20]
(21,40]
(41,60]
(61,80]
(81,100]
>100
NA

Distribution of sub−lineage 4.13

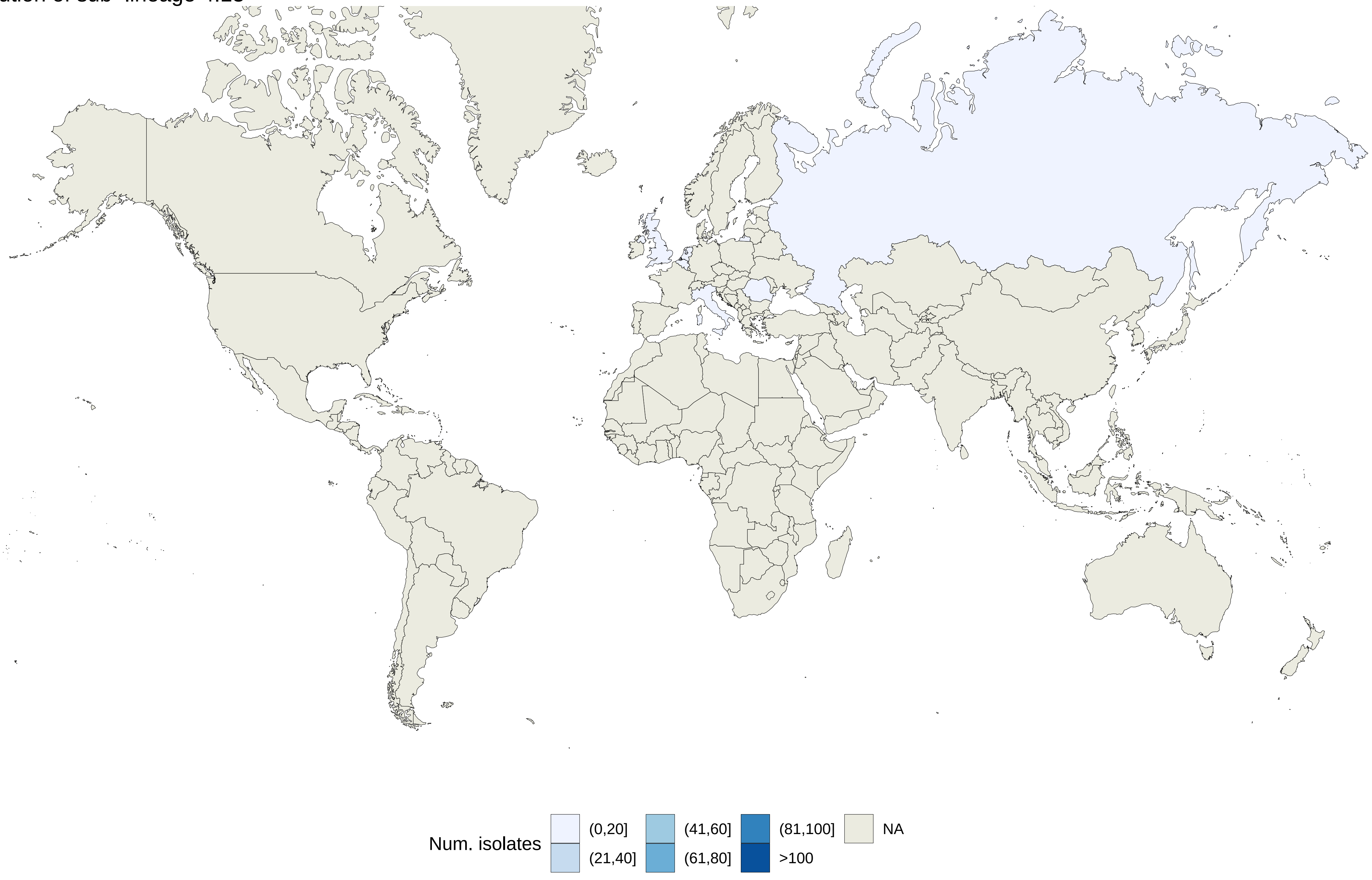

Distribution of sub−lineage 4.1

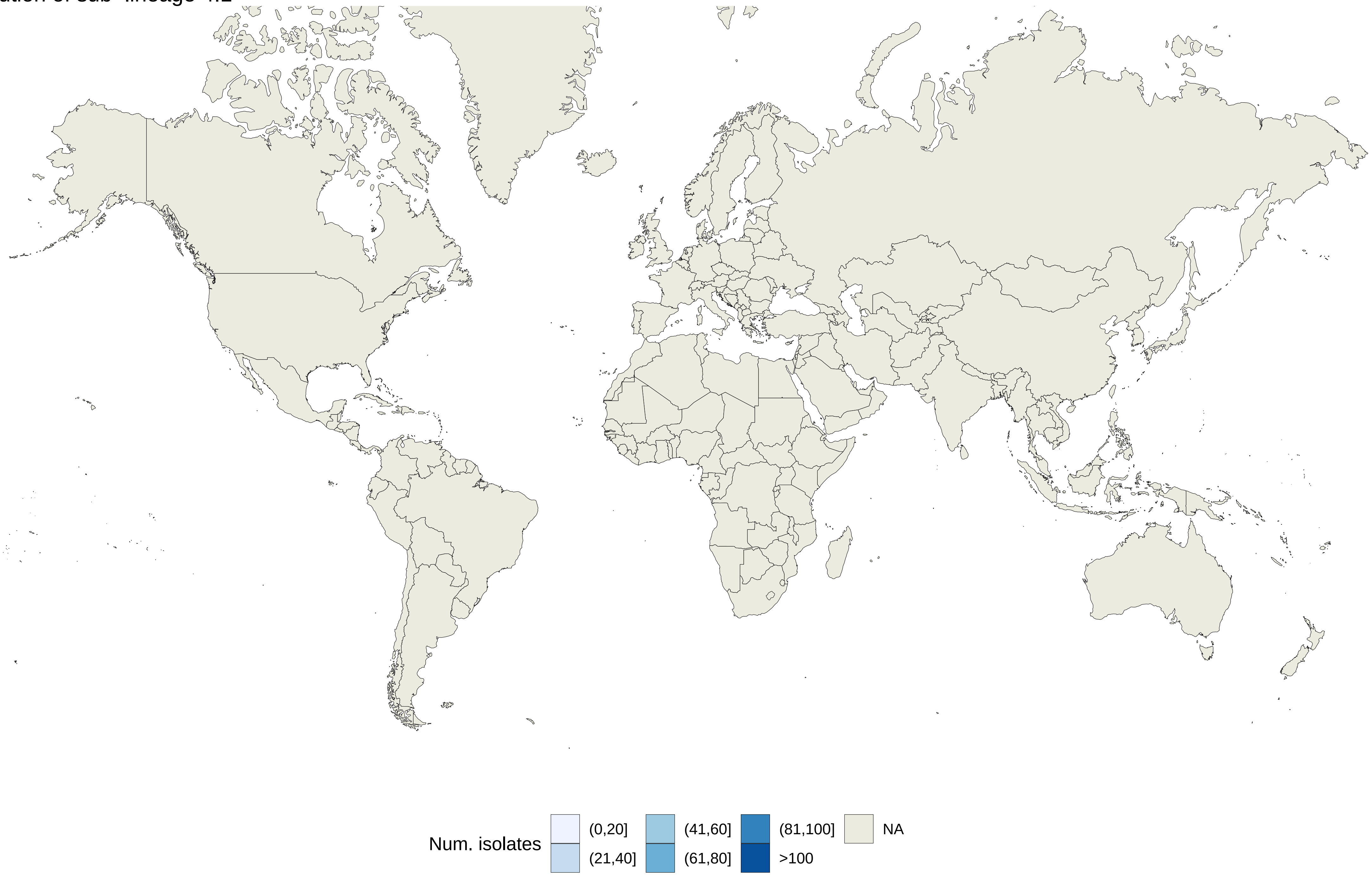

Distribution of sub−lineage 4.2.1.1

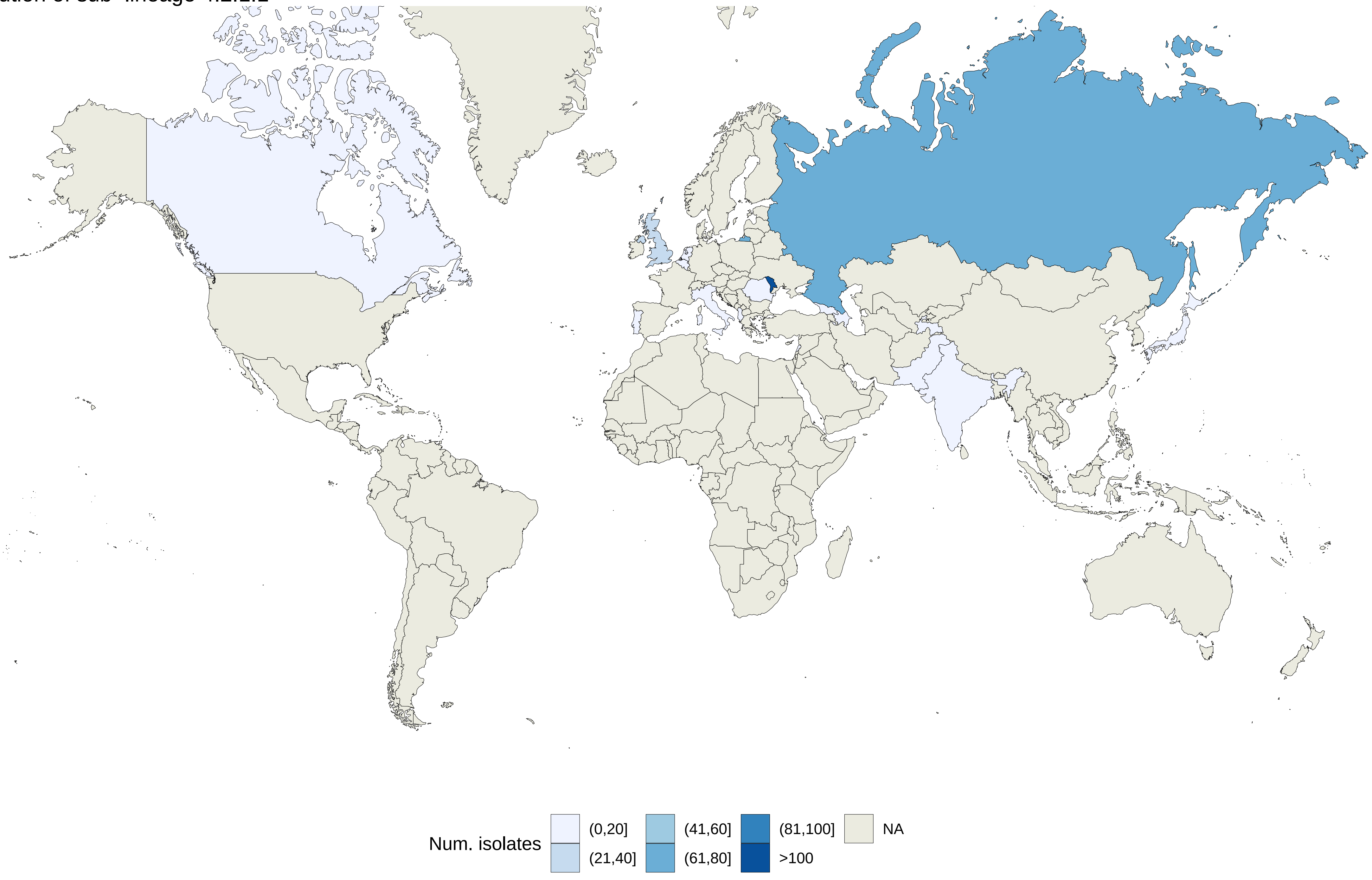

Distribution of sub−lineage 4.2.1.2

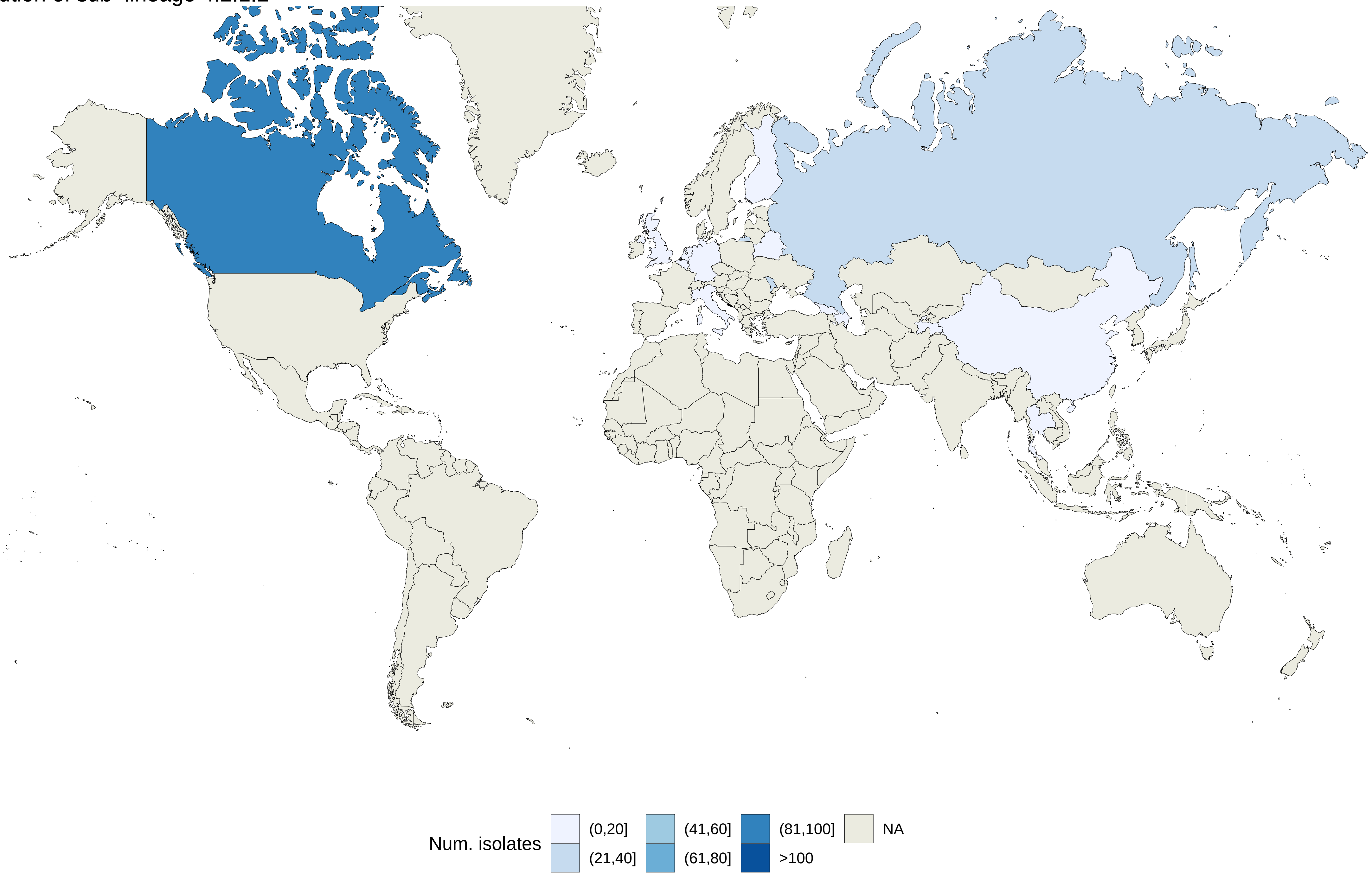

Distribution of sub−lineage 4.2.2

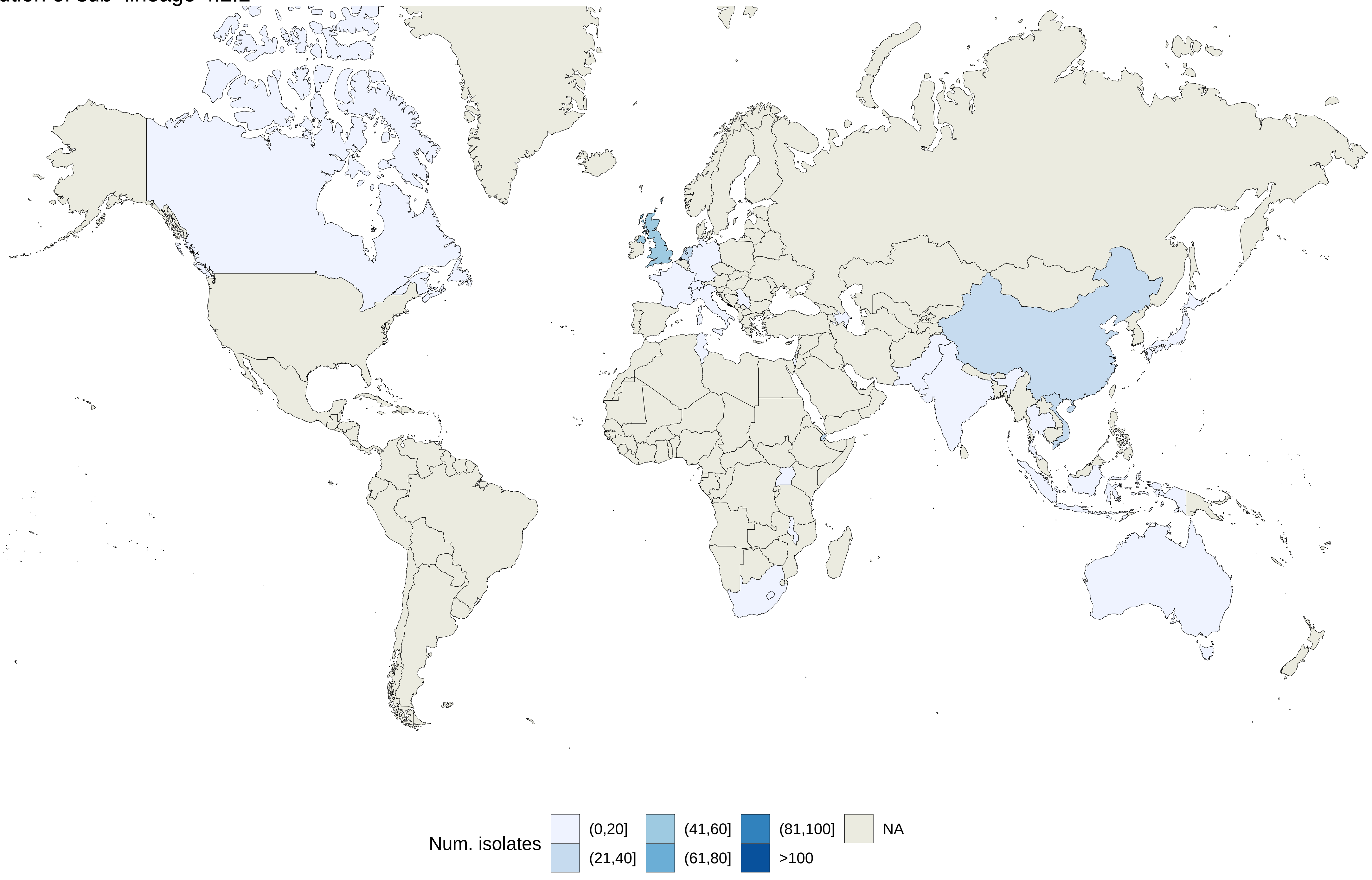

Distribution of sub−lineage 4.3.i1

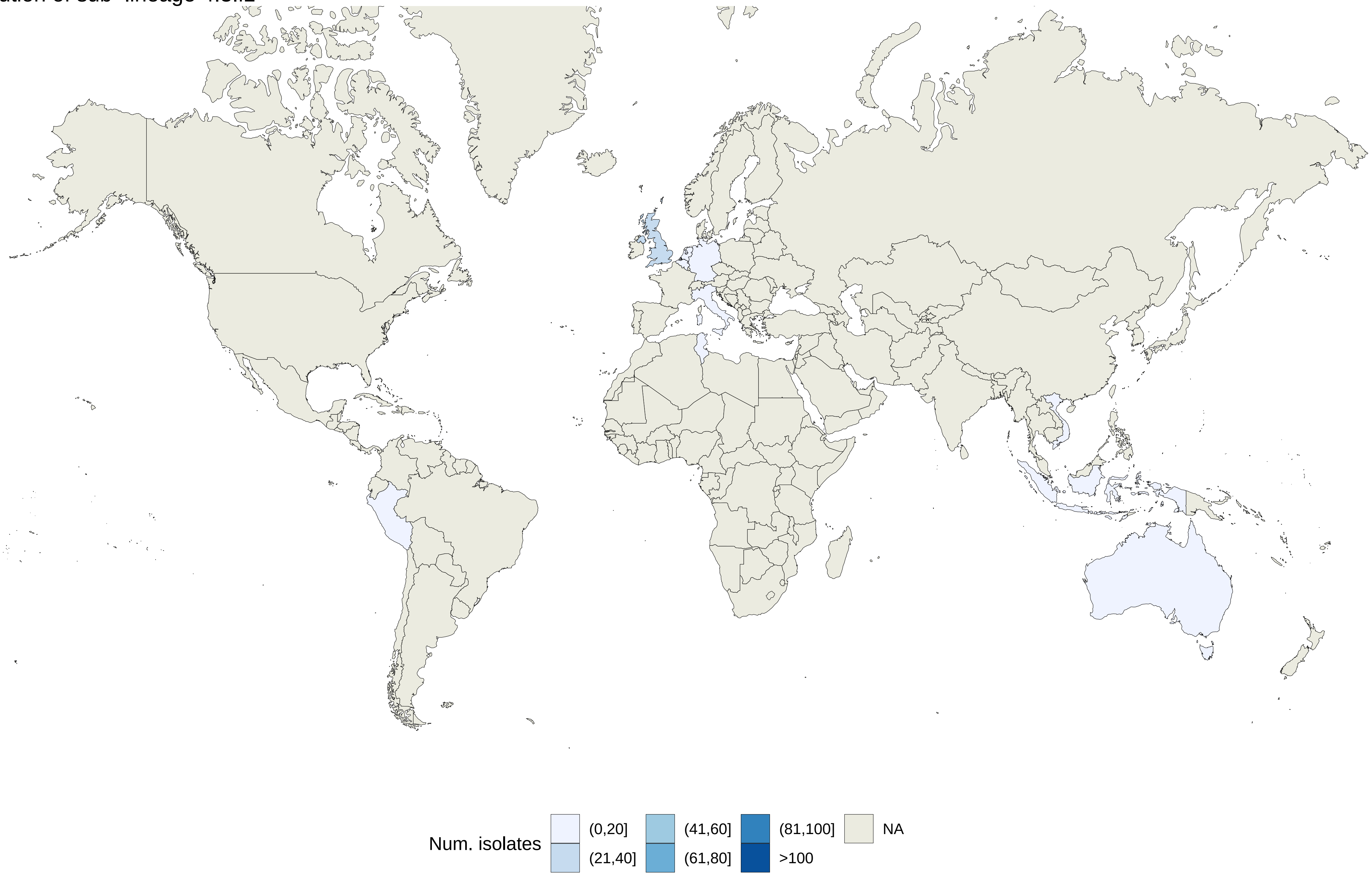

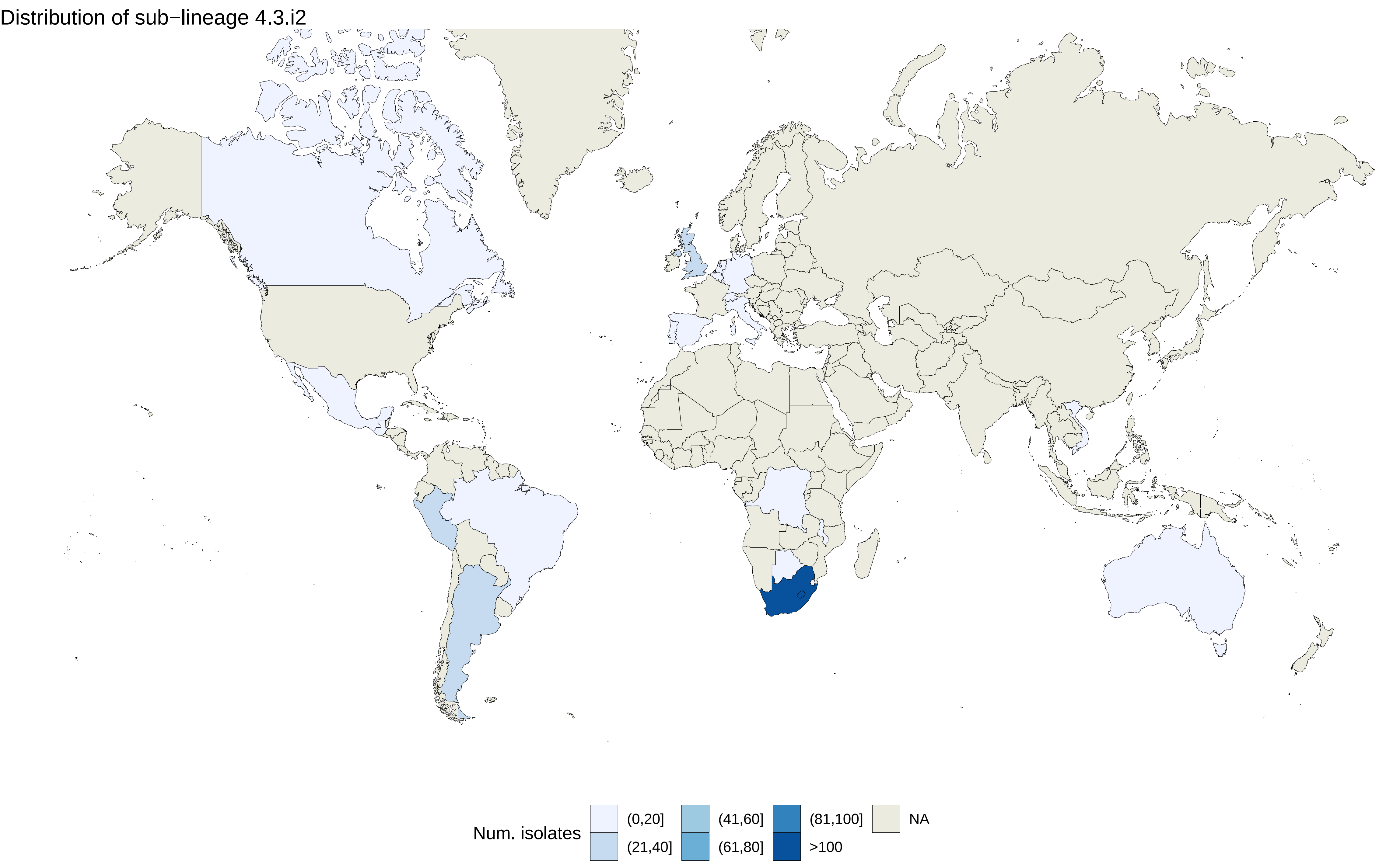
Distribution of sub−lineage 4.3.i2
Num. isolates
(0,20]
(21,40]
(41,60]
(61,80]
(81,100]
>100
NA

Distribution of sub−lineage 4.3.i3.1

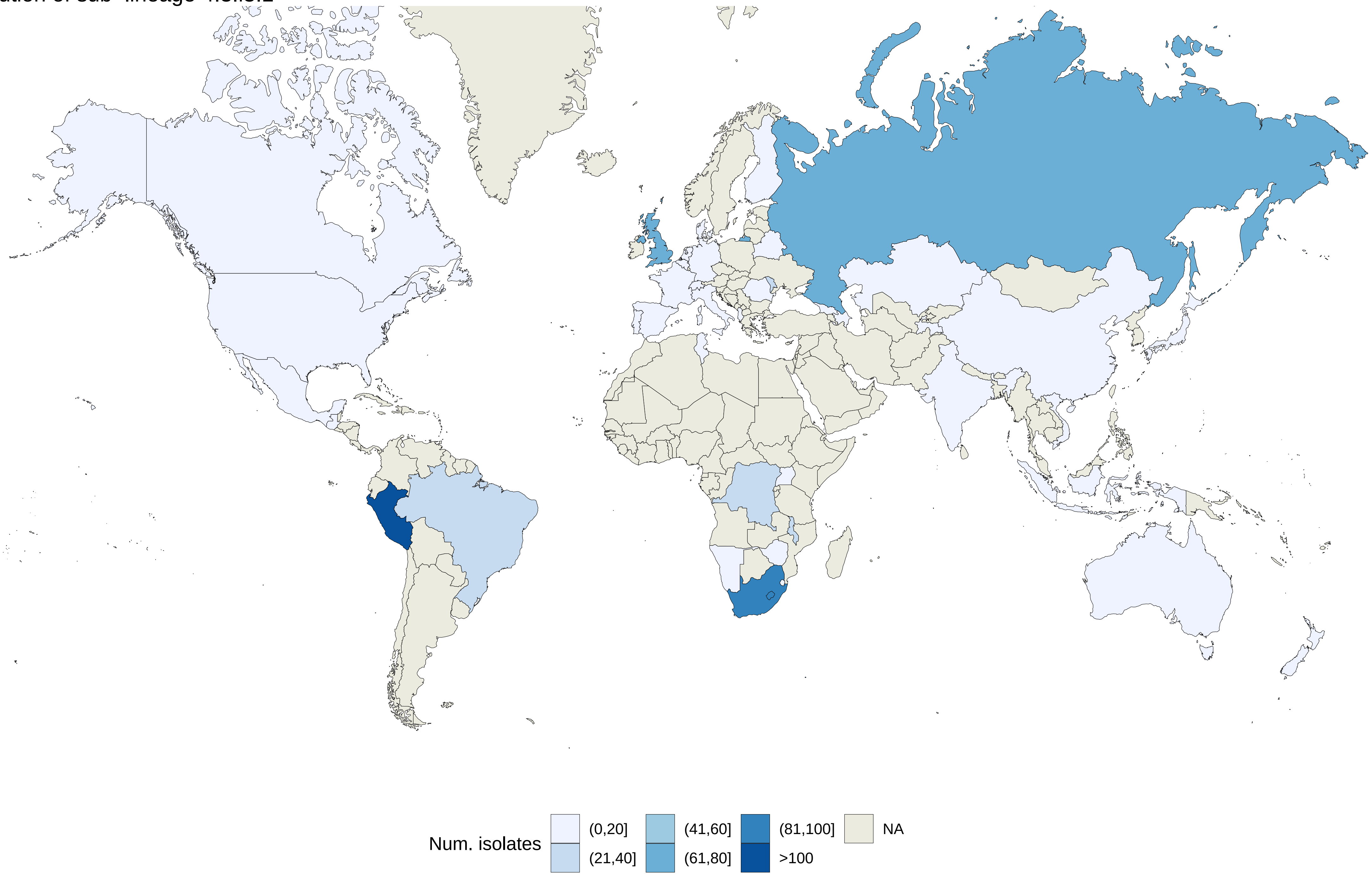

Distribution of sub−lineage 4.3.i3.2

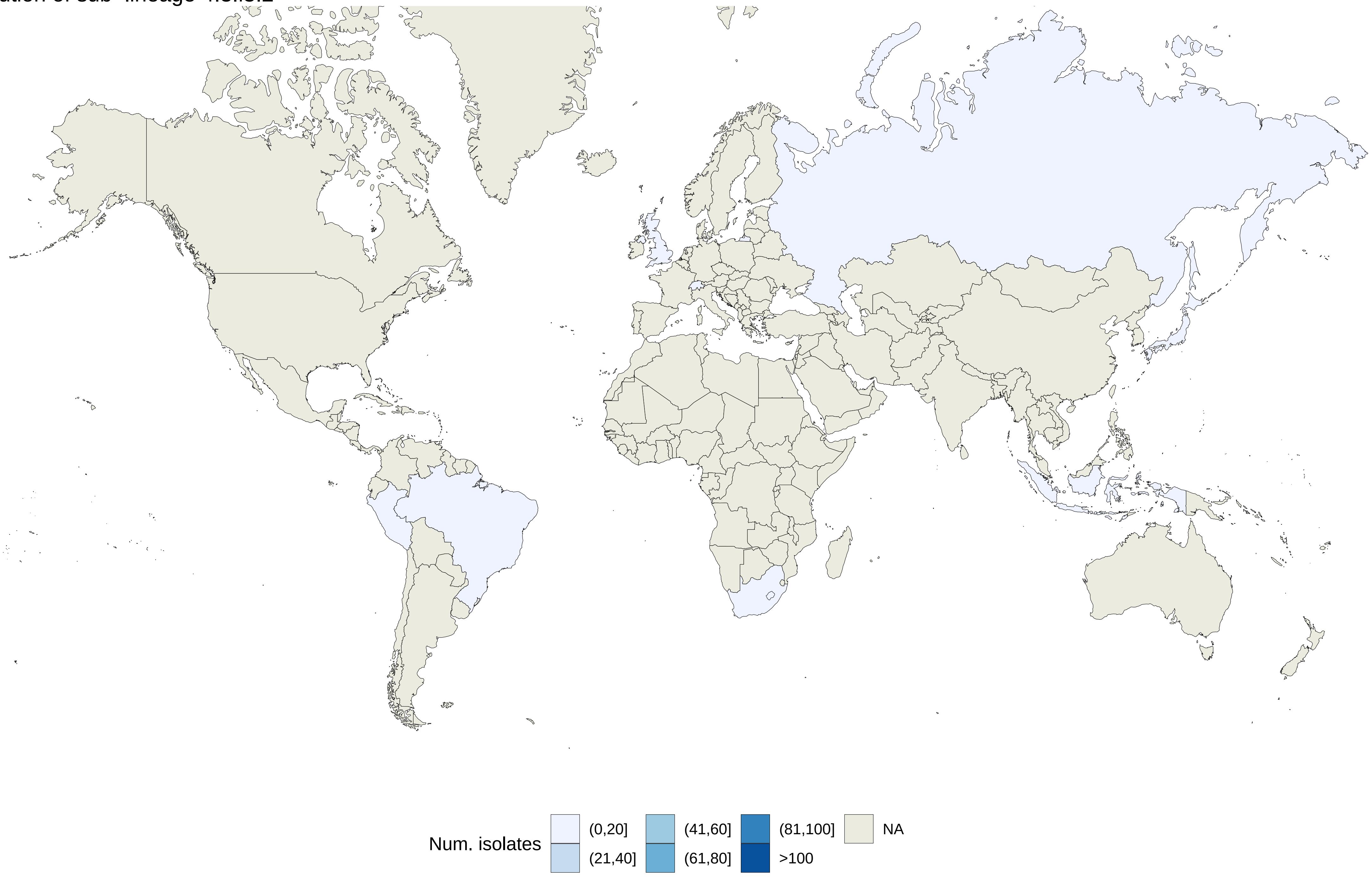

Distribution of sub−lineage 4.3.i4.1

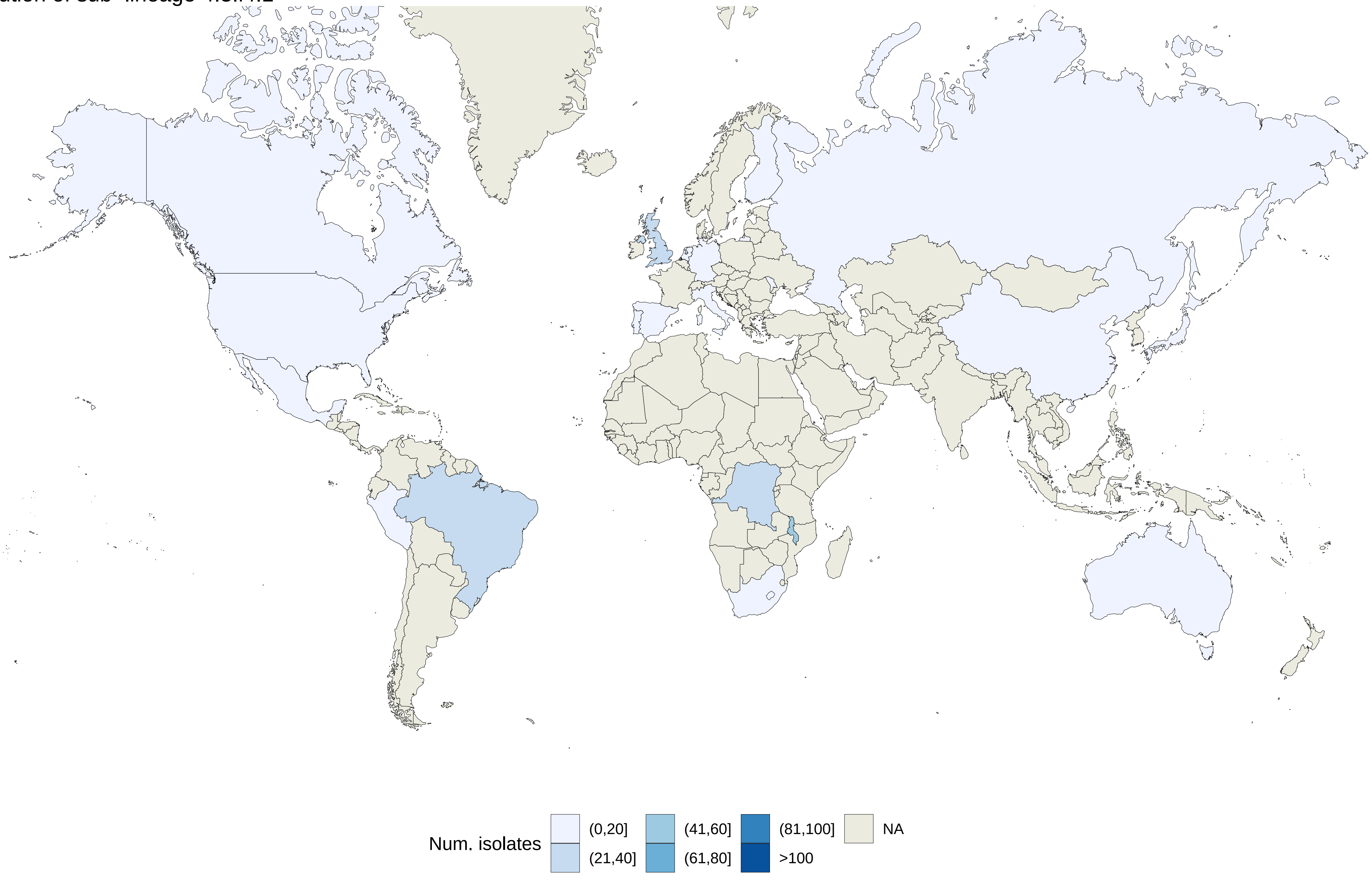

Distribution of sub−lineage 4.3.i4.2

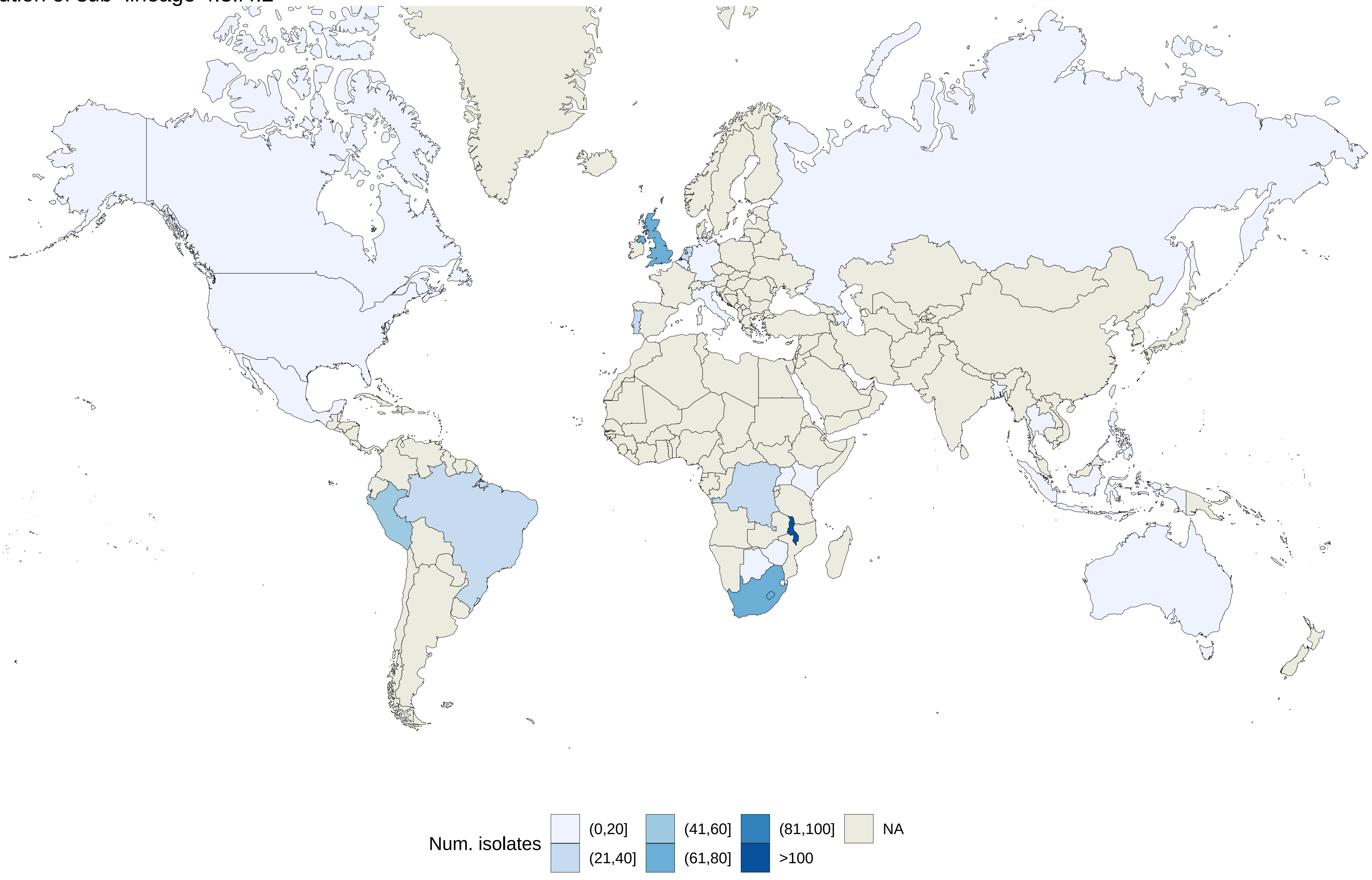

Distribution of sub−lineage 4.4.1.1

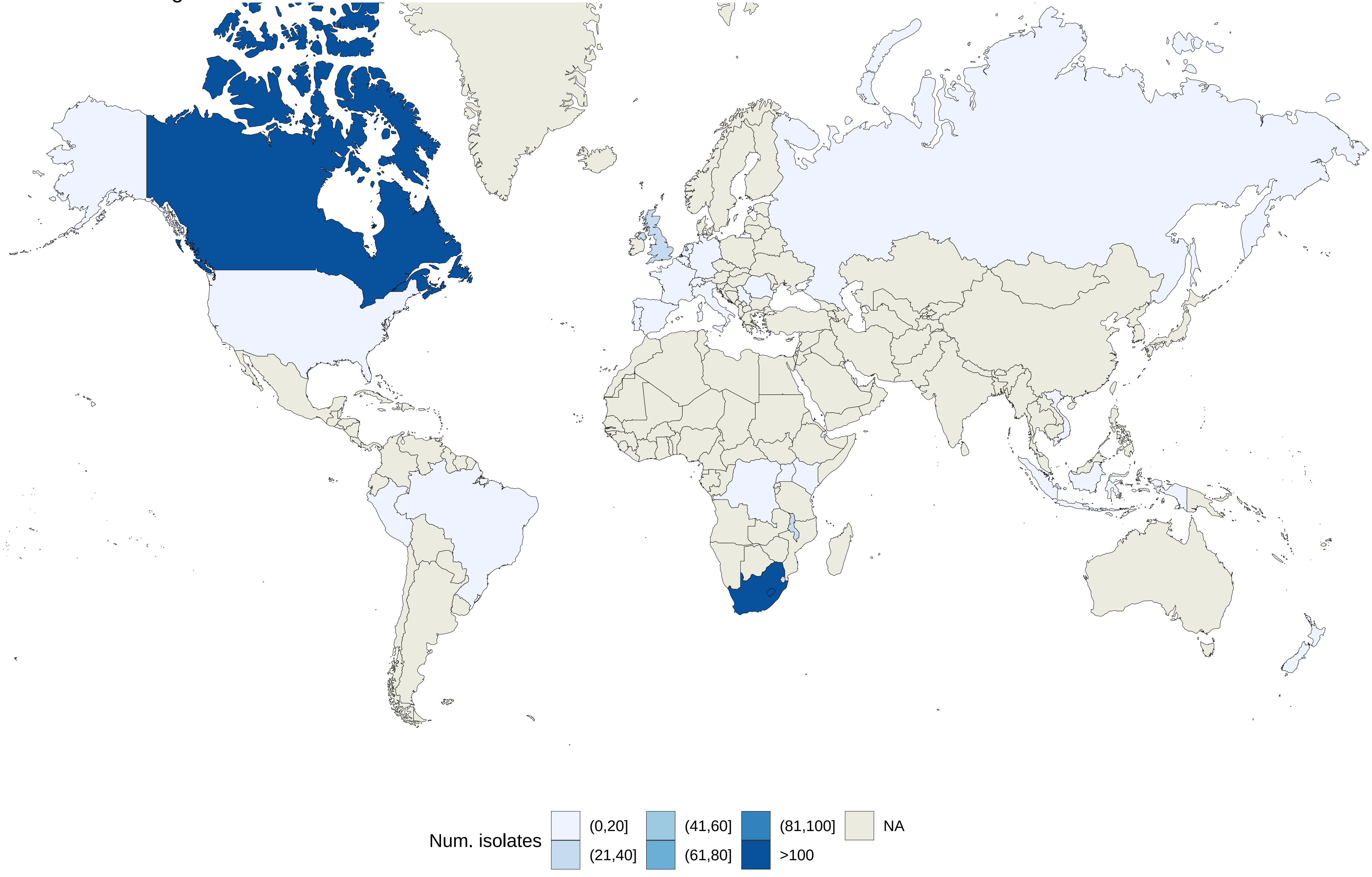

Distribution of sub−lineage 4.4.1.2

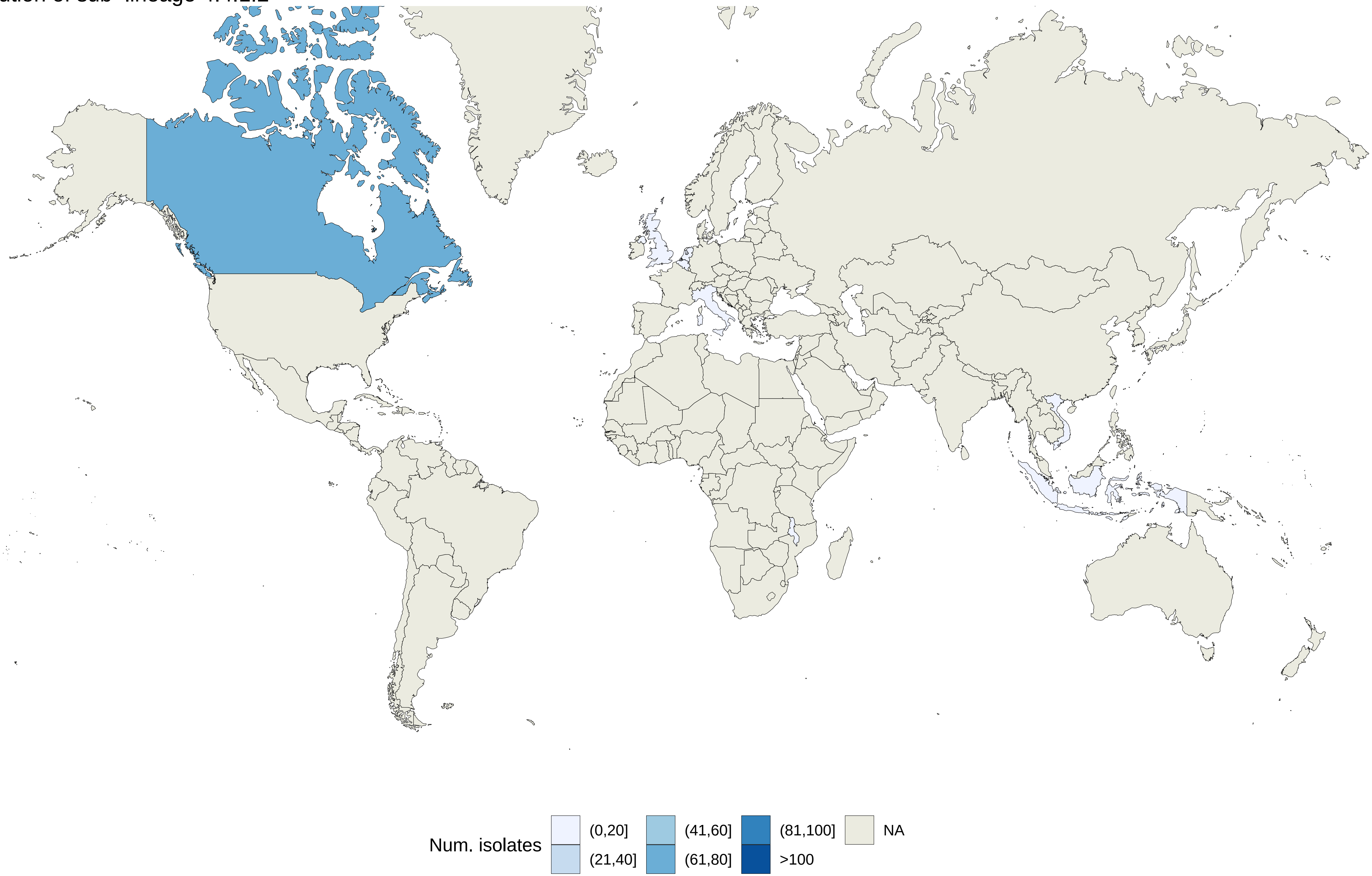

Distribution of sub−lineage 4.4.2

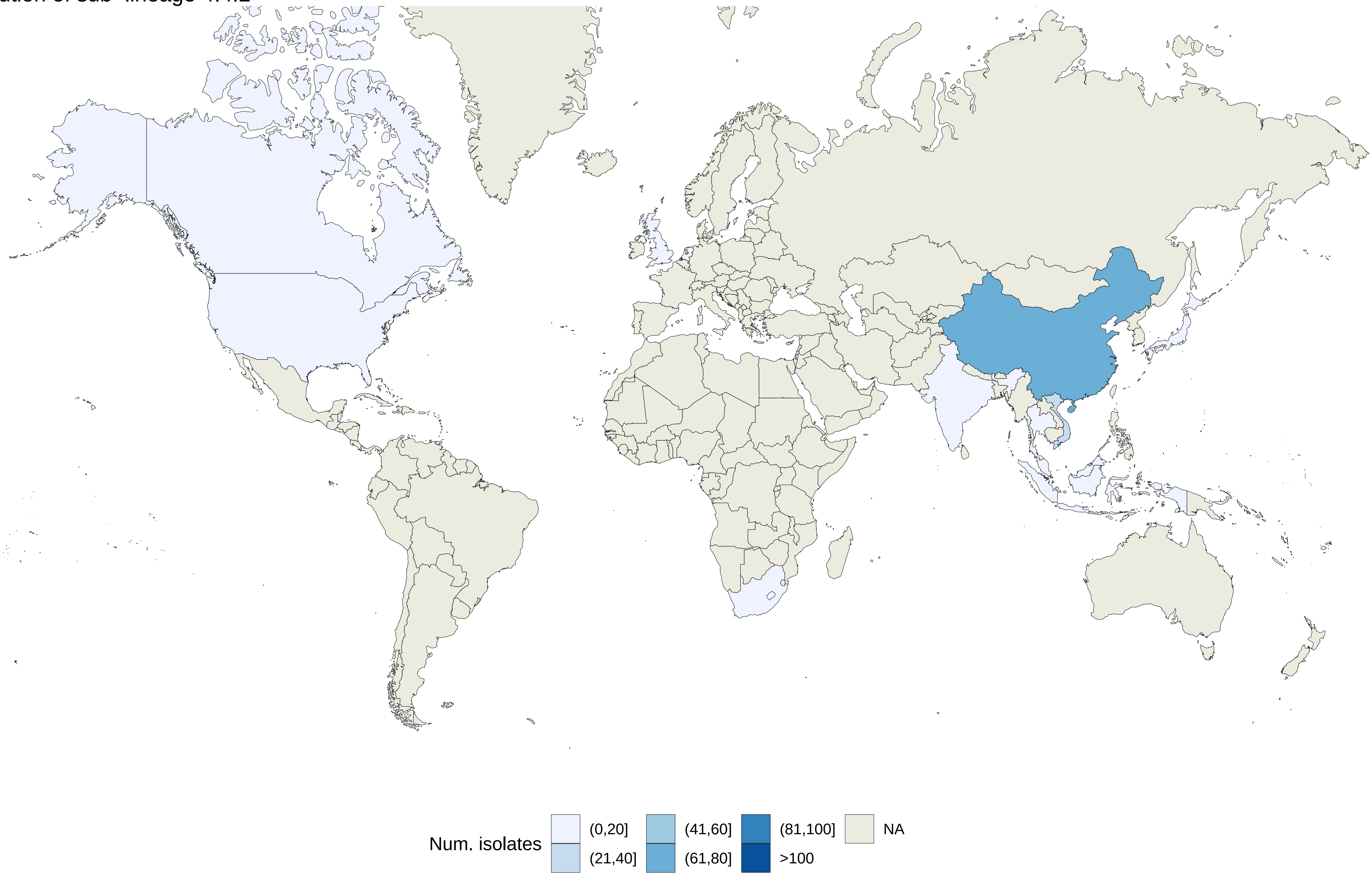

Distribution of sub−lineage 4.5

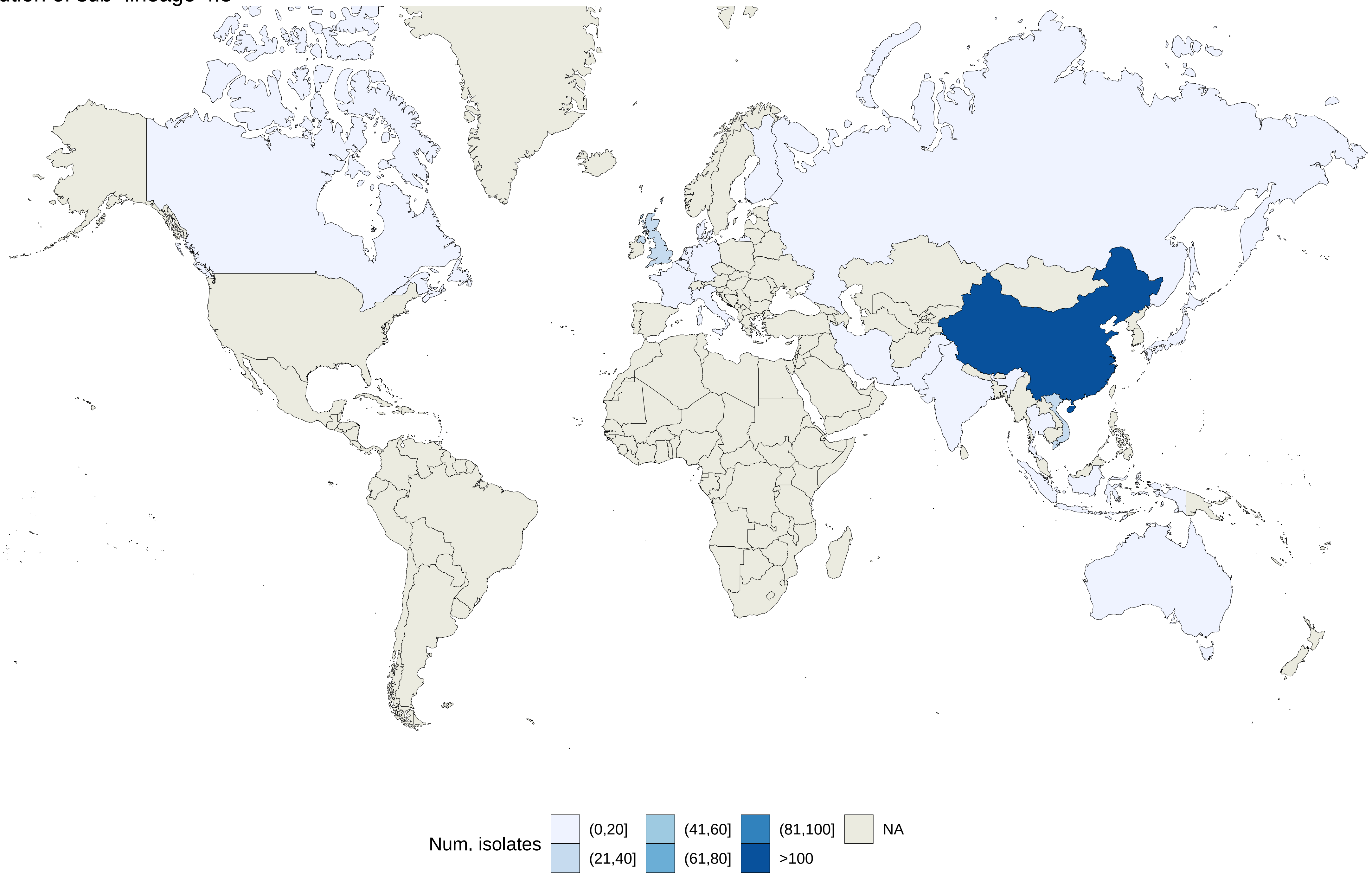

Distribution of sub−lineage 4.6.1.1.1.1.1

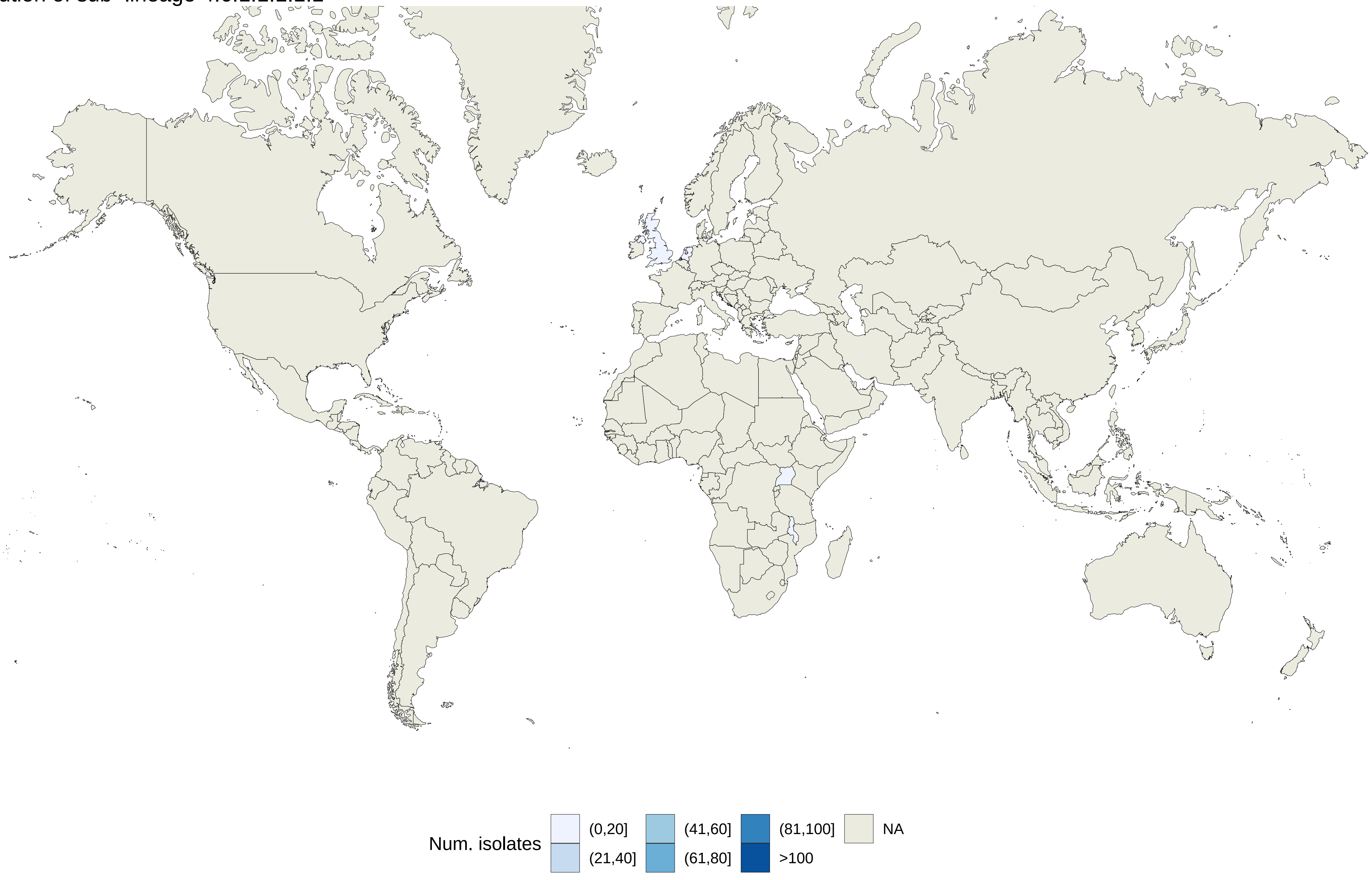

Distribution of sub−lineage 4.6.1.1.1.1.2

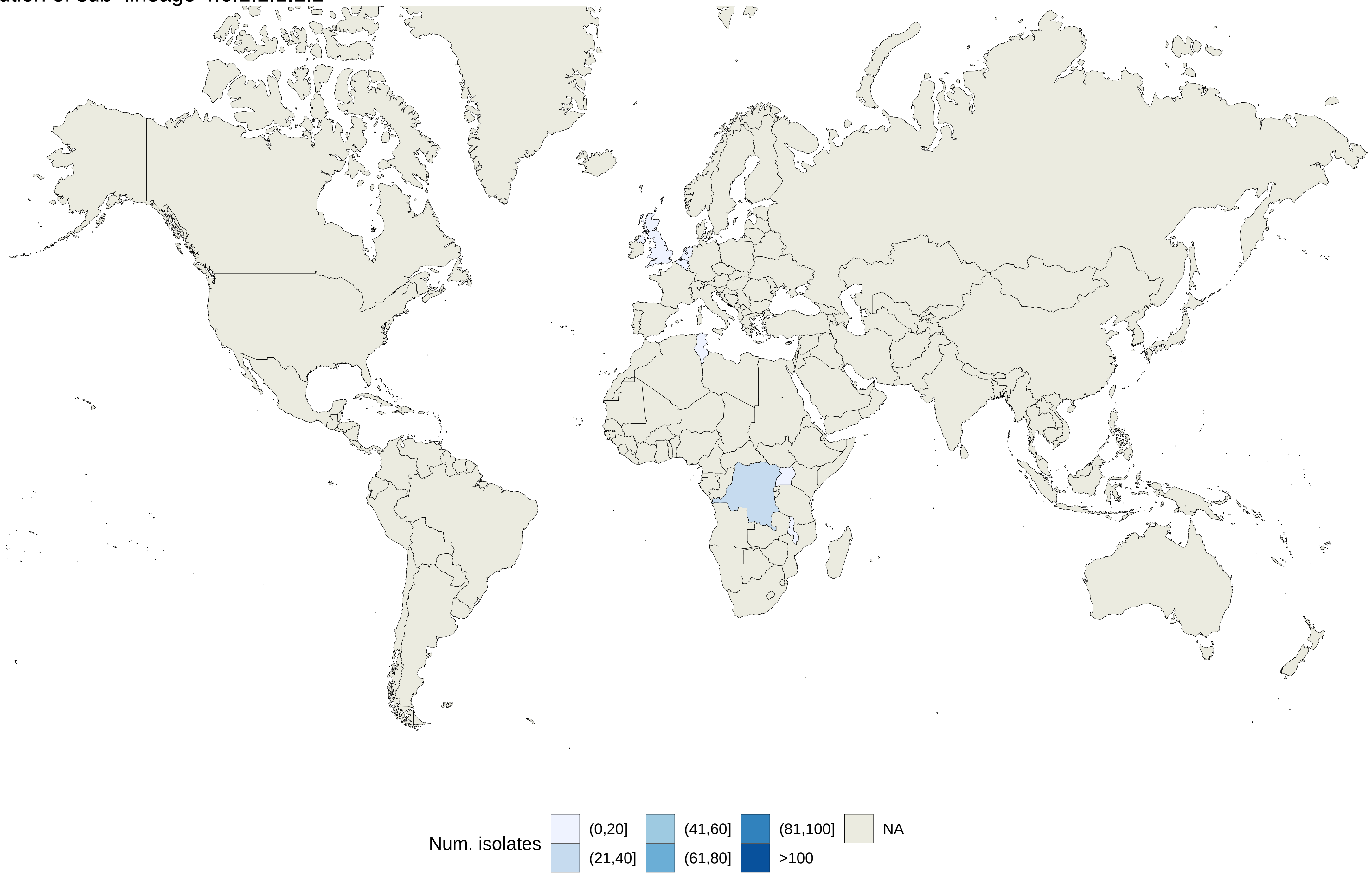

Distribution of sub−lineage 4.6.1.1.1.2

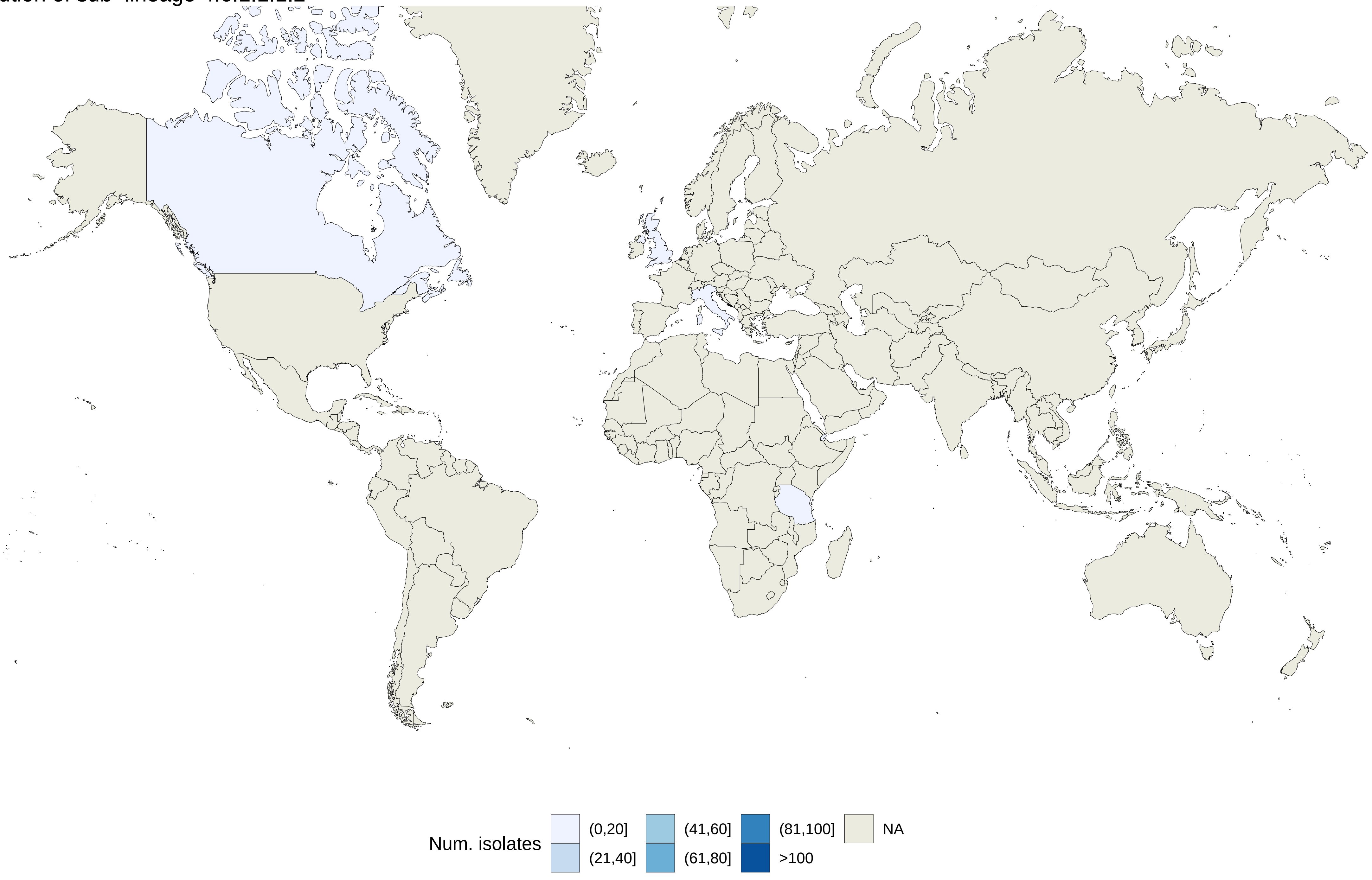

Distribution of sub−lineage 4.6.1.1.2

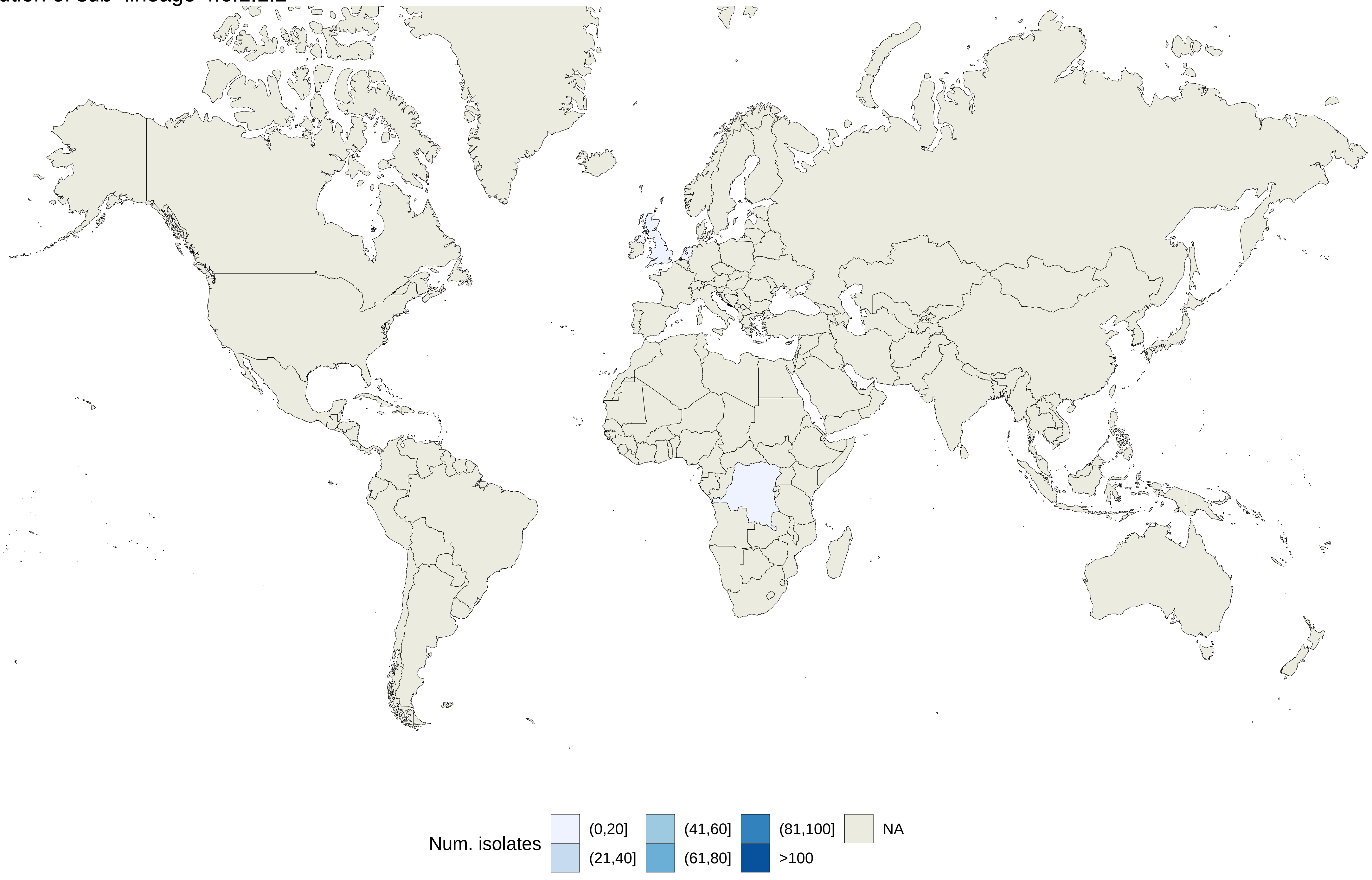

Distribution of sub−lineage 4.6.1.2

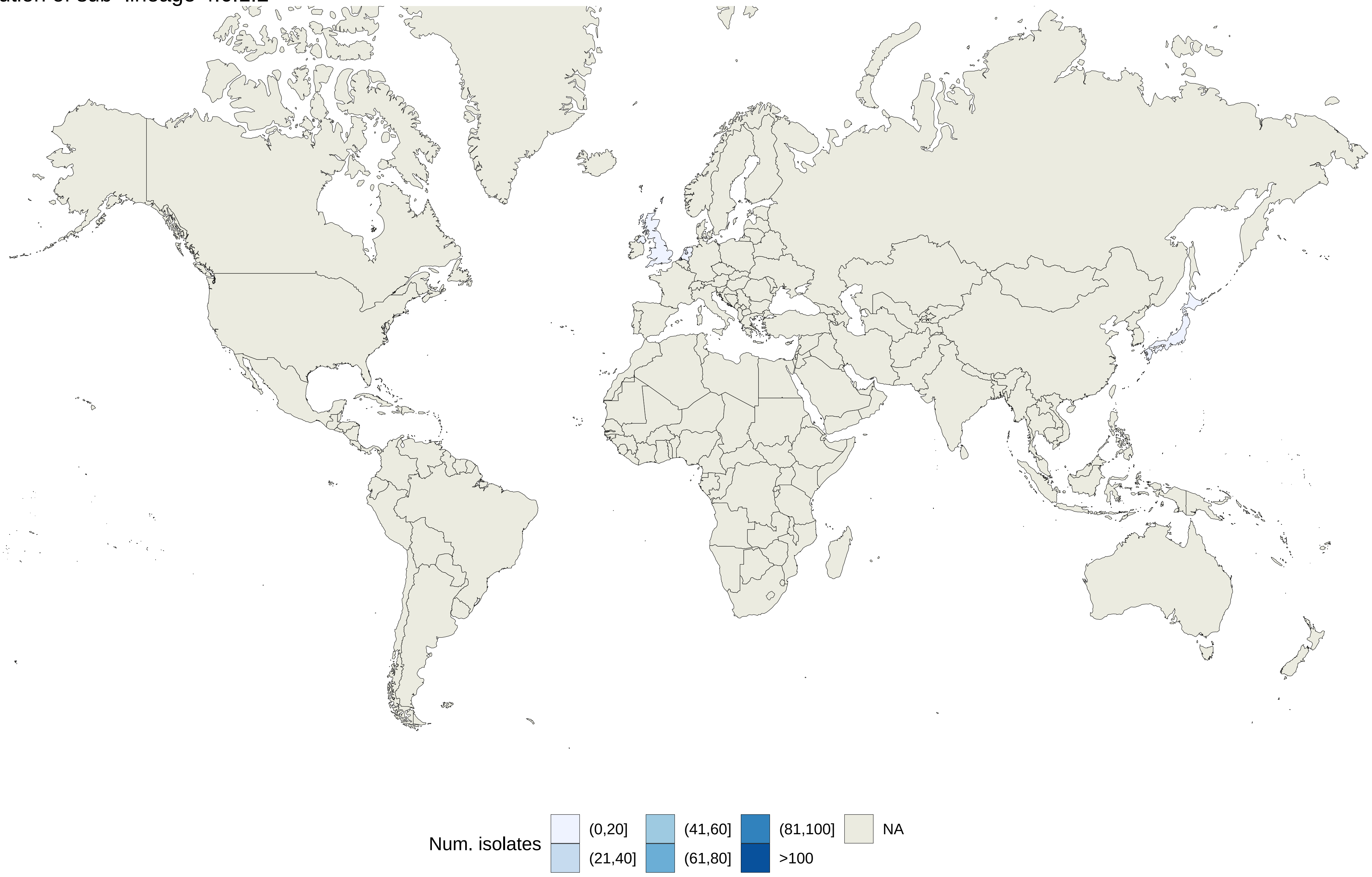

Distribution of sub−lineage 4.6.2.1.1.1.1

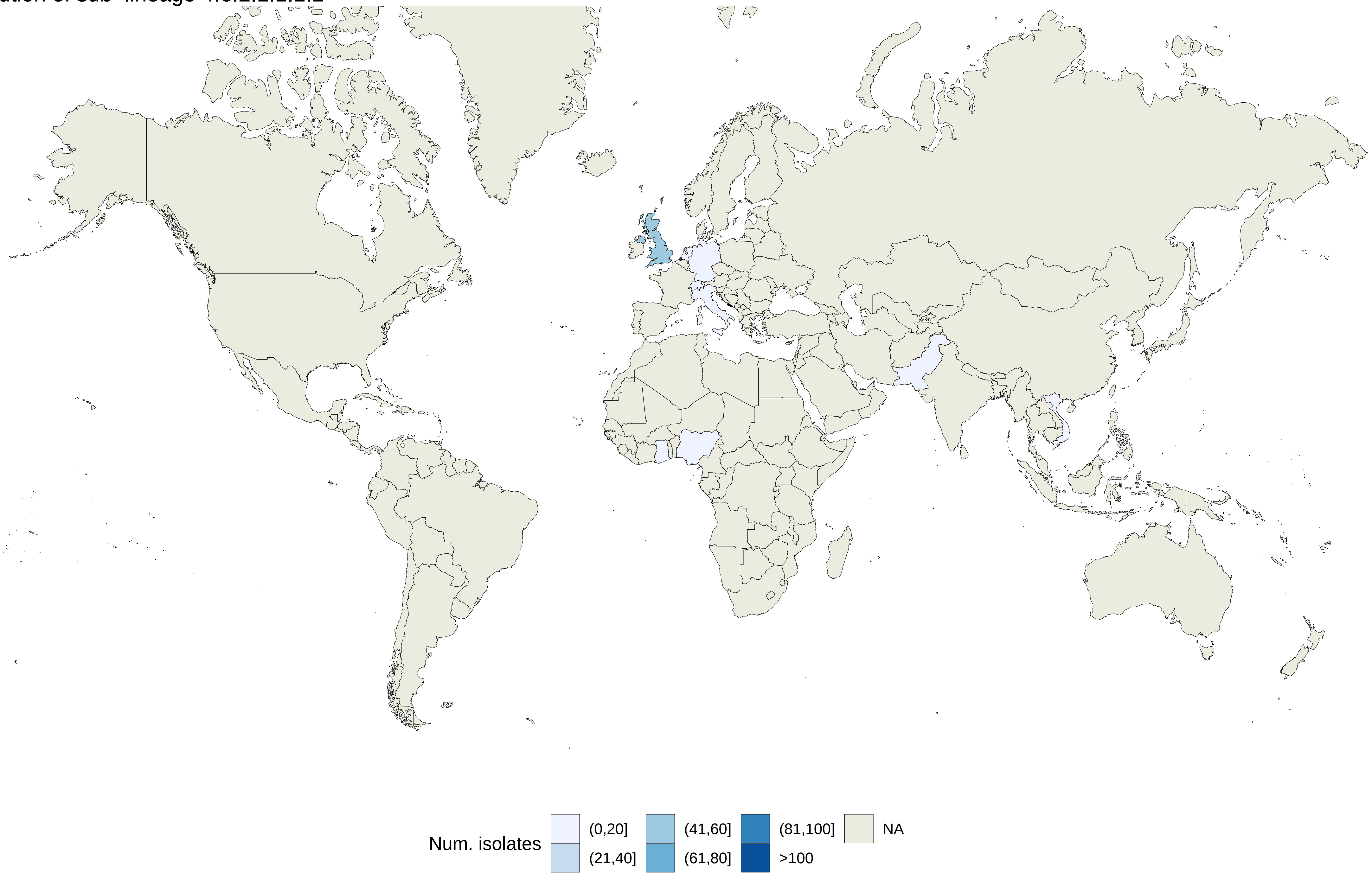

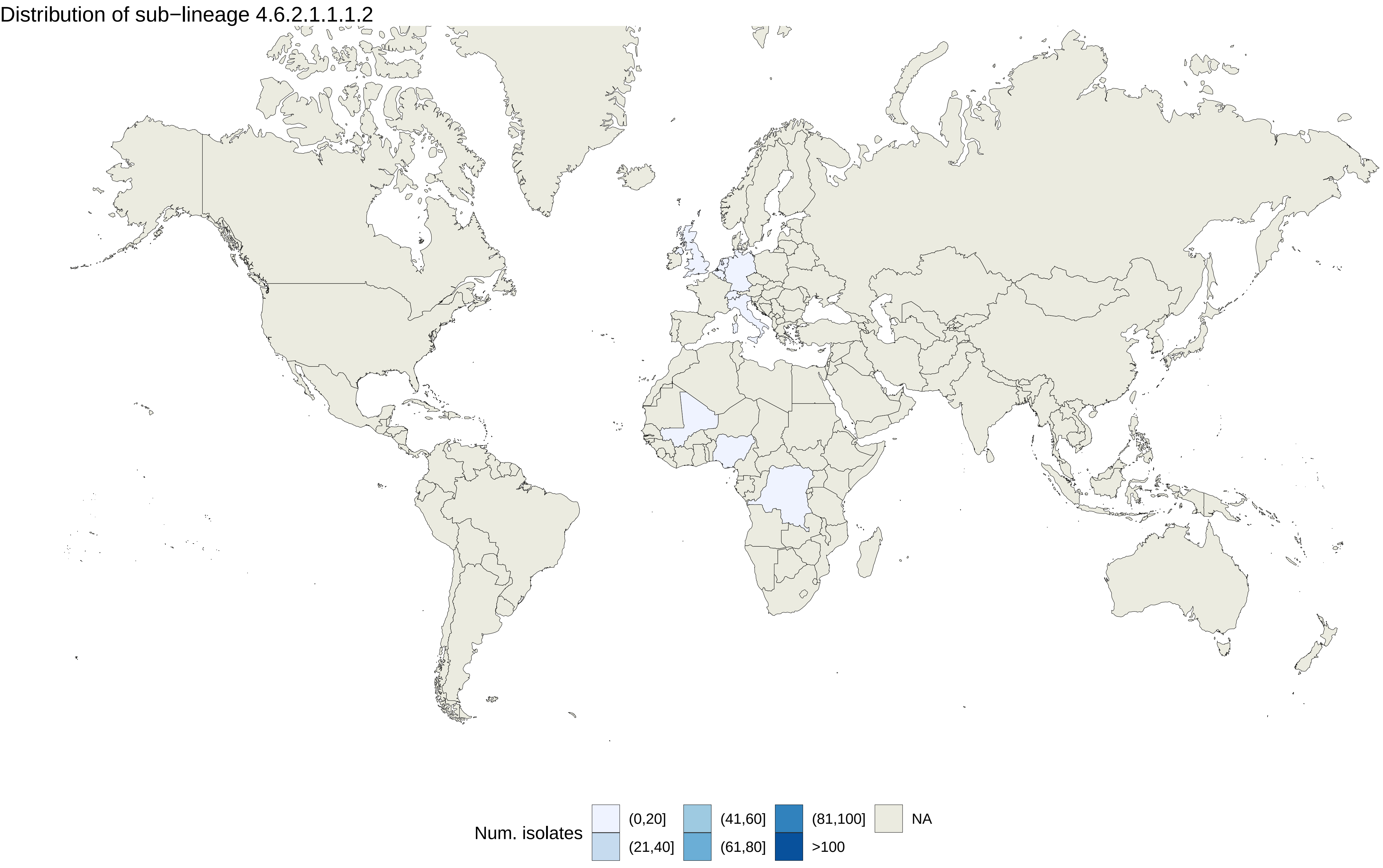
Distribution of sub−lineage 4.6.2.1.1.1.2
Num. isolates
(0,20]
(21,40]
(41,60]
(61,80]
(81,100]
>100
NA

Distribution of sub−lineage 4.6.2.1.1.2

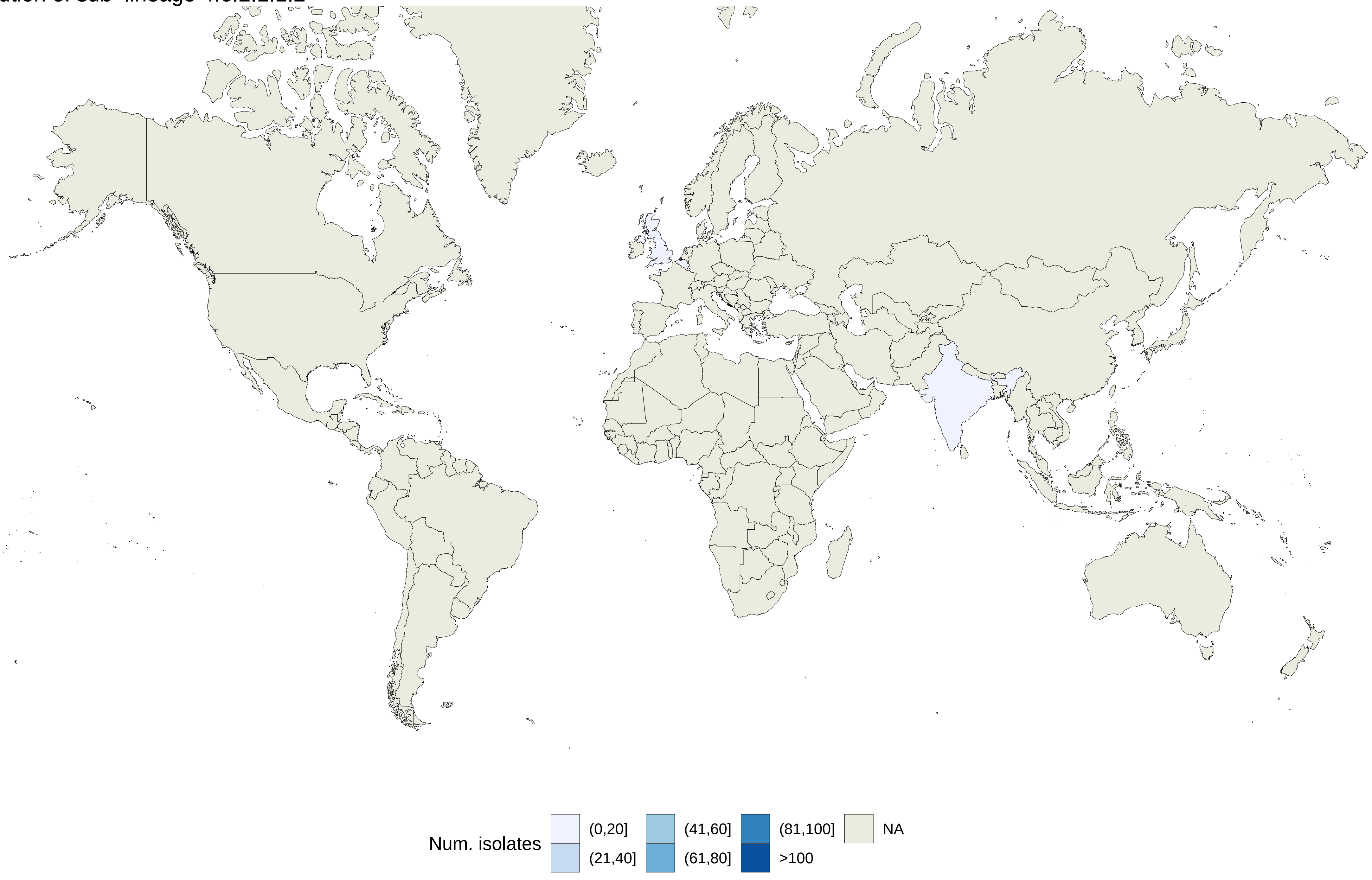

Distribution of sub−lineage 4.6.2.1.2

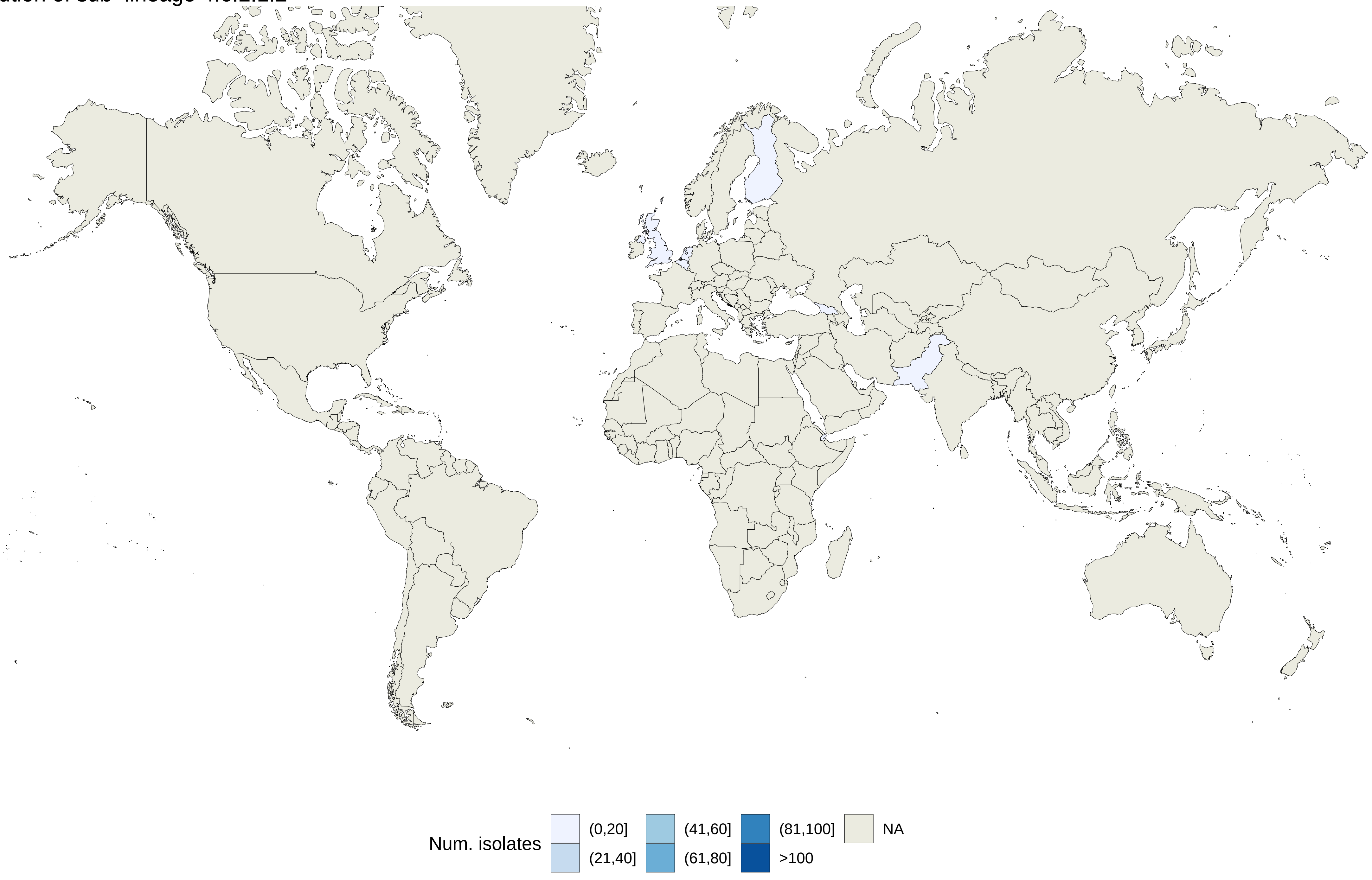

Distribution of sub−lineage 4.6.2.2

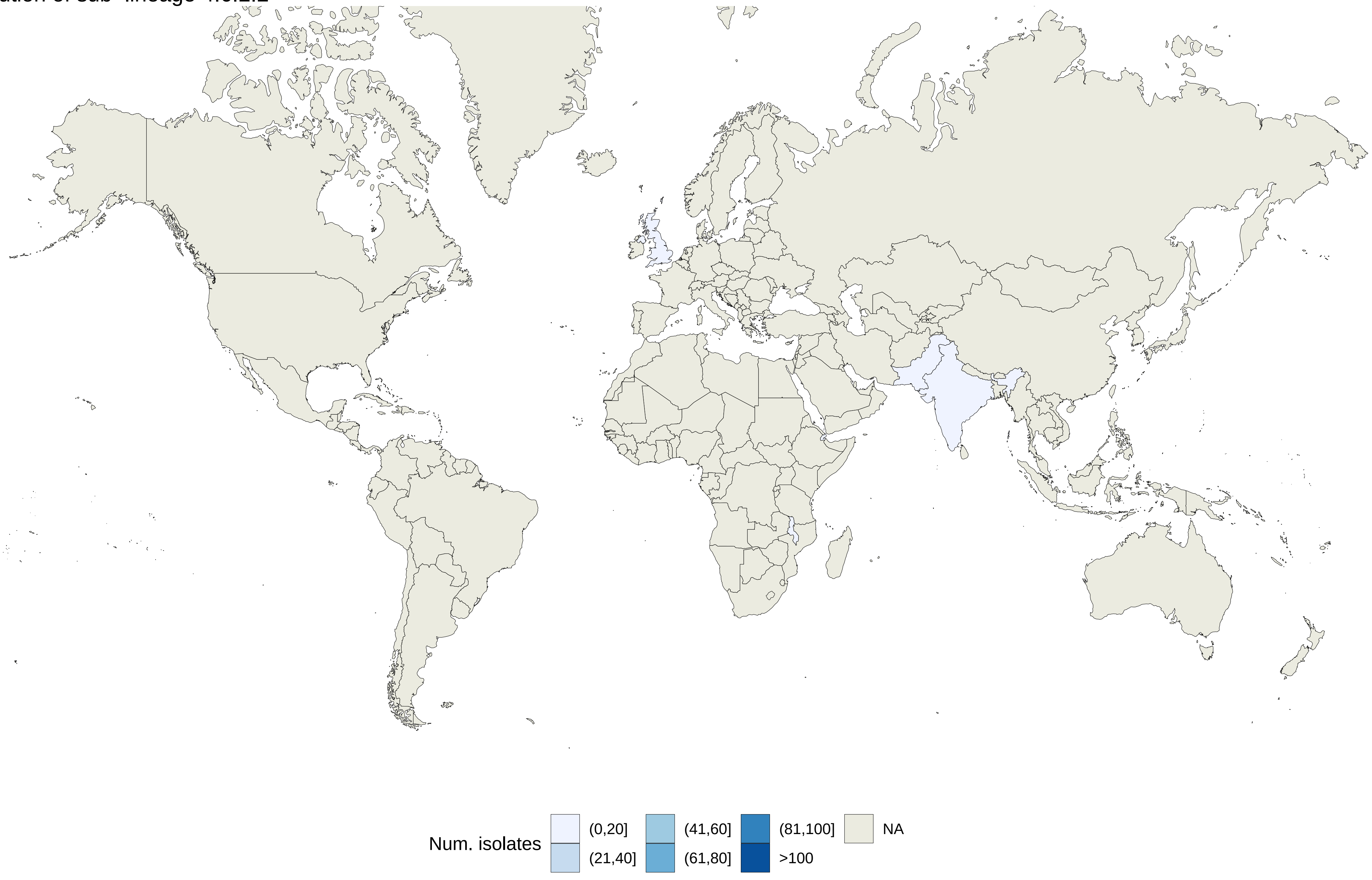

Supplement: Supplementary file 8 — Supplementary Data 5 [file 41467_2021_26248_MOESM8_ESM.zip › Supplementary_Data_5.pdf]
