# Supplementary material for: Population structure, biogeography and transmissibility of Mycobacterium tuberculosis
Source: Nat Commun. 2021 Oct 20;12:6099. doi: 10.1038/s41467-021-26248-1 (PMC8528816; doi:10.1038/s41467-021-26248-1)
Supplement: Supplementary file 12 — Reporting Summary [file 41467_2021_26248_MOESM12_ESM.pdf]

nature portfolio

Corresponding author(s): Maha Farhat
Luca Freschi

Last updated by author(s): Jul 29, 2021

Reporting Summary

Nature Portfolio wishes to improve the reproducibility of the work that we publish. This form provides structure for consistency and transparency in reporting. For further information on Nature Portfolio policies, see our Editorial Policies and the Editorial Policy Checklist.

Statistics

For all statistical analyses, confirm that the following items are present in the figure legend, table legend, main text, or Methods section.

n/a	Confirmed	
☐	☒	The exact sample size (*n*) for each experimental group/condition, given as a discrete number and unit of measurement
☒	☐	A statement on whether measurements were taken from distinct samples or whether the same sample was measured repeatedly
☐	☒	The statistical test(s) used AND whether they are one- or two-sided<br>*Only common tests should be described solely by name; describe more complex techniques in the Methods section.*
☒	☐	A description of all covariates tested
☐	☒	A description of any assumptions or corrections, such as tests of normality and adjustment for multiple comparisons
☐	☒	A full description of the statistical parameters including central tendency (e.g. means) or other basic estimates (e.g. regression coefficient) AND variation (e.g. standard deviation) or associated estimates of uncertainty (e.g. confidence intervals)
☐	☒	For null hypothesis testing, the test statistic (e.g. *F*, *t*, *r*) with confidence intervals, effect sizes, degrees of freedom and *P* value noted<br>*Give P values as exact values whenever suitable.*
☒	☐	For Bayesian analysis, information on the choice of priors and Markov chain Monte Carlo settings
☒	☐	For hierarchical and complex designs, identification of the appropriate level for tests and full reporting of outcomes
☐	☒	Estimates of effect sizes (e.g. Cohen's *d*, Pearson's *r*), indicating how they were calculated

Our web collection on statistics for biologists contains articles on many of the points above.

Software and code

Policy information about availability of computer code

Data collection	We used SRAtools version 2.9.1 to collect sequencing data from NCBI and code available at https://github.com/farhat-lab/resdata (DOI: https://zenodo.org/badge/latestdoi/136651308) to harmonize phenotypic resistance data.
Data analysis	We used the following software packages to analyse data (individually referenced in the methods section): PRINSEQ v0.20.4, Kraken v0.10.6, BWAmem v0.7.17, Picard v2.9.2, Samtools v1.9, Pilon v1.22, bcftools v1.9, vcf2phylip v1.5, iqtree v1.6.10, R v3.5.1, R popgenome v2.6.1, R ade4 v1.7-8, R ape v5.3, R phangorn v2.5.3, R vegan v2.5.6, R stats v3.5.1. New and custom software is available on github (DOIs: https://zenodo.org/badge/latestdoi/105924428, and 5097815)

For manuscripts utilizing custom algorithms or software that are central to the research but not yet described in published literature, software must be made available to editors and reviewers. We strongly encourage code deposition in a community repository (e.g. GitHub). See the Nature Portfolio guidelines for submitting code & software for further information.

Data

Policy information about availability of data

All manuscripts must include a data availability statement. This statement should provide the following information, where applicable:

- Accession codes, unique identifiers, or web links for publicly available datasets
- A description of any restrictions on data availability
- For clinical datasets or third party data, please ensure that the statement adheres to our policy

A summary table of the first dataset of 11,349 public isolates including drug resistance phenotypes and the scripts used to generate it from source databases are available at https://github/farhat-lab/resdata (v1.0). Isolates were filtered according to the criteria described in the methods resulting in a dataset of 9,584 isolates (described in Suppl. File 1). To determine the geographic distribution of Mtb sub-lineages identified in this data, we used a second dataset of 17,431 isolates

(accession numbers are provided in Suppl. File 4). Lastly, to validate the geographic distribution of sub-lineages we used a third dataset of 3,791 Mtb isolates systematically samples from five countries and three continents (Azerbaijan, Bangladesh, Pakistan, South Africa and Ukraine and accession numbers are detailed in Suppl. File 6). Source data is provided online to regenerate main text and supplementary figures. Any additional data are available from the corresponding authors upon reasonable request.

Field-specific reporting

Please select the one below that is the best fit for your research. If you are not sure, read the appropriate sections before making your selection.

☒ Life sciences ☐ Behavioural & social sciences ☐ Ecological, evolutionary & environmental sciences

For a reference copy of the document with all sections, see nature.com/documents/nr-reporting-summary-flat.pdf

Life sciences study design

All studies must disclose on these points even when the disclosure is negative.

Sample size

For the dataset (1, see data collection) we curated all the main works available in the literature at the time in order to come out with our set of isolates with AMR phenotypes.
For the dataset (2), we downloaded isolate metadata present on NCBI to get the largest dataset of isolates publicly available for which the country of isolation was available.
For dataset (3), we relied on the only available study where Mtb isolates were sampled systematically in 5 countries to be representative of the Mtb patient population.

Data exclusions

We excluded isolates with signs of contamination (< 90% of the reads were assigned to Mtb by Kraken), insufficient coverage (positions with a coverage of at least 10x are less than 95% of the genome) or potential cases of mixed isolates (F2 lineage-mixture metric > 0.5).

Replication

We used the resistant isolates to replicate sub-lineages initially defined with susceptible isolates only. Using this approach we reproduced the majority of sub-lineages except for a few cases that were related to lack of adequate representation in the resistant group and not due to misclassification of the lineages as detailed in the manuscript. In a second replication effort, we confirmed the presence of sub-lineages in an isolate dataset systematically sampled to represent TB in a particular country.
To replicate differences in transmissibility between the four major Mtb lineages, we used three complementary measures that confirmed the reported order of transmissibility. Finally we confirmed that differences in transmissibility held in different datasets.

Randomization

No randomization was performed on phenotypes or interventions in this study as the focus was purely on classification of population structure. We did obtain bootstrap support on phylogenies and this involved data resampling, but not randomization per se.

Blinding

No blinding was performed in this study due to the focus on population structure determination which is a form of unsupervised learning, there were no labels, phenotypes, or outcomes to blind.

Reporting for specific materials, systems and methods

We require information from authors about some types of materials, experimental systems and methods used in many studies. Here, indicate whether each material, system or method listed is relevant to your study. If you are not sure if a list item applies to your research, read the appropriate section before selecting a response.

Materials & experimental systems

n/a	Involved in the study	
☒	☐	Antibodies
☒	☐	Eukaryotic cell lines
☒	☐	Palaeontology and archaeology
☒	☐	Animals and other organisms
☒	☐	Human research participants
☒	☐	Clinical data
☒	☐	Dual use research of concern

Methods

n/a	Involved in the study	
☒	☐	ChIP-seq
☒	☐	Flow cytometry
☒	☐	MRI-based neuroimaging
