# Supplementary material for: Population structure, biogeography and transmissibility of Mycobacterium tuberculosis
Source: Nat Commun. 2021 Oct 20;12:6099. doi: 10.1038/s41467-021-26248-1 (PMC8528816; doi:10.1038/s41467-021-26248-1)
Supplement: Supplementary file 13 — Source Data [file 41467_2021_26248_MOESM13_ESM.zip › source_data/Source Data README.docx]

## Source Data README

This README file allows finding the Source Data files corresponding to each of the figures in the main manuscript or the Supplementary Information.

| **Figure** | **File(s)** | **Sheet (.xlsx file)** | **Description** |
| --- | --- | --- | --- |
| Fig. 1 | tree_lineage1_pan-susceptible.nex | - | Phylogenetic tree for lineage 1 pan-susceptible isolates. |
| Fig. 2 | tree_lineage2_pan-susceptible.nex | - | Phylogenetic tree for lineage 2 pan-susceptible isolates. |
| Fig. 3 | tree_lineage3_pan-susceptible.nex | - | Phylogenetic tree for lineage 3 pan-susceptible isolates. |
| Fig. 4 | tree_lineage4_pan-susceptible.nex | - | Phylogenetic tree for lineage 4 pan-susceptible isolates. |
| Fig. 5 | Source Data XLSX.xlsx | s_idx_num_isol_cont, s_idx_gen_spec | Table listing, for each sub-lineage, the Simpson index, the number of available isolates and in how many continents were found (s_idx_num_isol_cont); Simpson index for generalist and specialist sub-lineages described by Stucki et al. (s_idx_gen_spec) |
| Fig. 6 | Source Data XLSX.xlsx | geo_ncbi_L1_filter20 | Estimated proportion of 1.1.3.i1 lineage in countries with sub-lineage data. |
| Fig. 7 | Source Data XLSX.xlsx | geo_ncbi_L1_filter20 | Estimated proportion of 1.1.1.1 lineage in countries with sub-lineage data. |
| Fig. 8 | Source Data XLSX.xlsx | geo_ncbi_L1_filter20 | Estimated proportion of 1.1.2 lineage in countries with sub-lineage data. |
| Fig. 9 | tree_all_isolates_curated_phenotypes_dataset.treefile, Supplementary Data 1 | - | Phylogenetic tree for all the isolates of the dataset with phenotypic resistance data (tree_all_isolates_curated_phenotypes_dataset.treefile, it contains the values of the terminal branch lengths); Data for tree annotation (Supplementary Data 1) |
| Suppl. Fig. 1 | Source Data XLSX.xlsx | pheno_res_lin_breakdown | Table listing isolate ID, phenotype (susceptible: S, resistant: R) and main lineage for the dataset with phenotypic resistance data |
| Suppl. Fig. 2 | tree_lineage1_pan-susceptible.nex, Supplementary Data 2 | - | Phylogenetic tree for lineage 1 pan-susceptible isolates included in the dataset with phenotypic resistance data (tree_lineage1_pan-susceptible.nex); Data for tree annotation including lineage calls according to different lineage schemes (Supplementary Data 2) |
| Suppl. Fig. 3 | tree_lineage1_pan-susceptible.nex, Supplementary Data 2 | - | Phylogenetic tree for the lineage 1 pan-susceptible isolates included in the dataset with phenotypic resistance data (tree_lineage1_pan-susceptible.nex); Data for tree annotation including lineage calls according to different lineage schemes (Supplementary Data 2) |
| Suppl. Fig. 4 | tree_lineage2_pan-susceptible.nex, Supplementary Data 2 | - | Phylogenetic tree for the lineage 2 pan-susceptible isolates included in the dataset with phenotypic resistance data (tree_lineage2_pan-susceptible.nex); Data for tree annotation including lineage calls according to different lineage schemes (Supplementary Data 2) |
| Suppl. Fig. 5 | tree_lineage3_pan-susceptible.nex, Supplementary Data 2 | - | Phylogenetic tree for the lineage 3 pan-susceptible isolates included in the dataset with phenotypic resistance data (tree_lineage3_pan-susceptible.nex); Data for tree annotation including lineage calls according to different lineage schemes (Supplementary Data 2) |
| Suppl. Fig. 6 | tree_lineage4_pan-susceptible.nex, Supplementary Data 2 | - | Phylogenetic tree for the lineage 4 pan-susceptible isolates included in the dataset with phenotypic resistance data (tree_lineage4_pan-susceptible.nex); Data for tree annotation including lineage calls according to different lineage schemes (Supplementary Data 2) |
| Suppl. Fig. 7 | tree_lineage1_resistant.treefile | - | Phylogenetic tree for the lineage 1 resistant isolates included in the dataset with phenotypic resistance data (tree_lineage1_resistant.treefile) |
| Suppl. Fig. 8 | tree_lineage2_resistant.treefile | - | Phylogenetic tree for the lineage 2 resistant isolates included in the dataset with phenotypic resistance data (tree_lineage2_resistant.treefile) |
| Suppl. Fig. 9 | tree_lineage3_resistant.treefile | - | Phylogenetic tree for the lineage 3 resistant isolates included in the dataset with phenotypic resistance data (tree_lineage3_resistant.treefile) |
| Suppl. Fig. 10 | tree_lineage4_resistant.treefile | - | Phylogenetic tree for the lineage 4 resistant isolates included in the dataset with phenotypic resistance data (tree_lineage4_resistant.treefile) |
| Suppl. Fig. 11 | Supplementary Data 4 |  | Number of isolates per country of isolation (NCBI dataset / biogeography) |
| Suppl. Fig. 12 | Source Data XLSX.xlsx | s_idx_num_isol_cont | Table listing, for each sub-lineage, the Simpson index, the number of available isolates and in how many continents were found |
| Suppl. Fig. 13 | snp_distances_isolates_sublineages.tsv | snp_dist_isol_sublinages | Pairwise SNS distances of the isolates belonging to sub-lineages / internal groups that had Simpson diversity index < 0.28 |
| Suppl. Fig. 14 | Source Data XLSX.xlsx | s_idx_num_isol_cont | Table listing, for each sub-lineage, the Simpson index and the number of available isolates |
| Suppl. Fig. 15 | Supplementary Data 6 | - | Supplementary Data 6 provides isolate IDs, lineage calls and country of isolation |
| Suppl. Fig. 16 | Supplementary Data 6 | - | Supplementary Data 6 provides isolate IDs, lineage calls and country of isolation |
| Suppl. Fig. 17 | Supplementary Data 6 | - | Supplementary Data 6 provides isolate IDs, lineage calls and country of isolation |
| Suppl. Fig. 18 | Supplementary Data 6 | - | Supplementary Data 6 provides isolate IDs, lineage calls and country of isolation |
| Suppl. Fig. 19 | Supplementary Data 6 | - | Supplementary Data 6 provides isolate IDs, lineage calls and country of isolation |
| Suppl. Fig. 20 | tree_all_isolates_curated_phenotypes_dataset.treefile | - | Phylogenetic tree for all the isolates of the dataset with phenotypic resistance data |
| Suppl. Fig. 21 | tree_all_isolates_curated_phenotypes_dataset.treefile | - | Phylogenetic tree for all the isolates of the dataset with phenotypic resistance data |
| Suppl. Fig. 22 | Source Data XLSX.xlsx | prop_isolates_snp_thr | Proportion of isolates from each one of the four major lineages (L1-4) found at different SNS difference thresholds |
| Suppl. Fig. 23 | tree_all_isolates_curated_phenotypes_dataset.treefile, Source Data XLSX.xlsx | pheno_res_lin_breakdown | Phylogenetic tree for all the isolates of the dataset with phenotypic resistance data (tree_all_isolates_curated_phenotypes_dataset.treefile, it contains the values of the terminal branch lengths); list of pan-susceptible isolates (Source Data XLSX.xlsx, sheet pheno_res_lin_breakdown) |
| Suppl. Fig. 24 | tree_zignol_dataset.treefile, Supplementary Data 6 |  | Phylogenetic tree for the isolates of the Zignol et al. dataset (tree_zignol_dataset.treefile, it contains the values of the terminal branch lengths); Data for tree annotation (Supplementary Data 6) |
| Suppl. Fig. 25 | Source Data XLSX.xlsx | fst_int_nodes | F_ST_ (Fixation index), calculated on each internal node of the trees of pan-susceptible isolates. |
